# Functional Degeneracy in Paracoccus denitrificans Pd1222 Is Coordinated via RamB, Which Links Expression of the Glyoxylate Cycle to Activity of the Ethylmalonyl-CoA Pathway

**DOI:** 10.1128/aem.00238-23

**Published:** 2023-06-15

**Authors:** Katharina Kremer, Doreen Meier, Lisa Theis, Stephanie Miller, Aerin Rost-Nasshan, Yadanar T. Naing, Jan Zarzycki, Nicole Paczia, Javier Serrania, Patrick Blumenkamp, Alexander Goesmann, Anke Becker, Martin Thanbichler, Georg K. A. Hochberg, Michael S. Carter, Tobias J. Erb

**Affiliations:** a Department of Biochemistry and Synthetic Metabolism, Max Planck Institute for Terrestrial Microbiology, Marburg, Germany; b Department of Biology, University of Marburg, Marburg, Germany; c Department of Biological Sciences, Salisbury University, Maryland, USA; d Core Facility for Metabolomics and Small Molecule Mass Spectrometry, Max Planck Institute for terrestrial Microbiology, Marburg, Germany; e Center for Synthetic Microbiology (SYNMIKRO), Marburg, Germany; f Bioinformatics and Systems Biology, Justus Liebig University Giessen, Giessen, Germany; g Max Planck Fellow Group Bacterial Cell Biology, Max Planck Institute for Terrestrial Microbiology, Marburg, Germany; h Department of Chemistry, University of Marburg, Marburg, Germany; i Evolutionary Biochemistry Group, Max Planck Institute for Terrestrial Microbiology, Marburg, Germany; Danmarks Tekniske Universitet, The Novo Nordisk Foundation Center for Biosustainability

**Keywords:** Paracoccus denitrificans, anaplerosis, carbon metabolism, ethylmalonyl-CoA pathway, glyoxylate cycle, metabolic regulation, proteobacteria, transcriptional regulation

## Abstract

Metabolic degeneracy describes the phenomenon that cells can use one substrate through different metabolic routes, while metabolic plasticity, refers to the ability of an organism to dynamically rewire its metabolism in response to changing physiological needs. A prime example for both phenomena is the dynamic switch between two alternative and seemingly degenerate acetyl-CoA assimilation routes in the alphaproteobacterium Paracoccus denitrificans Pd1222: the ethylmalonyl-CoA pathway (EMCP) and the glyoxylate cycle (GC). The EMCP and the GC each tightly control the balance between catabolism and anabolism by shifting flux away from the oxidation of acetyl-CoA in the tricarboxylic acid (TCA) cycle toward biomass formation. However, the simultaneous presence of both the EMCP and GC in P. denitrificans Pd1222 raises the question of how this apparent functional degeneracy is globally coordinated during growth. Here, we show that RamB, a transcription factor of the ScfR family, controls expression of the GC in P. denitrificans Pd1222. Combining genetic, molecular biological and biochemical approaches, we identify the binding motif of RamB and demonstrate that CoA-thioester intermediates of the EMCP directly bind to the protein. Overall, our study shows that the EMCP and the GC are metabolically and genetically linked with each other, demonstrating a thus far undescribed bacterial strategy to achieve metabolic plasticity, in which one seemingly degenerate metabolic pathway directly drives expression of the other.

**IMPORTANCE** Carbon metabolism provides organisms with energy and building blocks for cellular functions and growth. The tight regulation between degradation and assimilation of carbon substrates is central for optimal growth. Understanding the underlying mechanisms of metabolic control in bacteria is of importance for applications in health (e.g., targeting of metabolic pathways with new antibiotics, development of resistances) and biotechnology (e.g., metabolic engineering, introduction of new-to-nature pathways). In this study, we use the alphaproteobacterium P. denitrificans as model organism to study functional degeneracy, a well-known phenomenon of bacteria to use the same carbon source through two different (competing) metabolic routes. We demonstrate that two seemingly degenerate central carbon metabolic pathways are metabolically and genetically linked with each other, which allows the organism to control the switch between them in a coordinated manner during growth. Our study elucidates the molecular basis of metabolic plasticity in central carbon metabolism, which improves our understanding of how bacterial metabolism is able to partition fluxes between anabolism and catabolism.

## INTRODUCTION

Acetyl-CoA is an essential intermediate in central carbon metabolism, where it serves as energy and/or carbon source. While acetyl-CoA is oxidized through the tricarboxylic acid (TCA) cycle for NADH and ATP generation, bacteria have evolved different strategies for the assimilation of acetyl-CoA into biomass. Several acetyl-CoA assimilation routes have been described to date (1–12) among them the glyoxylate cycle (2, 3) and the ethylmalonyl-CoA pathway (4–7, 12). The glyoxylate cycle (GC) consists of only two enzymes, isocitrate lyase (Icl) and malate synthase (Ms), which branch off the TCA cycle and are often organized in one operon under joint transcriptional regulation (13). The ethylmalonyl-CoA pathway (EMCP), instead, is a rather complex pathway that involves at least 12 enzymes (4), among them crotonyl-CoA carboxylase/reductase (Ccr). Ccr catalyzes a unique reaction, the reductive carboxylation of crotonyl-CoA into ethylmalonyl-CoA, and is considered the key enzyme of the EMCP (4, 6). In contrast to the GC, genes encoding the EMCP are typically distributed across the host genome (14).

**FIG 1:**
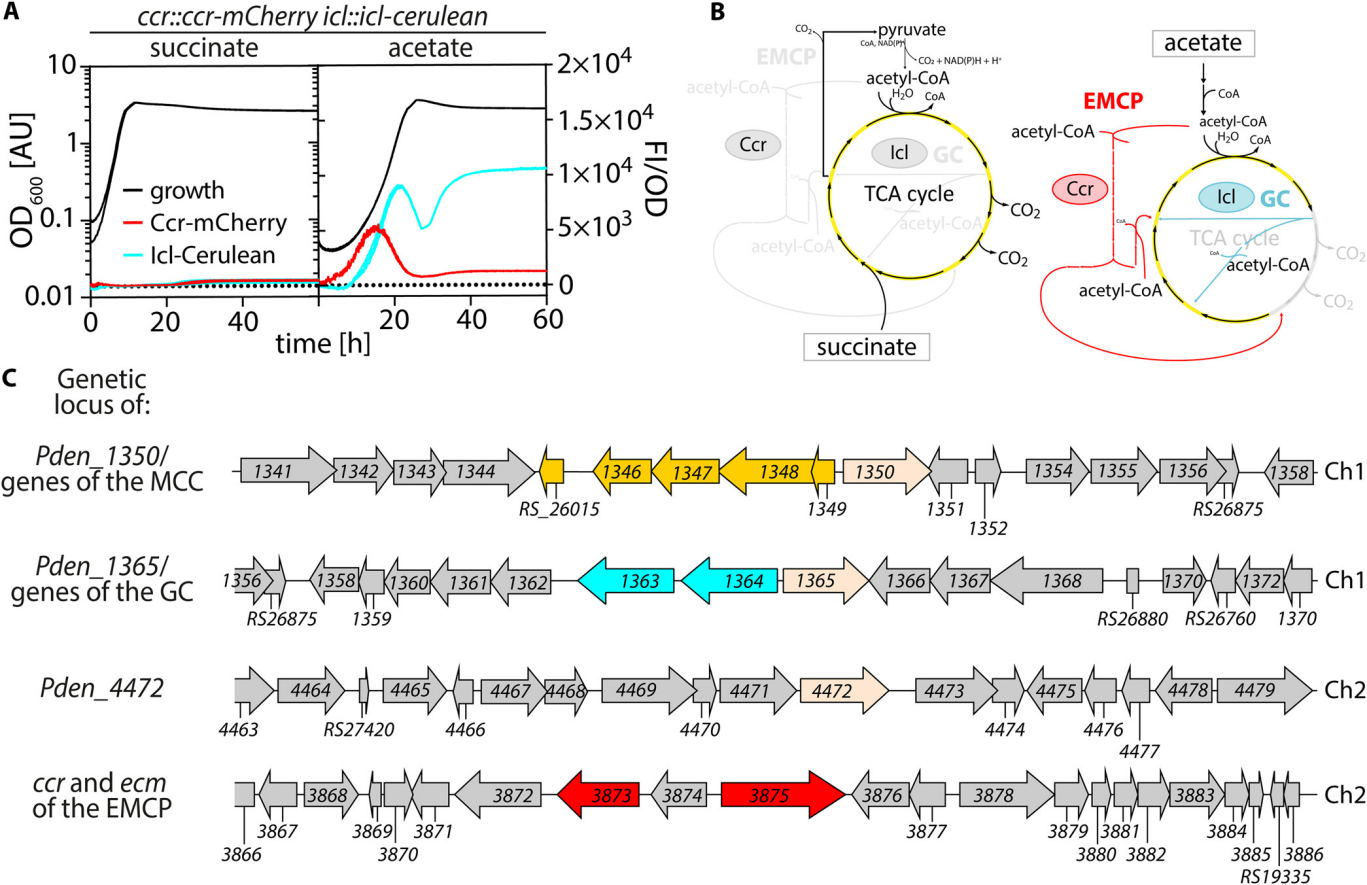
P. denitrificans Pd1222 sequentially employs the EMCP and the GC for efficient assimilation of acetyl-CoA. (A) Growth and fluorescence of Pd1222 *ccr::ccr-mCherry icl::icl-cerulean* (TJE-KK12) on succinate and acetate. Growth is given as OD_600_ on the left *y* axis of each graph. Fluorescence normalized to OD_600_ is given on the right *y* axis of each graph. Replicates are shown as individual curves. (B) Assumed assimilation routes for succinate (left) and acetate (right) in Pd1222. Succinate is assimilated via the TCA cycle. The regeneration of acetyl-CoA from succinate could occur by decarboxylation of malate to pyruvate and subsequent oxidative decarboxylation by pyruvate dehydrogenase (Pdh). The assimilation of acetate requires the activity of the EMCP and GC. (C) Genetic neighborhood of *scfR* homologs as well as the genes encoding the key enzymes of the GC and EMCP. Ch1, chromosome 1; Ch2, chromosome 2. Genes of ScfR homologs (light orange), the MCC (dark orange), the GC (cyan), and the EMCP (red) are highlighted in color. Gene names are given as Pd1222 gene tags (Pden_). For a detailed description of the individual genes of interest and their products, see Table 1.

Despite their biochemical and genetic differences, the EMCP and the GC seem to serve a functionally degenerate purpose; both pathways use three or two molecules of acetyl-CoA, respectively, and bypass the decarboxylation steps of the TCA cycle to allow the cell to form the TCA cycle intermediates succinate and malate from acetyl-CoA (2–5, 7, 12; Fig. 1B; Table 1). This shifts the function of the TCA cycle from acetyl-CoA oxidation (i.e., energy generation) toward acetyl-CoA assimilation (i.e., biomass formation). For optimal growth, bacteria require a tight and fine-tuned regulation of flux between acetyl-CoA oxidation and assimilation (15).

**TABLE 1:** Genes relevant to this study and their corresponding locus tags as well as protein IDs

Pathway	Gene	Product description	Old locus tag	New locus tag	Protein ID
EMCP	*phaA*	β-ketothiolase	Pden_2026	PDEN_RS10080	WP_011748315.1
	*phaB*	Acetoacetyl-CoA reductase	Pden_2027	PDEN_RS10085	WP_011748316.1
	*ccr*	Crotonyl-CoA carboxylase/reductase	Pden_3873	PDEN_RS19270	WP_011750106.1
	*epi*	Epimerase	Pden_2178	PDEN_RS10820	WP_011748465.1
	*ecm*	Ethylmalonyl-CoA mutase	Pden_3875	PDEN_RS19280	WP_041530425.1
	*mcd*	Methylsuccinyl-CoA dehydrogenase	Pden_2840	PDEN_RS14140	WP_011749115.1
	*mch*	Mesaconyl-CoA hydratase	Pden_0566	PDEN_RS02820	WP_011746911.1
	*mcl-1*	β-methylmalyl-CoA/L-malyl-CoA lyase	Pden_0799	PDEN_RS03970	WP_041529775.1
	*mcl-2*	Malyl-CoA thioesterase	Pden_0563	PDEN_RS02805	WP_011746908.1
	*pccA*	Propionyl-CoA Carboxylase subunit A	Pden_3684	PDEN_RS18285	WP_011749920.1
	*pccB*	Propionyl-CoA Carboxylase subunit B	Pden_3688	PDEN_RS18310	WP_011749924.1
	*mcm*	Methylmalonyl-CoA	Pden_3681	PDEN_RS18265	WP_011749917.1
MCC	*prpD*	2-methylcitrate dehydratase	Pden_1348	PDEN_RS06660	WP_011747670.1
	*prpC*	2-methylcitrate synthase	Pden_1347	PDEN_RS06655	WP_011747669.1
	*prpB*	2-methylisocitrate lyase	Pden_1346	PDEN_RS06650	WP_041530157.1
	*acyl-CoA thioesterase*	Acyl-CoA thioesterase	Pden_1349	PDEN_RS06665	WP_011747671.1
	*transcriptional repressor*	Transcriptional repressor	NA	PDEN_RS26015	NA
GC	*Icl (aceA)*	Isocitrate lyase	Pden_1363	PDEN_RS06725	WP_086000156.1
	*ms (aceB)*	Malate synthase	Pden_1364	PDEN_RS06730	WP_011747683.1
	*ramB*	Regulator of acetate metabolism	Pden_1365	PDEN_RS06735	WP_011747684.1
Role not verified	Putative *mccR*	Regulator of methylcitrate cycle	Pden_1350	PDEN_RS06670	WP_011747672.1
	Putative *pccR*	Regulator of methylmalonyl-CoA pathway	Pden_4472	PDEN_RS22285	WP_011750697.1

Among various bacteria, different regulatory mechanisms have been described that regulate flux distribution between acetyl-CoA oxidation and assimilation by controlling the enzymes of the TCA cycle as well as the key enzymes of the GC or EMCP, respectively, at the gene and/or protein level (16–20). However, these studies primarily focused on organisms that possess only one of the two alternative acetyl-CoA assimilation pathways, mostly the GC (summarized in 13). Notably, several organisms exist that harbor both the GC and the EMCP (14, 21–23).

These organisms are not only faced with the challenge to distribute metabolic flux between acetyl-CoA oxidation and assimilation, but also with the question of how to coordinate two seemingly degenerate metabolic pathways without compromising fitness.

While “functional degeneracy” describes the phenomenon that cells can use and metabolize the same substrate through different pathways (1), “metabolic plasticity” refers to the ability of a cell to dynamically rewire metabolic routes in response to changing physiological needs (24). The latter is well known from cancer cells. There, metabolic plasticity mediates survival and metastatic outgrowth by facilitating the rapid adaptation to changing conditions, which enables cancer cells to outcompete their neighbors by ensuring optimal nutrient supply (25–29). Several examples of functional degeneracy and metabolic plasticity have been observed in microbial communities (30–33) as well as individual bacterial populations. One example is the presence of two functionally degenerate routes for the oxidation of methylamine in Methylobacterium extorquens AM1 (34, 35) or the simultaneous existence of the EMCP and GC in several alphaproteobacteria such as P. denitrificans Pd1222 (22) or Rhodobacter capsulatus (23). Yet, the molecular mechanisms coordinating functional degeneracy and metabolic plasticity in bacteria remain largely unknown.

Here, we study the regulation of functional degeneracy during acetyl-CoA metabolism in P. denitrificans Pd1222. We show that acetyl-CoA metabolism follows a dedicated pattern in which the EMCP and GC are sequentially upregulated during a switch to acetate as the sole carbon source for growth. Using a combination of genetic, molecular biological, and biochemical approaches, we identified the transcriptional regulator controlling the GC in P. denitrificans Pd1222, characterized its DNA-binding site and identified CoA esters as small-molecule ligands that induce a change in the oligomerization state of the protein. Notably, these CoA esters are direct intermediates of the EMCP, implying that the expression of the GC is directly linked to an active EMCP. Altogether, our study demonstrates an as of yet unknown regulatory mechanism in central carbon metabolism, in which one seemingly degenerate pathway drives the expression of another.

## RESULTS

### The ScfR family of transcription factors are potential regulators of acetate metabolism in P. denitrificans Pd1222.

To study the regulation of the GC and the EMCP during a switch from succinate to acetate as the sole carbon source for growth, we used the reporter strain P. denitrificans Pd1222 *ccr::ccr-mCherry icl::icl-cerulean*, in which the key enzymes of the EMCP (Ccr) and the GC (Icl) were fused to distinct fluorescent proteins. During growth on succinate, neither Ccr-mCherry nor Icl-Cerulean fluorescence was detected (Fig. 1A). Upon a switch to acetate, the Ccr-mCherry signal increased first followed by the Icl-Cerulean signal. This sequential activation of key enzymes of the EMCP (Ccr) and the GC (Icl) is in line with earlier reports (22) that additionally showed very basal levels of Ccr (and no Icl) activity in cell extracts of succinate-grown cells and subsequent induction of both enzymes on acetate (Fig. 1A and B).

We next aimed to identify potential regulators of acetate metabolism in P. denitrificans Pd1222, henceforth termed Pd1222. Notably, several well-known homologs of transcriptional (e.g., IclR (18)) and posttranslational (e.g., AceK) (16, 17) regulators of the GC are absent in Pd1222, indicating that the regulation of this pathway relies on other mechanisms. The family of short-chain fatty acid regulators (ScfR) drew our attention, as several of these proteins are involved in the regulation of central carbon metabolic pathways, in particular acetate and/or propionate assimilation (36). Three known ScfR family members are encoded in the genome of Pd1222 (36), namely, Pden_4472, Pden_1365, and Pden_1350. Based on sequence similarity and gene neighborhood analysis, Pden_1350 is suggested as regulator of the methylcitrate cycle (MccR) and Pden_1365 as regulator of acetate metabolism (RamB), while no specific function could be predicted for Pden_4472 due to its genomic isolation (Fig. 1C) (36).

We used the enzyme similarity tools EFI-EST (37) and Cytoscape (38) to construct a sequence similarity network for ScfR family members in Pd1222 (Fig. S1). In line with previous predictions (36), Pden_1350 clustered together with PrpR, a recently characterized MccR homolog from Mycobacterium tuberculosis (39, 40), while Pden_1365 clustered in a node of RamB-like proteins that belong to subclass 2 and are distinct from the archetypal Corynebacterium glutamicum RamB that defines subclass 1 (36, 41). Pden_4472 was located in a node together with the regulator of propionyl-CoA carboxylase (PccR) that has recently been characterized as transcriptional activator of the methylmalonyl-CoA pathway (MMCP) in Rhodobacter sphaeroides (Fig. S1) (36). Notably, the MMCP is part of the canonical EMCP where it converts propionyl-CoA into succinyl-CoA. Two additional proteins of Pd1222 appeared in our ScfR network, namely, Pden_1785 and Pden_2985. The corresponding genes clustered together with genes for a two-component system and an isocitrate lyase/phosphoenolpyruvate mutase family protein, respectively. Based on the sequence similarity network analysis, we focused on Pden_1350, Pden_1365 and Pden_4472 as potential regulators of acetate metabolism in the following.

### Pden_1365 is a RamB homolog that regulates acetate metabolism in Pd1222.

**FIG 2:**
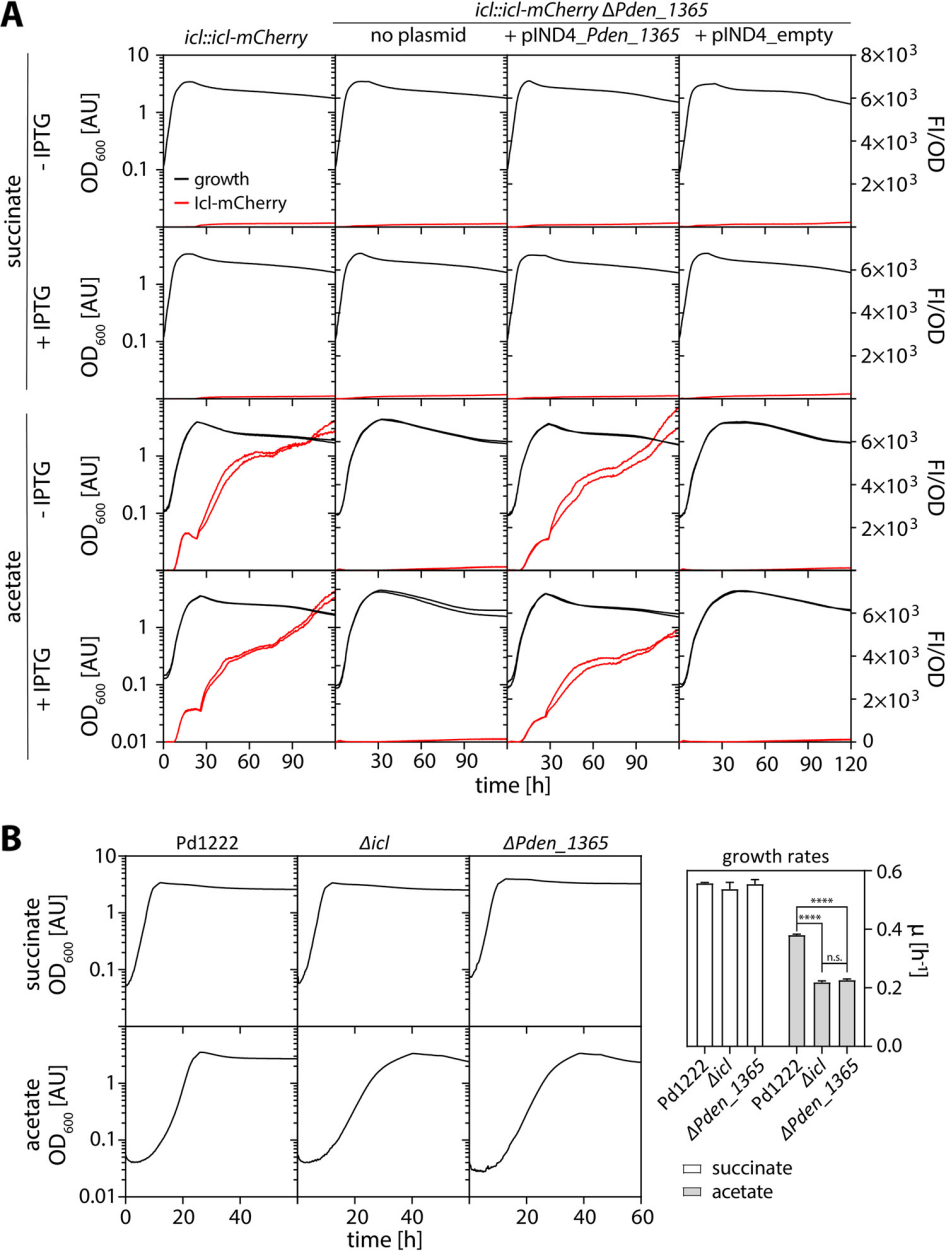
RamB (Pden_1365) is required for efficient production of Icl in P. denitrificans Pd1222. (A) Growth and fluorescence of Pd1222 *icl::icl-mCherry* (TJE-KK3), Pd1222 *icl::icl-mCherry* Δ*Pden_1365* (TJE-KK16), Pd1222 *icl::icl-mCherry* Δ*Pden_1365* pIND4_flag-Pden_1365 (TJE-KK43), and Pd1222 *icl::icl-mCherry* Δ*Pden_1365* pIND4_empty (TJE-KK48) on succinate and acetate in the absence (-) and presence (+) of 0.5 mM IPTG. Growth is given as OD_600_ on the left *y* axis. Fluorescence normalized to OD_600_ is given on the right *y* axis. Replicates are shown as individual curves. (B) Growth of Pd1222, Pd1222 Δ*icl* (TJE-KK8) and Pd1222 Δ*Pden_1365* (TJE-KK19) on succinate (upper panels) and acetate (lower panels). Replicates are shown as individual curves. The corresponding growth rates are given on the right. Asterisks indicate the level of significance as determined by *t* test. ****, *P* < 0.0005; n.s., not significant.

To test whether one or several of the ScfRs identified were involved in the regulation of the EMCP or GC, we studied the function of Pden_1350, Pden_1365, and Pden_4472 in Pd1222 *in vivo*. To this end, we deleted each of these genes individually in Pd1222 reporter strains with *ccr-mCherry* or *icl-mCherry/icl-cerulean* fusions. Neither the deletion of *Pden_4472* nor that of *Pden_1350* affected growth or pathway expression patterns in any of the reporter strains under any condition tested (Fig. S2A and B), which excludes these genes as regulators of the GC or the upper part of the EMCP in Pd1222. In contrast, and in line with its proposed function, the deletion of *Pden_1365* abolished the acetate-triggered production of Icl-mCherry in the *icl::icl-mCherry* reporter background and reduced the growth rate on acetate by 50% compared to the wild-type (WT) strain (μ*_icl::icl-mCherry_* = 0.4 h^−1^ versus μ*_icl::icl-mCherry_*_Δ_*_Pden_1365_* = 0.2 h^−1^) (Fig. 2A, column 2). This phenotype could be complemented by expression of *flag-Pden_1365* from an inducible promoter on plasmid pIND4 (42) (Fig. 2A, column 3), while the empty pIND4 had no effect on growth or fluorescence of the *Pden_1365* knockout reporter strain (Fig. 2A, column 4). Note that plasmid pIND4 is known to show leaky expression in P. denitrificans Pd1222 (42). Accordingly, the Δ*Pden_1365* growth and expression phenotypes could also be complemented by pIND4_*flag-Pden_1365* in the absence of inducer (Fig. 2A, column 3, row 3).

Deletion of *Pden_1365* in the WT background confirmed these results. Similar to the *icl::icl-mCherry* Δ*Pden_1365* strain, the *Pden_1365* deletion in the WT reduced the growth rate on acetate 2-fold (μ_WT_ = 0.4 h^−1^ versus μ_Δ_*_Pden_1365_* = 0.2 h^−1^) (Fig. 2B). Notably, the growth phenotype of the Δ*Pden_1365* mutant resembled that of a Δ*icl* mutant (22) (μ_Δ_*_icl_* = 0.2 h^−1^) (Fig. 2B), suggesting that the growth defect caused by deletion of *Pden_1365* could be caused by a lack of Icl in the cell. Together, these results verified the function of Pden_1365 as regulator of acetate metabolism in Pd1222 and suggested that the protein serves as transcriptional activator for the GC. Therefore, we will refer to Pden_1365 as RamB_Pd_ in the following.

### RamB_Pd_ binds to a conserved motif upstream of the GC operon.

Next, we aimed at identifying the promoter region of GC genes that is targeted by the transcription factor RamB_Pd_. The gene encoding RamB_Pd_ (*Pden_1365*) is located in reverse orientation on chromosome 1, directly upstream of the genes for malate synthase (*Pden_1364; aceB*, in the following referred to as *ms*) and *icl* (*Pden_1363*) (Fig. 1C, row 2). We assessed the upstream region of *ms* as well as the 120 bp intergenic region between *ms* and *icl* in more detail. To that end, we transformed Pd1222 with fluorescence reporter plasmids carrying either the upstream region of *ms* or the *ms-icl* intergenic region fused to a downstream *mCherry* reporter gene (Fig. S3A). In these experiments during growth on acetate, we detected fluorescent expression for the construct containing the *ms* upstream region, whereas no signal was observed for the *ms-icl* intergenic region or a promoterless *mCherry* gene. The fluorescence pattern obtained with the *ms* upstream region (Fig. S3D) was comparable to the signal observed for the Pd1222 *icl::icl-mCherry* strain (see above). However, when we analyzed the reporter construct containing the *ms* upstream region in the Δ*ramB* background (Fig. S3D), we did not observe any fluorescence, supporting the hypothesis that RamB_Pd_ functions as a transcriptional activator. In summary, these results confirmed the role of RamB_Pd_ as the central activator that acts by binding upstream of the *ms* gene.

**FIG 3:**
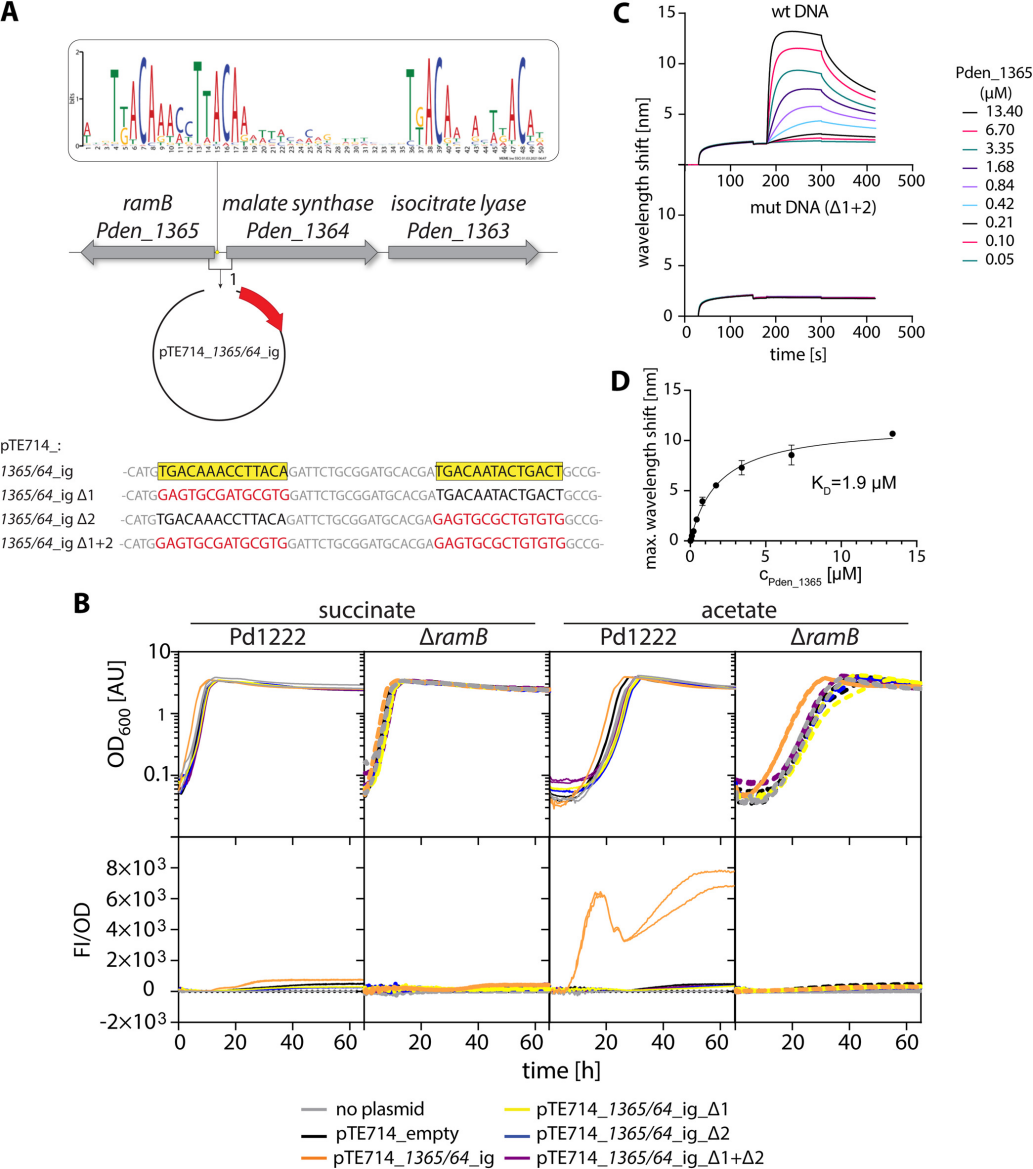
Two binding sites mediate the interaction of RamB_Pd_ with the promoter region of the *gc* operon. (A) Simplified schematic of the *gc* operon in P. denitrificans Pd1222 in reverse complement, including the consensus binding motif as determined by a sequence alignment using the MEME server (43). To generate a reporter construct for *in vivo* assays, the upstream region of the operon, including 50 bp of each flanking open reading frame was integrated upstream of a promoterless *mCherry* gene in the plasmid pTE714, resulting in the plasmid pTE714_1365/64_ig (pTE1634). Shown at the bottom is the sequence of the binding boxes present in the construct as well as the mutated sequences as present in the derivatives pTE714_1365/64_ig_d1 (pTE1637), pTE714_1365/64_ig_d2 (pTE1638), and pTE714_1365/64_ig_d1 + 2 (pTE1639). (B) Growth and fluorescence of Pd1222 and Pd1222 Δ*ramB* containing either no plasmid or different derivatives of pTE714 (as indicated) during growth on succinate and acetate. Growth is given as OD_600_ (upper panels), fluorescence is shown normalized to OD_600_ (lower panels). (C) Biolayer interferometry analysis showing the interaction of RamB_Pd_ with its target DNA *in vitro.* A biotinylated 80 bp dsDNA fragment of the promoter region of the *gc* operon containing intact binding boxes (upper panel) or mutated binding boxes (lower panel; mutations correspond to those present in pTE714_1365/64_ig Δ1 + 2) was immobilized on Sartorius Octet SAX2 biosensors. The biosensor tips with immobilized DNA were probed with increasing concentrations of RamB_Pd_ in binding buffer. Wavelength shifts resulting from an increase in the biolayer due to protein association were monitored over the course of time using a BLItz (FortéBio, USA). (D) Shown are the wavelength shifts reached at equilibrium at the end of the association phase plotted versus the concentration of protein present in the reaction. The K_D_ was determined as the concentration at which the half-maximal wavelength shift was reached.

RamB_Pd_ is a class 2 RamB homolog (36) (Fig. S1). While specific binding motifs are known (or predicted) for class 1 RamB homologs and other classes of ScfR-type transcription factors, no consensus motif has been described for class 2 RamB homologs to date (36). To identify such a binding motif, we searched for genomes that contain class 2 RamB homologs clustering with a putative GC operon. We identified 67 bacterial genomes that fulfilled this requirement and analyzed the sequences upstream of the GC operon with MEME (Multiple Em for Motive election) (43) to determine a putative class 2 RamB binding motif. As is common for ScfR binding sites, this binding motif consists of two individual binding boxes that are nearly identical and separated from each other by an 18 bp spacer (Fig. 3A). In the Pd1222 genome, this motif is located 82 bp upstream of the *ms* gene. We mutated either one or both binding boxes in a reporter plasmid containing the *ramB-ms* intergenic region fused to an *mCherry* reporter gene (Fig. 3A). When introduced into the WT strain, only the reporter construct in which both binding boxes were intact yielded an mCherry signal during growth on acetate, while *mCherry* expression was abolished for constructs where one or both binding boxes were mutated (Fig. 3B). In the Pd1222 Δ*ramB* control strain, none of the constructs showed *mCherry* expression, which is consistent with the notion that RamB_Pd_ functions as a transcriptional activator. We further verified these findings *in vitro* by analyzing the interaction of purified RamB_Pd_ (Fig. S4) with target DNA using biolayer interferometry. To this end, an 80 bp fragment of the *gc* promoter region comprising the two binding boxes separated by an 18 bp spacer and additional 17 bp on each side was immobilized on a biosensor and probed with increasing concentrations of protein, yielding an equilibrium dissociation constant K_D_ of 1.9 μM (Fig. 3C and D). Mutation of both binding boxes resulted in a complete loss of RamB_Pd_ binding (Fig. 3C). Altogether, these experiments identified and confirmed the putative binding site of RamB_Pd_.

### RamB_Pd_ is a transcriptional activator and repressor.

**FIG 4:**
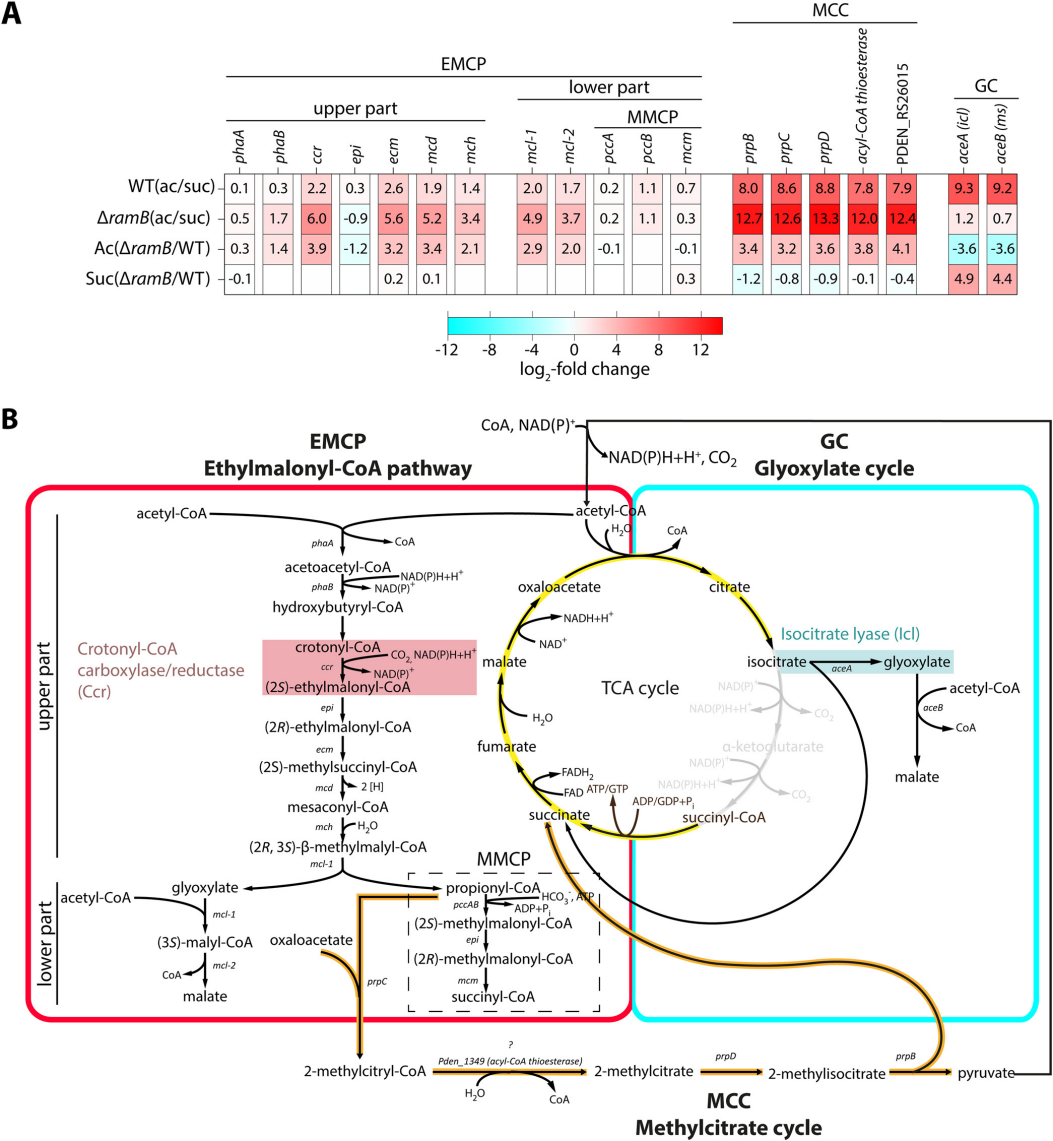
The genes of three pathways are upregulated during growth of P. denitrificans Pd1222 on acetate. (A) Regulation of the genes of the EMCP, GC, and MCC in Pd1222 and Pd1222 Δ*ramB* during mid-exponential growth phase on acetate and succinate. Data shown are derived from RNAseq-based transcriptome analysis. mRNA profiles were determined from triplicate growth cultures. Differential gene expression (DEseq2-based, Table S1 to S4) is given as log_2_ fold change in RNA levels between the two conditions indicated in front of each row. (B) Schematic representation of the pathways relevant for acetyl-CoA assimilation in P. denitrificans Pd1222 and their proposed reaction sequences. Genes coding for the enzymes responsible for catalyzing the individual reactions are given in italics. Color code: TCA cycle (yellow), EMCP (red), GC (cyan), MCC (orange). The key reactions of the EMCP and GC, as catalyzed by Crotonyl-CoA carboxylase/reductase (Ccr) and Isocitrate lyase (Icl), are highlighted with a red and cyan background, respectively. The MMCP (methylmalonyl-CoA pathway) in the lower EMCP is marked by a dashed box.

Next, we analyzed the changes in the global transcriptome of the P. denitrificans Pd1222 WT and the Δ*ramB* mutant induced upon a switch from succinate to acetate as the sole carbon source during mid-exponential growth close to the point at which we expected Icl production in the WT near its maximum (OD_600_ at the time of sampling: 0.8, see Fig. 1A for comparison). In the WT, 43 genes were significantly upregulated under this condition (Fig. S5A; Table S1). These included genes for acetate-CoA ligases (*Pden_4231*, *Pden_4550*, *Pden_4966*), several transporters as well as several enzymes of core metabolism, such as α-ketoglutarate dehydrogenase (*Pden_4984* and *Pden_4985*) or glucose-1-phosphate dehydrogenase (*Pden_4982*); (Table S1). Notably, genes of the *prp*-operon also showed a strong (300-fold) upregulation on acetate (Fig. 4A; Fig. S5A; Table S1). The *prp*-operon encodes the enzymes of the methylcitrate cycle (MCC), which converts propionyl-CoA via methylcitrate and methylisocitrate to succinate and pyruvate (8–11). As propionyl-CoA is an intermediate in the EMCP, upregulation of the MCC indicated that this pathway might be active in parallel to or instead of the MMCP-branch of the EMCP on acetate (Fig. 4B, see Discussion). Interestingly, transcripts of *ramB* showed an average of 10 reads, which is below the limit of statistical significance, indicating a very low, basal expression of *ramB* under all conditions in the WT in line with its proposed function as a transcriptional regulator.

In addition, and as expected in the WT, expression of EMCP and GC genes was also increased upon growth switch to acetate. GC genes were upregulated more than 600-fold on acetate in mid-exponential phase, while EMCP genes were only slightly upregulated (Fig. 4A, row 1; Table S1; Fig. S5A) (see Fig. 1A), which is consistent with the observed decrease of Ccr production in this growth phase. Unlike in the WT, the expression of GC genes did not change significantly in the Δ*ramB* mutant upon a switch to acetate (Fig. 4A, row 2; Fig. S5B; Table S2), indicating that RamB_Pd_ acts as an activator of the *gc* operon. However, while expression of the *gc* operon was not induced in the Δ*ramB* mutant on acetate, it increased more than 20-fold on succinate compared to the WT (Fig. 4A, row 4; Fig. S5C; Table S4), indicating that RamB_Pd_ might serve a dual function as a transcriptional activator on acetate and as a repressor on succinate. This hypothesis is in agreement with the observations that (i) RamB_Pd_ was able to bind its target DNA even in the absence of a potential ligand (Fig. 3C and D); (ii) addition of succinate to an acetate-grown culture in mid-log phase resulted in the downregulation of the GC (Fig. S7A); and (iii) deletion of *ramB* in the *icl::icl-mCherry* background resulted in higher basal mCherry fluorescence on succinate (Fig. S6). Interestingly, EMCP genes were also strongly upregulated in the Δ*ramB* mutant compared to the WT strain, with 8- to 15-fold higher transcript levels of *ccr*, *ecm*, *mcd*, and *mcl-1* compared to the WT on acetate (Fig. 4A, row 3; Table S3; Fig. S2C). Collectively, these results confirmed the central role of RamB_Pd_ as regulator of acetate metabolism in Pd1222 and suggested that the protein can act both as a transcriptional activator and repressor.

### Intermediates of the EMCP bind to RamB_Pd_.

**FIG 5:**
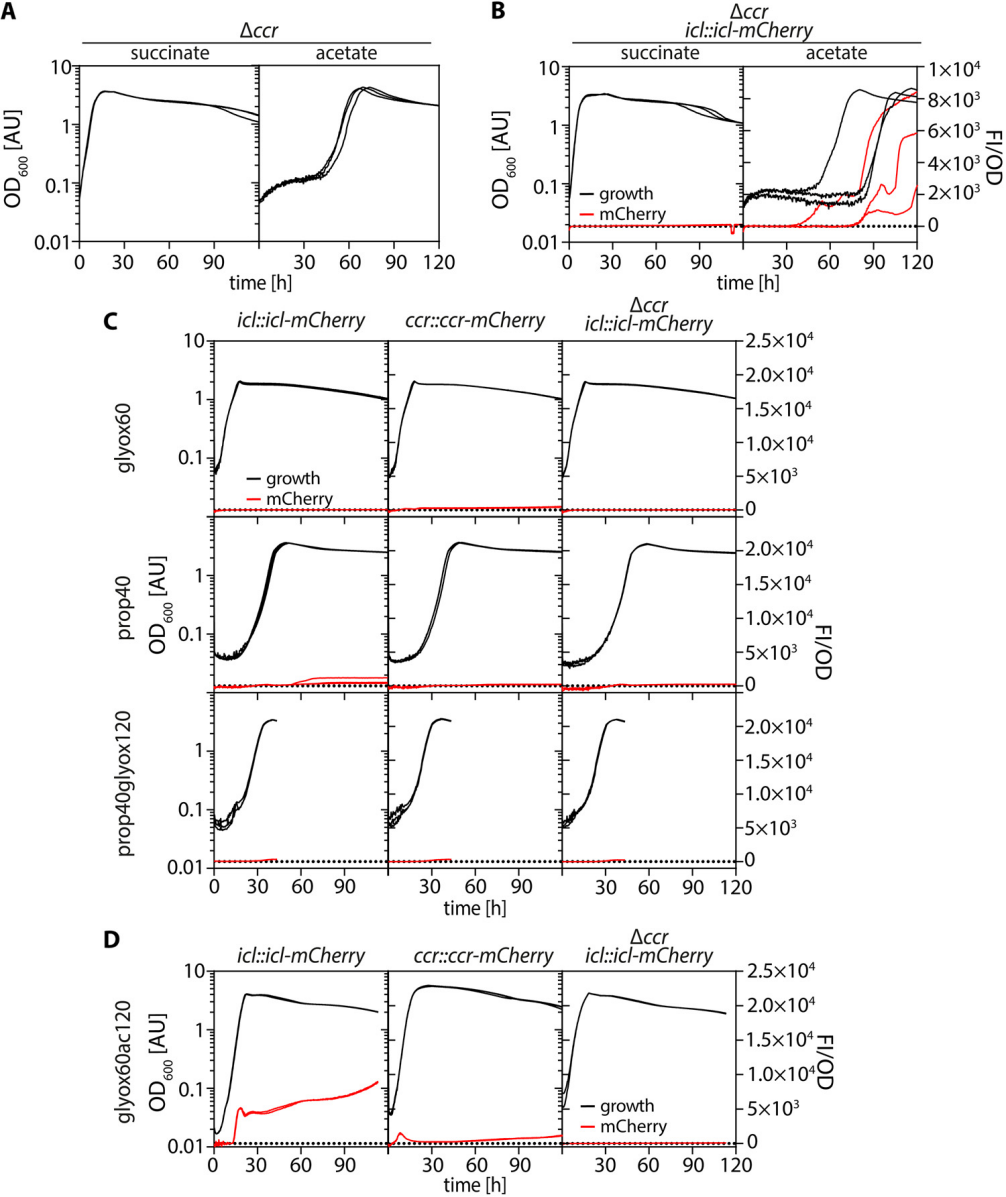
Expression of the GC depends on preceding activity of the EMCP. Growth of different reporter strains of Pd1222 as indicated. Growth (black) is given as OD_600_ on the left y-axes. Fluorescence normalized to OD_600_ is given as on the right y-axes. Replicates are shown as individual curves. (A) Growth of Pd1222 Δ*ccr* (TJE-KK7) on succinate and acetate. (B) Growth of Pd1222 Δ*ccr* *icl*::*icl*-*mCherry* (TJE-KK40) on succinate and acetate. (C) Growth of Pd1222 *icl*::*icl*-*mCherry* (TJE-KK3), Pd1222 *ccr*::*ccr*-*mCherry* (TJE-KK11), Pd1222 Δ*ccr* *icl*::*icl*-*mCherry* (TJE-KK40) on 60 mM glyoxylate (glyox60), 40 mM propionate (prop40) and 40 mM propionate mixed with 120 mM glyoxylate (prop40glyox120). (D) Growth of the strains described in (C) on 60 mM glyoxylate mixed with 120 mM acetate (glyox60ac120).

Previous studies showed that PrpR (from the MccR group of the ScfR family) from M. tuberculosis binds CoA-esters via a dedicated ligand binding pocket in dependence on an intact [4Fe4S] cluster in the protein (39). Homology modeling using SWISS-MODEL (44) with PrpR as template (39) predicted a similar binding pocket as well as the presence of the [4Fe4S] cluster in RamB_Pd_ (Fig. S9), which we confirmed by UV/Vis spectroscopy (see Materials and Methods). Together, this suggested that RamB_Pd_ also binds CoA-esters. Notably, the EMCP produces several CoA-ester intermediates and is always induced before the GC in the pre-exponential phase of Pd1222 cultures on acetate (OD600 < 0.1) regardless of the presence of additional carbon sources in the medium (Fig. 5D; Fig. S8B) or the growth state of the preculture (Fig. S7C), suggesting that an active EMCP and/or its CoA-ester intermediates are required for GC expression. This hypothesis was further supported by the fact that a Δ*ccr* mutant showed a prolonged lag phase (between 30 h to 90 h) upon a switch to acetate (22) (Fig. 5A and B), while the lag phase was unchanged in a Δ*icl* strain under the same conditions (22) (Fig. 2B), suggesting that RamB_Pd_ might indeed sense an EMCP intermediate.

To narrow down the list of potential ligands of RamB_Pd_, we tested whether the signal was an intermediate of the upper part (reactions upstream of Mcl-1) or the lower part (reactions downstream of Mcl-1) of the EMCP (Fig. 4B). Growth on downstream metabolites, such as glyoxylate, propionate, or a mixture of both carbon sources, had no inducing effect on the expression of *ccr* or *icl*, and thus the EMCP or the GC (Fig. 5C). In contrast, growth on upstream substrates such as acetate (Fig. 1A), acetate plus glyoxylate (Fig. 5D), ethanol, or crotonate induced *icl* expression (22), independently of the presence of additional (i.e., 5% vol/vol) CO_2_, which is an important co-substrate of the EMCP and Ccr as well as propionyl-CoA carboxylase (PccAB) in particular (Fig. S7B). Altogether, these results indicated that the signal inducing expression of the GC was produced in the upper part of the EMCP. This notion was further supported by the fact that functional Ccr (and presumably an active EMCP) was required to induce *icl*. While *icl* expression was induced during growth on acetate plus glyoxylate (Fig. 5D, first panel), it was lost under the same conditions in a Δ*ccr* strain (Fig. 5D, last panel).

**FIG 6:**
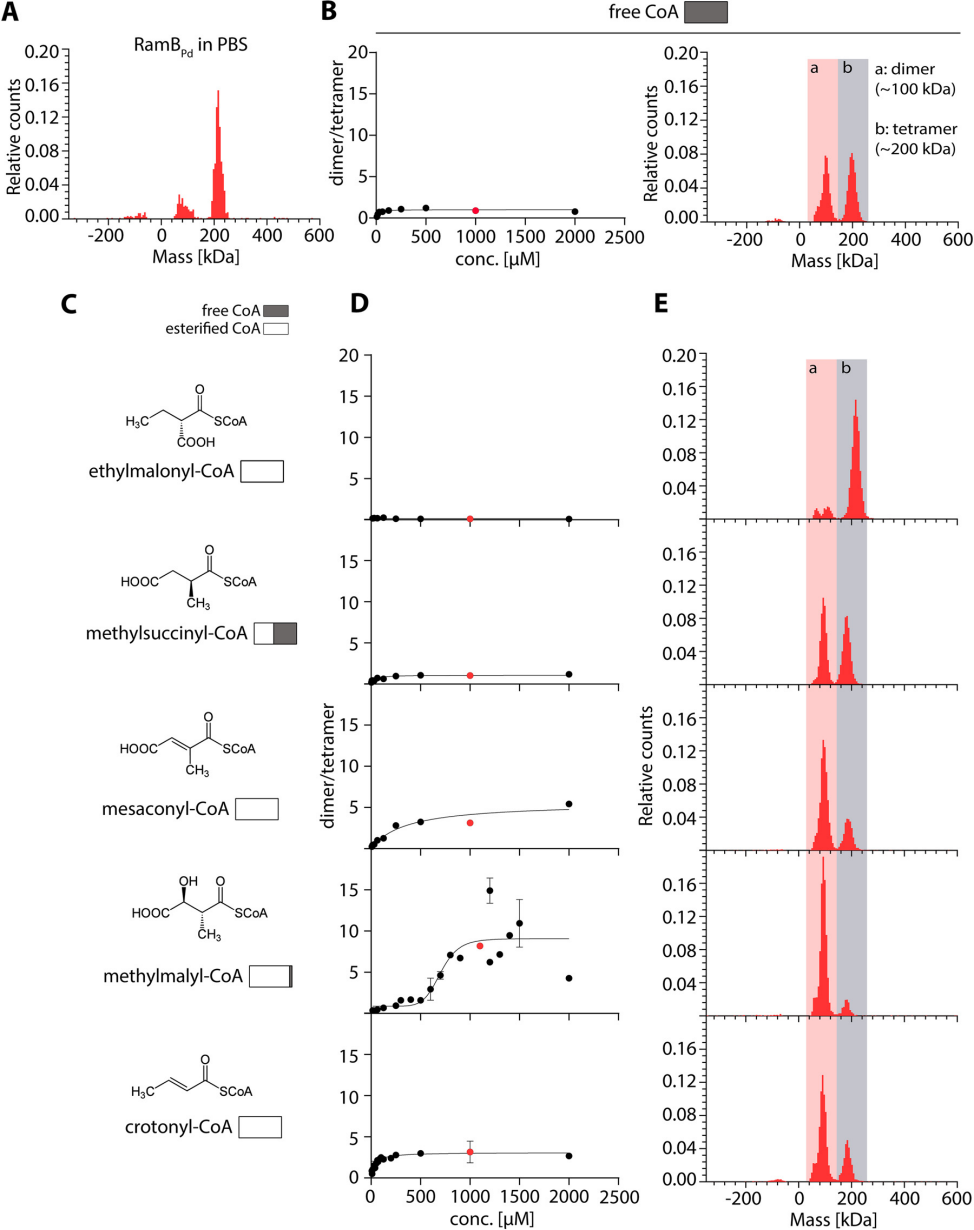
Methylmalyl-CoA induces a change in the oligomeric state of RamB_Pd_. The oligomeric state of RamB_Pd_ was assessed by mass photometry in reactions containing 250 nM protein in PBS buffer with or without supplements as indicated. Relative counts were normalized to the total count number of each measurement. Red dots indicate the assay concentrations, for which results of the MP measurement are exemplified on the right. Dimer peaks are shown on a red background and indicated with (a); tetramer peaks are shown on a gray background and indicated with (b). (A) RamB_Pd_ in PBS buffer. (B) Titration of RamB_Pd_ with rising concentrations of free CoA. RamB_Pd_ was pre-incubated with rising concentrations of free CoA in PBS buffer for 35 min before the measurement. The ratio of dimer/tetramer formed is plotted versus the corresponding concentration of CoA in the assay in triplicate (left panel). Dots represent the mean of three measurements, error bars indicate the standard deviation. An exemplary result of a mass photometry measurement is depicted on the right. (C) Structures and names of the respective CoA-esters used in the assays on the right. The amount of free CoA present in the stock of each CoA-ester is shown as a bar chart with the white area representing the percentage of esterified CoA and the gray area representing the percentage of free CoA. (D) Titration measurements of the interaction of the esters shown in (C) to RamB_Pd_, performed as described in (B). Dots represent the mean of three measurements, error bars indicate the standard deviation. (E) Result of an exemplary mass photometry measurement.

We next aimed to identify the potential ligand of RamB_Pd_. The upper part of the EMCP, including the reactions between the reactions of Ccr and Mcl-1 produces four intermediates, namely (2*S* and 2*R*)-ethylmalonyl-CoA, (2*S*)-methylsuccinyl-CoA, mesaconyl-C1-CoA, and (2*R*/3*S*)-β-methylmalyl-CoA (4, 12) (Fig. 4B). To study the effect of the different-CoA esters on RamB_Pd_, we assessed the oligomeric state of RamB_Pd_ *in vitro* in the absence and presence of the different CoA-esters using mass photometry (45, 46). Besides a background peak at 80 kDa, RamB_Pd_ was detected at a mass corresponding to a molecular weight of approximately 210 kDa (Fig. 6A), indicating that RamB_Pd_ forms a tetramer in solution.

Incubation of the protein in the presence of free CoA resulted in a second peak that increased in size with higher concentrations of CoA, until reaching saturation at 62.5 μM CoA with a peak ratio of 1:1 dimer to tetramer. The protein species in this second peak has a molecular weight of ~105 kDa, corresponding to a dimer. These results suggest that the tetrameric RamB_Pd_ complex dissociates into a dimer upon the addition of CoA. We next tested the influence of the different CoA ester intermediates of the EMCP on dimer formation (Fig. 6C to E). Ethylmalonyl-CoA did not induce dimer formation, while methylsuccinyl-CoA (which contained 54% free CoA) showed similar effects as free CoA. Incubation of RamB_Pd_ with mesaconyl-CoA or crotonyl-CoA resulted in a 3:1 ratio of dimers to tetramers. In contrast, incubation with β-methylmalyl-CoA increased the ratio of dimers to tetramers to 9:1, with an apparent K_D_ of the interaction of 700 μM. The strong shift toward dimer formation at physiologically relevant concentrations suggested that β-methylmalyl-CoA is the preferred ligand of RamB_Pd_.

### Acetate assimilation is different in Rhodobacter capsulatus SB1003.

**FIG 7:**
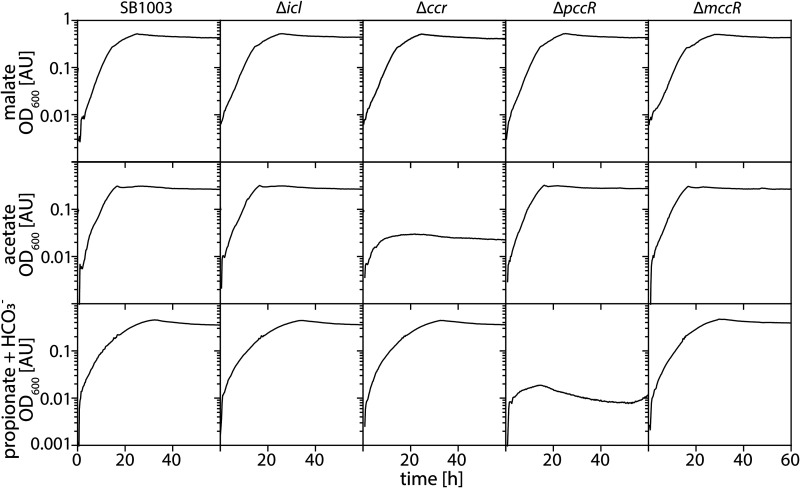
R. capsulatus SB1003 shows different employment patterns of the EMCP and GC. The growth of SB1003 (wt), SB1003 Δ*icl*, SB1003 Δ*ccr*, SB1003 Δ*pccR*, SB1003 Δ*mccR* on malate, acetate and propionate supplemented with HCO_3_- is shown as OD_600_. All carbon sources were provided at a concentration of 10 mM each.

We finally turned our attention to Rhodobacter capsulatus SB1003, an alphaproteobacterium that is closely related to P. denitrificans Pd1222 and possesses the genes of the EMCP, the GC and the MCC as well (47). Like Pd1222, this organism harbors several ScfR family members. Based on their locations in the sequence similarity network (Fig. S1) and the neighborhoods of their respective genes, we identified the proteins as putative RamB (regulator of the GC genes), putative PccR (regulator of propionyl-CoA carboxylase), and putative MccR (regulator of the MCC genes) (36), indicating a similar strategy of R. capsulatus SB1003 to assimilate acetate as Pd1222. To test this hypothesis, we deleted the genes of the key enzymes and (putative) regulators of acetate metabolism in R. capsulatus SB1003 (*ccr*, *icl*, *pccR*, and *mccR* genes were independently deleted) and analyzed the growth of the mutant strains on different carbon sources. Deletion of these genes did not affect the growth of R. capsulatus SB1003 on malate, the negative control (Fig. 7, line 1). As observed for Pd1222, deletion of *ccr* impaired the growth of R. capsulatus SB1003 on acetate (Fig. 7, column 3, line 2), emphasizing the importance of the EMCP for its ability to assimilate acetyl-CoA. However, unlike in Pd1222, deletion of *icl* did not affect the growth of R. capsulatus SB1003 on acetate (Fig. 7, column 2, line 2), indicating that Icl (and likely the GC) plays a minor role in acetyl-CoA assimilation in this species. Deletion of the regulatory *pccR* or *mccR* genes did not affect growth on acetate, but the deletion of *pccR* uniquely impaired growth on propionate. Deletion of *mccR* left growth of R. capsulatus SB1003 on propionate and CO_2_ unaffected, indicating that the MCC was not able to substitute for the loss of the expression of MMCP genes, which requires PccR (Fig. 7, column 4, line 3) (36). In summary, these findings suggested that despite the presence of a common set of pathways and regulators, acetate metabolism strongly differs between these two organisms.

## DISCUSSION

Organisms must balance metabolic flux between catabolic and anabolic routes during growth. One of the key intermediates in central carbon metabolism is acetyl-CoA, which can be either oxidized in the citric acid cycle (catabolism) or assimilated into biomass (anabolism). Notably, the alphaproteobacterium P. denitrificans Pd1222 employs two functionally degenerate acetyl-CoA assimilation pathways, the EMCP and the GC. It has been suggested that the EMCP represents the ancestral pathway in P. denitrificans Pd1222, while the GC was likely acquired through lateral gene transfer (22). The existence of two apparently redundant central metabolic traits in one organism is surprising and raises the question why P. denitrificans Pd1222 uses two parallel acetyl-CoA assimilation strategies and how the expression and activity of the two pathways are coordinated during growth of P. denitrificans Pd1222.

**FIG 8:**
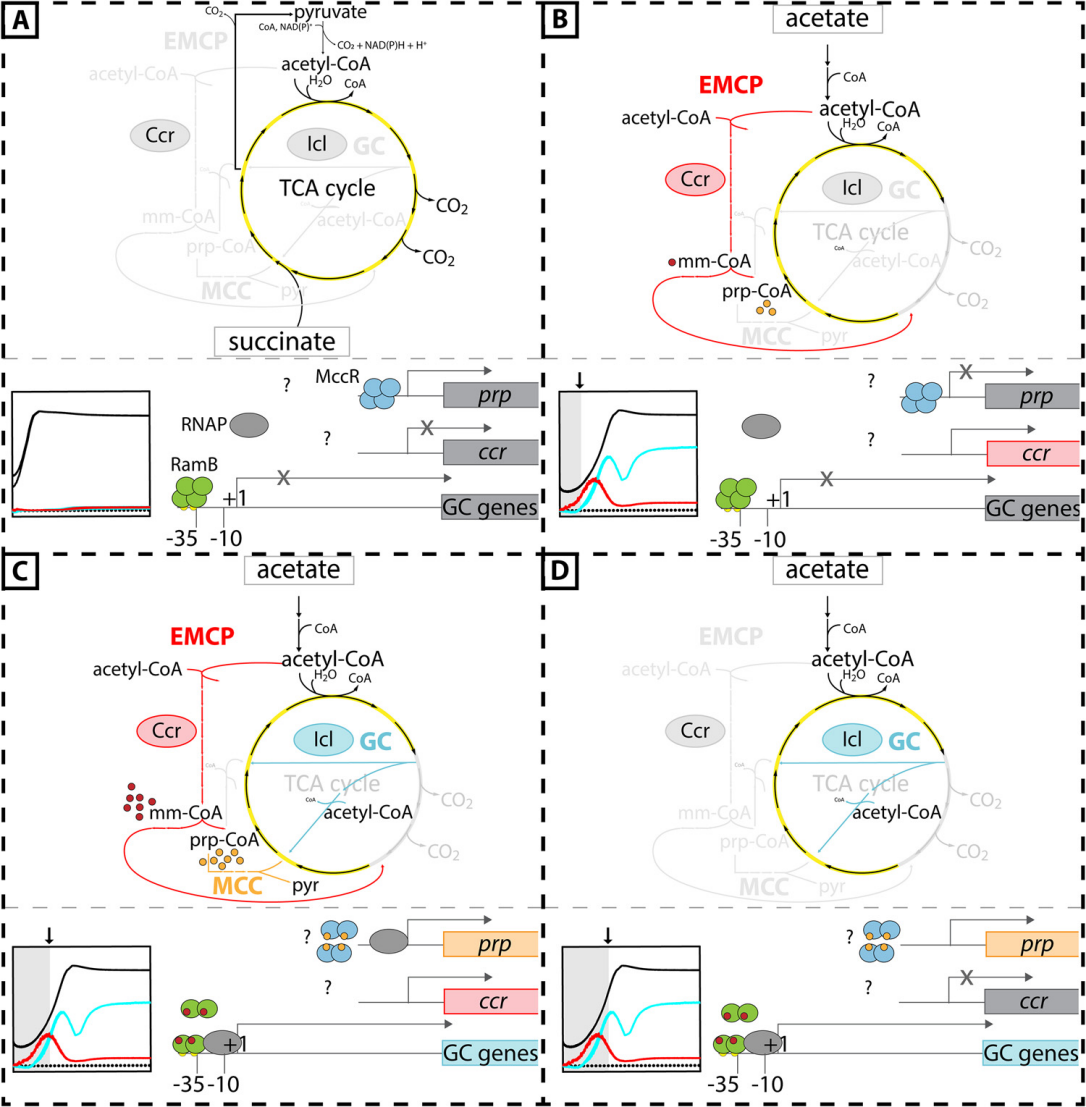
Hypothetical regulation of acetyl-CoA metabolism in P. denitrificans Pd1222. Shown is the interplay of the MCC, the EMCP, and the GC during growth on succinate (A) and growth on acetate (B to D). The different stages of growth on acetate depicted are indicated in the growth curve by arrows and gray background. Regulator binding sites, the -35 and -10 boxes as well as the transcription start site (+1) are shown for the *gc* operon. (A) Assimilation of succinate occurs via the TCA cycle. Neither the genes of the MCC (*prp*-operon), the EMCP (here shown on the example of *ccr*), nor the GC are expressed at relevant levels under this condition. Expression of the *prp* operon might be negatively or positively regulated by MccR (Pden_1350; here shown is an example for negative regulation). Expression of the GC genes is repressed by a blockage of transcription through binding of tetrameric RamB to the *gc* promoter. (B) When the cells are shifted to growth on acetate, genes of the EMCP are initially upregulated. The resulting flux through the EMCP produces the metabolites methylmalyl-CoA (mm-CoA) and propionyl-CoA (prp-CoA). Due to low activity or the lack of propionyl-CoA carboxylase (PccAB) and other propionyl-CoA converting enzymes, propionyl-CoA accumulates, which leads to a backup in the EMCP and consequently an accumulation of methylmalyl-CoA. (C) propionyl-CoA might stimulate MccR to trigger expression of the *prp*-genes, resulting in the metabolization of propionyl-CoA by the now produced MCC enzymes. Binding of methylmalyl-CoA to RamB promotes a decay of the tetrameric inhibitory RamB complex into dimers. In the dimeric form, RamB stays bound to the *gc* promoter, but now promotes expression of the GC genes through interaction with RNA polymerase (RNAP). (D) A yet unknown signal leads to downregulation of the EMCP, while the GC (and presumably the MCC) remains upregulated.

Here, we propose a novel mechanism of transcriptional control of GC genes that involves metabolic cross talk via RamB, a regulator of the ScfR family that binds upstream of the *gc* operon (Fig. 8). Induction of the GC is driven by flux through the EMCP, an alternative pathway for assimilating the same initial substrate.

The hypothesis that RamB links the expression of the GC to the activity of the EMPC is supported by several findings. First, the EMCP is (always) active at very basal levels in P. denitrificans Pd1222 and becomes upregulated in the pre-exponential growth phase after a switch to acetate and prior to induction of the GC. Second, deletion of the EMCP-essential *ccr* gene results in a significantly prolonged lag in GC expression. Third, *in vitro* experiments with purified RamB_Pd_ show that several intermediates of the EMCP, and in particular β-methylmalyl-CoA, cause a shift from the tetrameric to the dimeric state of RamB_Pd_ at physiologically relevant ligand concentrations.

Our findings show that P. denitrificans Pd1222 achieves metabolic plasticity through a complex entanglement and coordination of functionally degenerate central carbon metabolic pathways. Functional degeneracy is not uncommon in prokaryotes, and alphaproteobacteria in particular. Methylobacterium extorquens AM1, a related methylotroph, for instance, features two different methylamine assimilation routes that serve different purposes and confer distinct advantages under different growth conditions (34, 35). In the case of acetyl-CoA assimilation in P. denitrificans Pd1222, the EMCP might serve as a basic assimilation route, which is replaced by the more specialized and more efficient GC, when high fluxes require a strong commitment to acetyl-CoA assimilation (22).

This work clarifies the molecular basis of GC regulation in P. denitrificans Pd1222, but several questions remain unanswered. While upregulation of the GC requires an active EMCP, as shown in this work, the mechanism upregulating the EMCP itself remains enigmatic. In addition, the regulation of acetate metabolism in P. denitrificans Pd1222 is more complex and seems to involve catabolite repression, because succinate suppresses acetate metabolism (Fig. S8) but apparently not EMCP expression. Moreover, externally added succinate directly represses the expression of GC genes. This indicates a (post-)transcriptional/translational feedback loop and points to a more integrated regulation of acetate metabolism in the context of central carbon metabolism in P. denitrificans Pd1222. This hypothesis is further supported by the observation that the deletion of *ramB* or *icl* increases EMCP expression, possibly to compensate for the loss of GC activity.

An interesting finding of our study is that the MCC is upregulated in addition to the EMCP and GC during growth on acetate (and even further increased in the absence of a functional GC in the Δ*ramB* mutant). This indicates that propionyl-CoA might be additionally assimilated through the MCC under these conditions, possibly as a strategy to prevent accumulation of potentially toxic concentrations of propionyl-CoA in the cell (48). In any way, the additional upregulation of the MCC represents an interesting variation of the EMCP that might add to the functional degeneracy of *P. dentrificans* Pd1222 and, potentially, other organisms expressing the EMCP and the MCC.

We would like to note that the functional degeneracy of acetyl-CoA assimilation routes is accompanied by regulatory degeneracy: although R. capsulatus SB1003 possesses in principle the same metabolic capabilities (i.e., codes for the EMCP, GC, and MCC) and homologous regulatory proteins (i.e., RamB, PccR, MccR), the regulation of acetate metabolism (i.e., the induction of the different pathways), and the relevance of functional degeneracy of acetyl-CoA assimilation differs from P. denitrificans Pd1222. Future studies focusing on acetyl-CoA assimilation in *P. dentrificans* Pd1222 and/or R. capsulatus SB1003 will shed more light on and thus eventually complete our picture of functional degeneracy and metabolic plasticity in alphaproteobacteria. These findings are also of importance for efforts to reprogram and rewire bacterial central carbon metabolism in synthetic biology and biotechnology, which aim at introducing new metabolic modules into alphaproteobacteria and/or transplanting alphapreoteobacterial pathways into other model systems (49, 50).

## MATERIALS AND METHODS

### Chemicals.

All chemicals used in this study were obtained from Sigma-Aldrich (Steinheim, Germany) and Carl Roth (Karlsruhe, Germany) unless specified otherwise and were of the highest purity available.

### Strains, medium, and cultivation conditions.

All plasmids and strains used in this study are listed in Table 2. Escherichia coli was grown in Luria Bertani (LB) medium in the presence of appropriate antibiotics (ampicillin [100 μg/mL]; gentamycin [30 μg/mL]; kanamycin [50 μg/mL]; streptomycin [20 μg/mL]; tetracycline [10 μg/mL]) at 37°C. For the cultivation of E. coli ST18, media were supplemented with 5-aminolevulinic acid (50 μg/mL). Paracoccus denitrificans was grown in the presence of appropriate antibiotics (kanamycin [50 μg/mL]; spectinomycin [50 μg/mL]; rifampicin [30 μg/mL], tetracycline [10 μg/mL]) in LB medium or mineral salt medium supplemented with trace elements TE3-Zn (51) and defined carbon compounds at a total carbon concentration of 120 mM at 30°C. For strains carrying derivatives of the suicide plasmid pK18mobsacB, LB medium was replaced with super optimal broth (SOB) to lower the risk of point mutations in the *sacB* gene. R. capsulatus was grown at 30°C with peptone yeast extract (PYE) medium, as well as a modified version of minimal media (7) that, undiluted, contained 20× salt solution (20 g/L NH_4_Cl, 6 g/L MgSO_4_·7H_2_O, 3 g/L KCl, 0.1 g/L CaCl_2_·2H_2_O, 0.05 g/L FeSO_4_·7H_2_O), 20× phosphate buffer, pH 7.0 (500 mM KH_2_PO_4_, 500 mM Na_2_HPO_4_), 1,000× trace elements solution (0.5 g/L NaEDTA, 0.3 g/L FeSO_4_·7H_2_O, 0.003 g/L MnCl_2_·4H_2_O, 0.005 g/L CoCl_2_·6H_2_O, 0.001 g/L CuCl_2_·2H_2_O, 0.002 g/L NiCl_2_·6H_2_O, 0.003 g/L Na_2_MoO_4_·2H_2_O, 0.005 ZnSO_4_·7H_2_O, 0.002 g/L H_3_BO_3_), and 1,000× vitamin solution (0.1 g/L cyanocobalamin, 0.3 g/L pyridoxamine-2 HCl, 0.1 g/L calcium-D(+)-pantothenate, 0.2 g/L thiamine dichloride, 0.2 g/L nicotinic acid, 0.08 g/L 4-aminobenzoic acid, 0.02 g/L, D(+)-biotin) with defined carbon sources as indicated for individual experiments at a concentration of 10 mM each. The optical density at 600 nm (OD_600_) of cultures was determined using a Spectroquant Prove 300 spectrophotometer (Merck, Germany). Growth and fluorescence measurements on P. denitrificans Pd1222 and its derivatives were performed in black VisiView 96-well plates with an optical bottom (Perkin Elmer, USA) in a Tecan M200 Pro plate reader system (Tecan, Switzerland). Growth was followed by measuring the OD_600_ at regular intervals. The fluorescence of mCherry was measured at an emission wavelength of 610 nm after excitation at 575 nm with a constant gain of 120. The fluorescence of Cerulean was measured at an emission wavelength of 475 nm after excitation at 433 nm with a constant gain of 90. The growth of R. capsulatus SB1003 and its derivatives was followed in a SpectraMax i3 plate reader (Molecular Devices, USA) by monitoring the OD_600_.

**TABLE 2:** Strains and plasmids used in this study

Strain/plasmid name	Organism/backbone	Genotype/description	Resistance	Source/reference
TOP10	Escherichia coli	F^–^*mcr*A Δ(*mrr*-*hsd*RMS-*mcr*BC) φ80*lac*ZΔM15 Δ*lac*X74 *rec*A1 *ara*D139 Δ(*ara-leu*)7697 *gal*U *gal*K λ^–^*rps*L(Str^R^) *end*A1 *nup*G		Invitrogen, USA
DH5α	Escherichia coli	F^–^ φ80*lac*ZΔM15 Δ(*lac*ZYA-*arg*F)U169 *rec*A1 *end*A1 *hsd*R17 (r_K_^–^, m_K_^+^) *pho*A *sup*E44 λ^–^*thi*-1 *gyr*A96 *rel*A1		New England Biolabs
S17-1	Escherichia coli	*pro, res^−^ hsdR17 (rK^−^ mK^+^) recA^−^* with an integrated *RP4-2-Tc::Mu-Km::Tn7*, Tp^r^		65
ST18	Escherichia coli	S17 λ*pir*Δ*hemA*		52
BL21 AI	Escherichia coli	F^–^*omp*T *hsd*S_B_ (r_B_^–^, m_B_^–^) *gal dcm ara*B::T7RNAP-*tet*A		Novagen, Germany
Rosetta (DE3)pLysS	Escherichia coli	F- *ompT hsdS*_B_(r_B_- m_B_-) *gal dcm* (DE3) pLysSRARE	Cam	Novagen, Germany
Pd1222	Paracoccus denitrificans	Wild-type with increased conjugation efficiency	Spec, Rif^*a*^	66
TJE-KK3	Paracoccus denitrificans	Pd1222 *icl::icl-mCherry*	Spec, Rif	22
TJE-KK7	Paracoccus denitrificans	Pd1222 Δ*ccr*	Spec, Rif	22
TJE-KK8	Paracoccus denitrificans	Pd1222 Δ*icl*	Spec, Rif	22
TJE-KK11	Paracoccus denitrificans	Pd1222 *ccr::ccr-mCherry*	Spec, Rif	22
TJE-KK12	Paracoccus denitrificans	Pd1222 *ccr::ccr-mCherry icl::icl-cerulean*	Spec, Rif	22
TJE-KK15	Paracoccus denitrificans	Pd1222 *ccr::ccr-mCherry icl::icl-cerulean* Δ*Pden_4472*	Spec, Rif	this work
TJE-KK16	Paracoccus denitrificans	Pd1222 *icl::icl-mCherry* Δ*ramB*	Spec, Rif	this work
TJE-KK17	Paracoccus denitrificans	Pd1222 *ccr::ccr-mCherry* Δ*Pden_1350*	Spec, Rif	this work
TJE-KK18	Paracoccus denitrificans	Pd1222 *icl::icl-mCherry* Δ*Pden_4472*	Spec, Rif	this work
TJE-KK20	Paracoccus denitrificans	Pd1222 *ccr::ccr-mCherry icl::icl-cerulean* Δ*ramB*	Spec, Rif	this work
TJE-KK23	Paracoccus denitrificans	Pd1222 pTE714	Spec, Rif	this work
TJE-KK24	Paracoccus denitrificans	Pd1222 Δr*amB* pTE714	Spec, Rif	this work
TJE-KK32	Paracoccus denitrificans	Pd1222 Δ*ramB* pTE714_*1365/1364*_ig	Spec, Rif	this work
TJE-KK33	Paracoccus denitrificans	Pd1222 pTE714_*1365/1364*_ig	Spec, Rif	this work
TJE-KK40	Paracoccus denitrificans	Pd1222 Δ*ccr icl::icl-mCherry*	Spec, Rif	this work
TJE-KK42	Paracoccus denitrificans	Pd1222 *icl::icl-mCherry* pIND4_FLAG-r*amB*	Spec, Rif	this work
TJE-KK43	Paracoccus denitrificans	Pd1222 *icl::icl-mCherry* Δ*ramB* pIND4_FLAG-*ramB*	Spec, Rif	this work
TJE-KK47	Paracoccus denitrificans	Pd1222 *icl::icl-mCherry* Δ*ramB* pIND4	Spec, Rif	this work
TJE-KK51	Paracoccus denitrificans	Pd1222 pTE714_*1365/64*_ig_d1	Spec, Rif, Tet	this work
TJE-KK52	Paracoccus denitrificans	Pd1222 pTE714_*1365/64*_ig_d2	Spec, Rif, Tet	this work
TJE-KK53	Paracoccus denitrificans	Pd1222 pTE714_*1365/64*_ig_d1 + 2	Spec, Rif, Tet	this work
TJE-KK54	Paracoccus denitrificans	Pd1222 Δ*ramB* pTE714_*1365/64*_ig_d1	Spec, Rif, Tet	this work
TJE-KK55	Paracoccus denitrificans	Pd1222 Δ*ramB* pTE714_*1365/64_*ig_d2	Spec, Rif, Tet	this work
TJE-KK56	Paracoccus denitrificans	Pd1222 Δ*ramB* pTE714_*1365/64*_ig_d1 + 2	Spec, Rif, Tet	this work
TJE-KK57	Paracoccus denitrificans	Pd1222 pTE770	Spec, Rif, Tet	this work
TJE-KK58	Paracoccus denitrificans	Pd1222 Δ*ramB* pTE770	Spec, Rif, Tet	this work
TJE-KK60	Paracoccus denitrificans	Pd1222 icl::icl-mCherry Δ*ramB* Δ*Pden*_4472	Spec, Rif, Tet	this work
SB1003	Rhodobacter capsulatus	Wild-type		this work
Δ*ccr*	Rhodobacter capsulatus	SB1003 Δ16020		this work
Δ*icl*	Rhodobacter capsulatus	SB1003 Δ16495		this work
Δ*pccR*	Rhodobacter capsulatus	SB1003 Δ04520		this work
Δ*mccR*	Rhodobacter capsulatus	SB1003 Δ16525		this work
pTB146		backbone for one of *his-sumo*-tagged genes	Amp	64
pTB145		*his-ulp1*	Amp	64
pK18mobsacB		backbone for double homologous recombination	Kan	67
pTE714		pTE100	Tet	68
pIND4		backbone for one of *his*-tagged genes in alphaproteobacteria	Kan	42
Plasmids for Paracoccus denitrificans Pd1222				
pTE770		pTE714_*mCherry*	Tet	68
pTE1615	pK18mobsacB	pK18mobsacB_*icl*-flanks	Kan	22
pTE1616	pK18mobsacB	pK18mobsacB_*icl-mCherry-iclds*	Kan	22
pTE1630	pK18mobsacB	pK18mobsacB_*Pden1350*-flanks	Kan	this work
pTE1631	pK18mobsacB	pK18mobsacB_P*den1365*-flanks	Kan	this work
pTE1632	pK18mobsacB	pK18mobsacB_*Pden4472*-flanks	Kan	this work
pTE1633	pTE714	pTE714_*1364/1363_*ig	Tet	this work
pTE1634	pTE714	pTE714_*1365/1364_*ig	Tet	this work
pTE1637	pTE714	pTE714_*1365/1364_*ig_d1	Tet	this work
pTE1638	pTE714	pTE714_*1365/1364_*ig_d2_new	Tet	this work
pTE1639	pTE714	pTE714_*1365/1364_*ig_d1 + 2_new	Tet	this work
pTE5000	pIND4	pIND4_*FLAG-ramB* (original *his*-tag of pIND4 was removed in this construct)	Kan	this work
Plasmids for Escherichia coli				
pTE5007	pTB146	pTB146_r*amB*	Amp	this work
Plasmids for Rhodobacter capsulatus SB1003				
pJM009	pK18mobsacB	pK18mobsacB_*ccr*-flanks	Kan	this work
pYN011	pK18mobsacB	pK18mobsacB_*icl-*flanks	Kan	this work
pYN019	pK18mobsacB	pK18mobsacB_*pccR*-flanks	Kan	this work
pRK005	pK18mobsacB	pK18mobsacB_*mccR-*flanks	Kan	this work

a Spec, spectinomycin; Rif, rifampicin; Tet, tetracyclin; Kan, kanamycin; Amp, ampicillin; Cam, chloramphenicol.

### Genetic manipulation of P. denitrificans.

The transfer of plasmids to P. denitrificans was achieved by biparental mating using E. coli ST18 as a donor strain (52). The deletion of genes from the P. denitrificans genome was carried out as described (22) with LB medium being replaced with SOB medium. The transfer of replicative plasmids to P. denitrificans was done according (53). The selection of transformants occurred on mineral salt medium supplemented with 120 mM methanol and appropriate antibiotics. The successful transfer of plasmids and the deletion of genes were verified by colony PCR. All strains and plasmids used in this study are listed in Table 2.

### Genetic manipulation of R. capsulatus.

R. capsulatus and E. coli cultures were grown overnight for at least 24 h. R. capsulatus and E. coli S17-1 were allowed to conjugate for at least 24 h to transfer the desired plasmids into R. capsulatus. R. capsulatus strains containing the plasmids were selected using PYE with 20 μg/mL kanamycin, followed by colony PCR. Plasmid-containing cells were grown in liquid PYE with no antibiotics for at least 24 h. Cultures were then centrifuged, diluted, and plated on PYE agar + 10% sucrose or nutrient agar (NA) + 10% sucrose. Colonies were patch-plated in parallel onto PYE agar and PYE agar with antibiotics. Colonies that grew on PYE agar but not on PYE agar with antibiotics were identified as potential deletion or fusion strains. PCR was performed to confirm the deletion of the desired gene.

### Generation of plasmids.

Plasmids pTE1630-pTE1632 were generated by amplification of the up- and downstream genomic regions of targeted genes from Pd1222 genomic DNA with oligonucleotides 1 to 12 and subsequent Gibson assembly (54) of matching PCR products as indicated in Table 3 with EcoRI-linearized, dephosphorylated pK18mobsacB. Plasmids pTE1633 and pTE1634 were generated by amplification of inserts from Pd1222 gDNA with oligonucleotides 13 to 16 and subsequent ligation of adequately restricted PCR products into equally restricted and dephosphorylated pTE714. Plasmids pTE1637-1639 were generated by inverse amplification of pTE1634 with oligonucleotides 17 to 21, followed by removal of the template strand with DpnI, phosphorylation and ligation (KLD reaction). Plasmid pTE5000 was generated by inverse amplification of pIND4 with oligonucleotides 22 to 23, amplification of Pden_1365 with oligonucleotides 24 to 25 from Pd1222 genomic DNA and subsequent Gibson assembly of the resulting fragments. Plasmid pTE5007 was generated in two steps. First, the insert fragment was amplified from Pd1222 gDNA with oligonucleotides 26 to 27 and inserted into SapI-restricted pTB146 by Gibson assembly. A missing codon for a glycine-residue in the future Ulp1 recognition site was then inserted into the resulting construct by inverse amplification with oligonucleotides 28 to 29 and followed by a KLD reaction. Plasmids pMJ009, pYN011, pYN019, and pPK005 were generated by amplification of up- and downstream genomic regions of targeted genes from SB1003 genomic DNA and subsequent Gibson assembly of matching products according to Table 3 with BamHI- and XbaI-linearized, dephosphorylated pK18mobsacB. All oligonucleotides used in this study and their purpose are listed in Table 3.

**TABLE 3:** Oligonucleotides used in this study

#	Name	Sequence^*a*^	Cloning method/purpose	Construction of plasmid	Restriction site/modification
1	1350-1-f	TCGAGCTCGGTACCCGGGGATCCTCTAGAGGGATCGGCGGGATGTTGT	Gibson assembly	pTE1630	
2	1350-1-r	CTGCGGGCAGGCGGGGCTGGGCGAGATCCCCAGG	Gibson assembly	pTE1630	
3	1350-2-f	GGGATCTCGCCCAGCCCCGCCTGCCCGCAGCGC	Gibson assembly	pTE1630	
4	1350-2-r	CGGCCAGTGCCAAGCTTGCATGCCTGCAGGATCGCGTTCAGCCGCAGCAGGACCG	Gibson assembly	pTE1630	
5	1365-1-f	GAATTCGAGCTCGGTACCCGGGGATCCTCTAGAGCGGCGGGCAGGGTCTCGATCA	Gibson assembly	pTE1631	
6	1365-1-r	GCGGGCCGAGCAATCCGGGACGCCCAGCTCGGCCGCCA	Gibson assembly	pTE1631	
7	1365-2-f	GGCGGCCGAGCTGGGCGTCCCGGATTGCTCGGCCCGCG	Gibson assembly	pTE1631	
8	1365-2-r	GACGGCCAGTGCCAAGCTTGCATGCCTGCAGGCGCGGGCGACCCGGCCCC	Gibson assembly	pTE1631	
9	4472-1-f	TCGAGCTCGGTACCCGGGGATCCTCTAGAGTGCGCGACATCGTGCCGG	Gibson assembly	pTE1632	
10	4472-1-r	GCAGTTCGGCCGGGGTGAAACGCCCAGCCGGTC	Gibson assembly	pTE1632	
11	4472-2-f	CGGCTGGGCGTTTCACCCCGGCCGAACTGCCATCAGCGTT	Gibson assembly	pTE1632	
12	4472-2-r	ACGGCCAGTGCCAAGCTTGCATGCCTGCAGGCCATCGGCGGCGGCCAGGG	Gibson assembly	pTE1632	
13	EcoRI_Pden1364-f	GATATGAATTCCTGCCCGATTTCATCACCG	restriction/ligation	pTE1633	EcoRI
14	XbaI_STOP_Pden1363-r	cgTTCTAGATCAGTAGCGGGACGACACCTC	restriction/ligation	pTE1633	XbaI
15	EcoRI_STOP_Pden1365-r	GATATGAATTCTCAGCGCAGCACGCGCAGG	restriction/ligation	pTE1634	EcoRI
16	XbaI_STOP_Pden1364-r	CGTCTAGATCAGGCGCGCAGGACGACGGG	restriction/ligation	pTE1634	XbaI
17	1365_5UTR_binding1_mut-f	ATGCGTGGATTCTGCGGATGCACGATGACAATACT	KLD	pTE1637	
18	1365_5UTR_binding1_mut-r	CGCACTCCATGGCCCGAGGCGAGAAACTGATCATC	KLD	pTE1637	
19	1365_5UTR_binding2_mut-r	GCACTCTCGTGCATCCGCAGAATCTGTAAGGTTTG	KLD	pTE1638	
20	binding2_mut2-f	GCTGTGTGGCCGCACCCATTGCTTTCAAAGCAATA	KLD	pTE1638/39	
21	binding12_mut2-r	GCACTCTCGTGCATCCGCAGAATCCACGCATCGCA	KLD	pTE1639	
22	pIND4-f	GCTTAATTAGCTGAGCTTGGAC	Gibson assembly	pTE5000	
23	FLAG_pIND4-r	ATCGTCATCATCTTTGTAATCAATATCATGGTCTTTGTAGTCACCGTCGTGATCCTTATAGTCCATGGTTAATTTCTCCTCTTTAATTCTAG	Gibson assembly	pTE5000	
24	FLAG_linker_1365-f	GATTACAAAGATGATGACGATAAACTTCATCCTAGGGCAGGGAGTGCGGCCGGCAGCG	Gibson assembly	pTE5000	
25	pIND4_1365-r	AAGCTCAGCTAATTAAGCCTAACCCTCCTCGAAGCTGAAGATC	Gibson assembly	pTE5000	
26	Pden1365-f_NOSTRT	CACAGAGAACAGATTGGTGCCCGAGGCGAGAAACTGATCATC	Gibson assembly	pTE5007	
27	pTB146_1365-r(2)	ACGGAGCTCTGCTCTTCTCTAACCCTCCTCGAAGCTGAAGATCG	Gibson assembly	pTE5007	
28	pLT1-f	GGTGCCCGAGGCGAGAAACTG	KLD	pTE5007	
29	pLT1-r	ACCAATCTGTTCTCTGTGAGCC	KLD	pTE5007	
30	D16020UpF1	CTTGCATGCCTGCAGGTCGACTCTAGAGCCACGGCGATGTAAAGCGACAAAAG	Gibson assembly	pMJ009	
31	D16020UpR1	GGTGCGCAGCCCCATCTCGCCGATTTCGTAAAGGTCTTTCTC	Gibson assembly	pMJ009	
32	D16020DnF1	ATCGGCGAGATGGGGCTGCGCACCTTTGAAGATG	Gibson assembly	pMJ009	
33	D16020DnR1	GAATTCGAGCTCGGTACCCGGGGATCCGGGCGGATTCCCACAGGAACG	Gibson assembly	pMJ009	
34	D16495UpF1	CTTGCATGCCTGCAGGTCGACTCTAGACGGCACGGCTGGTCAAGGTCTG	Gibson assembly	pYN011	
35	D16495UpR1	CGTGTCGCCCAGGACCGCCTTGATCATCTGCTG	Gibson assembly	pYN011	
36	D16495DnF1	ATCAAGGCGGTCCTGGGCGACACGTTCAAGGAGATG	Gibson assembly	pYN011	
37	D16495DnR1	GAATTCGAGCTCGGTACCCGGGGATCCACGGTTGGCTGCACTGGATCGAAG	Gibson assembly	pYN011	
38	D04520UpF1	CTTGCATGCCTGCAGGTCGACTCTAGAGCATGGACCGGATGGTGCTTTCG	Gibson assembly	pYN019	
39	D04520UpR3	TTCCATGTCGTCGCGCAGTTTCACACCGGCATAAAG	Gibson assembly	pYN019	
40	D04520DnF2	GTGAAACTGCGCGACGACATGGAACTGGGCAATCCG	Gibson assembly	pYN019	
41	D04520DnR2	GAATTCGAGCTCGGTACCCGGGGATCCCTGTCGGATCAAGGGCAACATCTC	Gibson assembly	pYN019	
42	D16525UpF1	CTTGCATGCCTGCAGGTCGACTCTAGACGCACCAGTTCGACCTGAATTTCATAGC	Gibson assembly	pPK005	
43	D16525UpR1	CCACAGATAGCTCCGCACCCCCATGAATGCCTTTG	Gibson assembly	pPK005	
44	D16525DnF1	ATGGGGGTGCGGAGCTATCTGTGGATCGCCCGACAG	Gibson assembly	pPK005	
45	D16525DnR1	GAATTCGAGCTCGGTACCCGGGGATCCAGCGCGATGACCCTTGACGATG	Gibson assembly	pPK005	
46	bs_wt-f	TCTCGCCTCGGGCCATGTGACAAACCTTACAGATTCTGCGGATGCACGATGACAATACTGACTGCCGCACCCATTGCTTT	BLITz		5′-biotinylation
47	bs_wt-r	AAAGCAATGGGTGCGGCAGTCAGTATTGTCATCGTGCATCCGCAGAATCTGTAAGGTTTGTCACATGGCCCGAGGCGAGA	BLITz		
48	bs_mut12-f	TCTCGCCTCGGGCCATGGAGTGCGATGCGTGGATTCTGCGGATGCACGAGAGTGCGCTGTGTGGCCGCACCCATTGCTTT	BLITz		5′-biotinylation
49	bs_mut12-r	AAAGCAATGGGTGCGGCCACACAGCGCACTCTCGTGCATCCGCAGAATCCACGCATCGCACTCCATGGCCCGAGGCGAGA	BLITz		

a Recognition sites for restriction enzymes are underlined in the given sequences.

### Global transcriptome analysis.

Samples for transcriptome analysis were taken in triplicates from succinate- and acetate-growing cultures of P. denitrificans Pd1222 and the Pd1222 Δ*ramB* deletion at OD_600_ 0.8. For sample collection, 2 mL of culture were transferred into sterile 2-mL Eppendorf tubes and pelleted by centrifugation at 10,000 × *g* and 4°C for 10 min. Supernatant was discarded and samples were snap-frozen at −80°C. Storage of samples occurred at −80°C until further usage. The Qiagen miRNeasy Kit was used for total RNA isolation starting from a pellet of 2-mL cultured cells. The pellet was resuspended in 1 mL QiAzol lysis reagent and homogenized in a FastPrep sample preparation system using Lysing Matrix B containing 0.1 mm silica beads (MP biomedicals) and the following settings: 4 × 6,500 rpm for 20 s, 15-s break. The supernatant was transferred into a new tube and RNA isolation was performed according to the manufacturer’s instruction, including the optional on column DNase I digestion. Depletion of rRNA was performed and cDNA libraries were prepared with the Illumina Stranded Total RNA Prep Kit (Ligation with Ribo-Zero Plus; Illumina, USA). Library quality was assessed with the fragment analyzer employing the Agilent HS NGS Fragment kit (Agilent, USA). Quantification of libraries was done by qPCR and the KAPA library quantification Kit (Roche, Switzerland) in a qTOWER3 G Thermal Cycler (Analytic Jena, Germany). Libraries were paired-end sequenced on an Illumina MiSeq using the MiSeq reagent kit V3 featuring 150-bp read length. Sequencing data are available from ArrayExpress (reference number: E-MTAB-12482). The differential gene expression analysis was done as described elsewhere (55) using the RNA-seq pipeline Curare 0.3.1 (https://github.com/pblumenkamp/Curare). Briefly, reads were preprocessed with Trim Galore 0.6.7 (56) and Cutadapt 3.5 (57) with a quality and length threshold setting of 20 (phred score and base pairs, respectively). Preprocessed reads were aligned to the Pden1222 genome (chromosome 1: NC_008686.1, chromosome 2: NC_008687.1, plasmid: NC_008688.1) using Bowtie2 2.4.4 in “very-sensitive” mode and with “–mm” option (58).The resulting SAM/BAM files were processed with SAMtools 1.13 (59). Read-to-feature assignment was done using featureCounts (60) of the subread 2.0.1 package (61). Normalized read counts were then calculated and differential gene expression determined with DESeq2 1.32.0 (Tables S1 to 4) (62). For verification and analysis of the results, GenExVis 0.4.1 (https://github.com/pblumenkamp/GenExVis) was used.

### Synthesis of CoA esters.

Synthesis of CoA esters was performed as described (63) unless specified otherwise. β-Methylmalyl-CoA was synthesized from propionyl-CoA and a 12-fold molar excess of glyoxylate (in buffer: 100 mM MOPS/KOH pH 7.5, 5 mM MgCl_2_) using heterologously expressed and purified Rhodobacter sphaeroides Mcl-1 (accession number ACI22682) as catalyst. The resulting esters were purified via preparative HPLC-MS with a 1260 Infinity II LC System (Agilent, USA) in combination with a 6130 Single Quadrupole LC/MS (Agilent, USA). Lyophilized CoA esters were stored at −80°C and dissolved in ddH_2_O before use. The concentrations of the respective CoA esters were determined photometrically using a Carry 60 UV-Vis spectrophotometer (Agilent, USA) and calculated from the absorbance at 260 nm using defined extinction coefficients (22,300 M^−1 ^cm^−1^ for α-β-unsaturated fatty acid CoA thioesters and 16,400 M^−1 ^cm^−1^ for saturated ones). The amount of free sulfhydryl groups (indicating free CoA) present in the samples was quantified by treatment with Ellman’s reagent (DNTB) in EDTA-HEPES/KOH buffer (15 mM EDTA, 200 mM HEPES-KOH pH 7.8) and subsequent measurement of the absorbance at 412 nm (extinction coefficient: 14,000 M^−1 ^cm^−1^).

### Heterologous production and purification of proteins.

*6xhis-sumo-ramB* was heterologously expressed from plasmid pTE5007, respectively, in E. coli BL21 AI (Novagen, Germany) grown in terrific broth (TB; 24 g/L yeast extract, 20 g/L tryptone, 0.017 M KH_2_PO_4_, 0.072 M K_2_HPO_4_, 10% [wt/vol] glycerol) in the presence of ampicillin. Gene expression was induced by addition of 0.25% arabinose and 0.5 mM IPTG when the cultures had reached an OD_600_ of 1.0 to 2.0. *6xhis-ulp1* was expressed from plasmid pTB145 (64) in E. coli Rosetta (DE3)pLysS (Novagen, Germany) grown in TB supplemented with ampicillin and gentamicin. Gene expression was induced by addition of 0.5 mM IPTG when cultures had reached an OD_600_ of 1.0 to 2.0. Overproduction of all proteins occurred at 25°C overnight and was verified by SDS-PAGE analysis of culture samples taken before and after induction. The cultures were harvested by centrifugation at 8,000 × *g* and 10°C for 15 min. For lysis, the cells were resuspended in buffer A (50 mM Tris, 500 mM NaCl, pH 7.8) supplemented with 0.5 mM DTT, DNase I, and 5 mM MgCl_2_ and treated by four cycles of sonication (50%, 1s pulse, 60 s) using a Sonopuls GM200 sonicator (BANDELIN, Germany). Cell lysates were cleared by ultracentrifugation at 100,000 × *g* and 4°C for 45 min followed by filtration through a Filtropur S 0.45 μm filter (Sarstedt, Germany). His-tagged proteins in cell lysate were loaded on pre-equilibrated Ni-NTA agarose beads in Protino drop down columns (Macherey-Nagel, Germany), washed with buffer A and eluted with increasing concentrations of buffer B (50 mM Tris, 500 mM NaCl, 500 mM imidazole, pH 7.8). Buffer exchange was performed with Cytiva PD-10 Desalting columns (Cytiva, USA) and elution in desalting buffer (50 mM Tris, 350 mM NaCl, 10% glycerol, pH 7.8). His-SUMO tag was cleaved off by treatment of the fusion proteins with His-Ulp1 protease (1 U per 2 μg of target protein) in desalting buffer, followed by removal of His-SUMO protein with pre-equilibrated Ni-NTA agarose beads. Successful purification of proteins, as well as proteolytic cleavage of tags was verified by SDS-PAGE. Protein concentrations were determined using a NanoDrop 2000 (Thermo Fisher Scientific, USA). After successful purification, RamB_Pd_ was routinely analyzed via UV-vis spectroscopy to verify the presence of the previously described [4Fe4S] cluster as displayed by a characteristic peak at 410 nm in UV-vis spectroscopy (39).

### Mass photometry.

The molecular weights of protein-protein complexes were determined in phosphate-buffered saline (PBS; pH 7.4) on 1.5 H, 24 × 60 mm microscope coverslips (Carl Roth, Germany) and Culture Well Reusable Gaskets (GRACE BIO-LABS, USA) using a Refeyn One mass photometer (Refeyn Ltd., UK) with the AcquireMP software (Refeyn Ltd., UK). A previously measured standard was used at a concentration of 20 nM as a reference. The processing and analysis of mass photometry images was performed using DiscoverMP (Refeyn Ltd., UK).

### Biolayer interferometry.

The analysis of protein-DNA interactions by biolayer interferometry (BLI) was performed using Sartorius Octet SAX2 biosensors in binding buffer (desalting buffer supplemented with 0.01% Tween 20 and 10 μM bovine serum albumin (BSA)) on a BLItz platform (FortéBio, USA). Biotinylated dsDNA at 40 μM concentration was generated by annealing oligonucleotides 46 and 47 for wt DNA with intact binding sites and oligonucleotides 48 and 49 (Table 3) for DNA with mutated binding sites in Phusion high GC buffer (Thermo Fisher Scientific, USA) dsDNA stocks were diluted 20-fold in binding buffer to reach a working concentration of 2 μM. To immobilize biotinylated dsDNA fragments on the biosensor tips, 4 μL of the 2 μM stock were applied to the sample drop holder and the biosensor tip was placed in it. DNA loading was followed for 120 s until equilibrium was reached. Afterwards, the biosensor tip was transferred back into binding buffer to wash off excess DNA fragments for 30 s. The association of protein with the DNA-loaded biosensor tip was followed as described for DNA loading. After each sample loading step, the sample drop holder was cleaned with 0.5 M NaOH, followed by two washes with ddH_2_O. After the association step, the biosensor tip was transferred into binding buffer again and protein dissociation was followed for 120 s. A new biosensor tip was used for each measurement.

### Data availability.

Sequencing data are available from ArrayExpress (reference number: E-MTAB-12482).
