# Supplementary material for: Functional Degeneracy in Paracoccus denitrificans Pd1222 Is Coordinated via RamB, Which Links Expression of the Glyoxylate Cycle to Activity of the Ethylmalonyl-CoA Pathway
Source: Appl Environ Microbiol. 2023 Jun 15;89(7):e00238-23. doi: 10.1128/aem.00238-23 (PMC10370305; doi:10.1128/aem.00238-23)
Supplement: Supplemental file 1 — Supplemental material. Download aem.00238-23-s0001.docx, DOCX file, 7.4 MB [file aem.00238-23-s0001.docx]

**Supplementary Information***:*


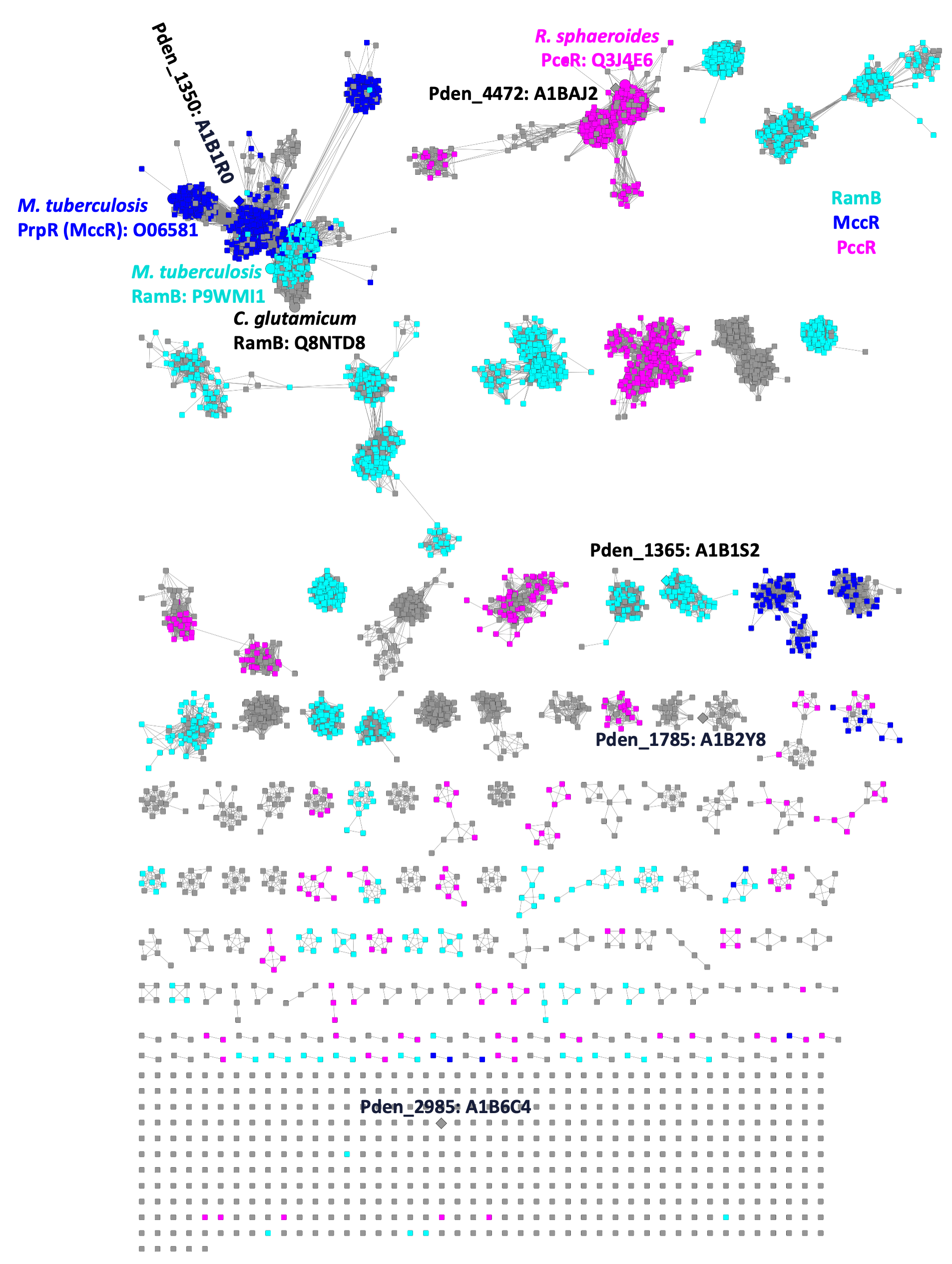


Supplementary Figure 1: Sequence similarity network (SSN) of the ScfR family (PF09856). The network was generated by EFI-EST using UniProt version 2022_03 and InterPro version 90. Nodes represent proteins from the ScfR family. Lines (edges) connect nodes whose proteins are related by alignment scores better than e^-151^. EFI-GNT was used to identify conserved genome neighborhoods that encoded the proteins from the SSN in the figure. Nodes are colored according to the neighborhoods in which the respective proteins are encoded. Proteins identified as RamB (teal) are encoded in neighborhoods that included aceA and/or aceB. Proteins identified as MccR (blue) are encoded in neighborhoods that include at least two of the following: prpB, prpC, or prpD. Proteins identified as PccR (pink) are encoded in neighborhoods that included pccA, pccB, or mcm. Proteins represented by gray nodes are not encoded in neighborhoods that included the aforementioned genes. Circular nodes are marked with the Swiss-Prot annotation and UniProt identifiers of the respective proteins. Diamond nodes represent proteins of Paracoccus denitrificans Pd1222 and are labeled with the respective Pden_ gene tags and UniProt identifiers in black.


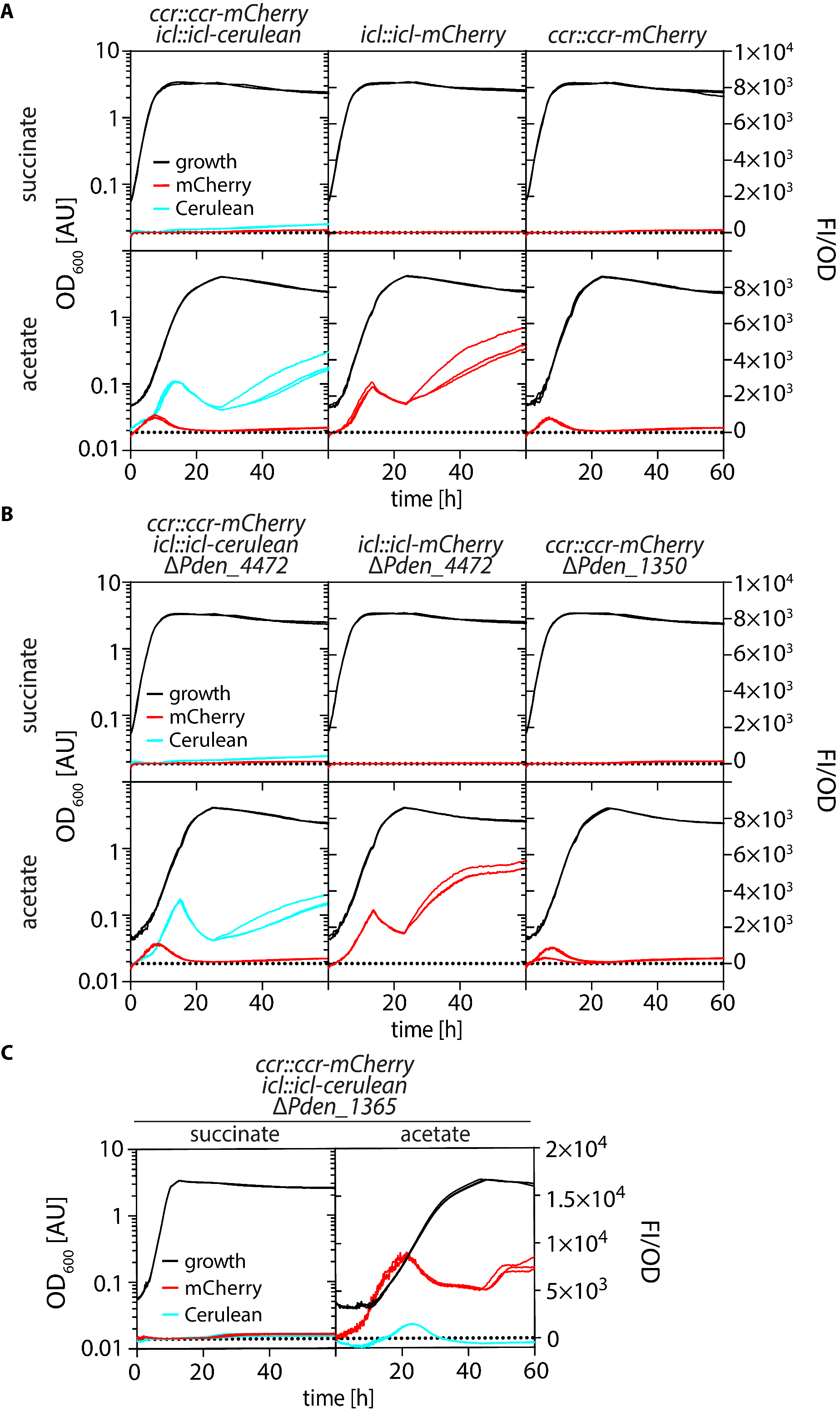


Supplementary Figure 2: Deletion of scfR homologues in different Pd1222 reporter backgrounds. Growth is given as OD_600_ on the left y-axis. Fluorescence normalized to OD_600_ is depicted as on the right y-axis. Replicates are shown as individual curves. Genotypes indicating the respective fluorescent fusions present in the different strains are given above the panels. Growth and fluorescence of unaffected scfR mutants (B) and their parental strains (A) on succinate (upper rows) and acetate (lower rows). (C) Growth and fluorescence of ccr::ccr-mCherry icl::icl-cerulean ΔPden_1365 (TJE-KK20) on succinate and acetate.


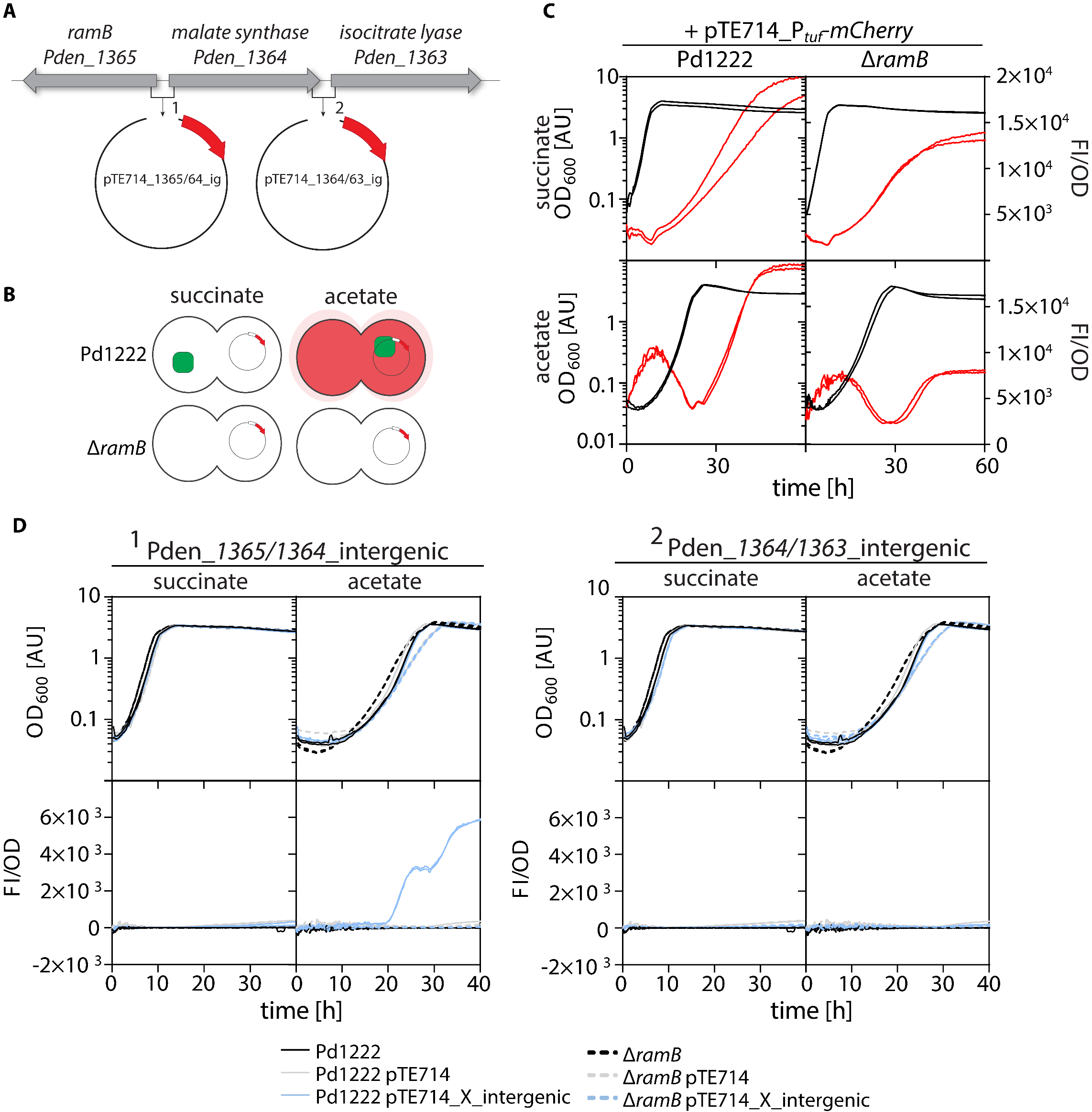


Supplementary Figure 3: The genes of the GC are organized in an operon. (A) Simplified depiction of the GC genes in reverse complement. icl (Pden_1363) and ms (Pden_1364) are separated by an intergenic region of 120 bp. ms and ramB (Pden_1365) are separated by 134 bp. The individual intergenic regions including 50 bp of their respective flanking open reading frames (ORFs) were integrated into plasmid pTE714, yielding plasmids pTE714_1365/64_ig (pTE1634) and pTE714_1364/63_ig (pTE1633), respectively. In the resulting plasmids, the intergenic regions are followed by an otherwise promoterless mCherry gene. (B) Predicted outcome of the reporter assay. In wild-type Pd1222, RamB is present but inactive during growth on succinate. When shifted to acetate, the regulator is activated and induces gene expression from the promoter region of its target gene. If the reporter plasmid present in the cell carries this region, expression of mCherry will be induced, resulting in fluorescence of the cell. In the ΔramB mutant, gene expression from the reporter plasmids cannot be induced due to the lack of regulator. (C and D) Growth and fluorescence of Pd1222 and Pd1222 ΔramB carrying different variants of pTE714. Growth is depicted as OD_600_ on the left y-axis. Fluorescence corrected by OD_600_ is given as on the right y-axis. (C) Positive controls. The Pd1222 strains carry a derivative of pTE714 (pTE770) with mCherry under the control of the constitutive P_tuf_ promoter. Here, an mCherry signal was detected in all strains under all conditions, verifying the functionality of the mCherry gene as a reliable reporter. (D) Fluorescence reporter assay with Pd1222 strains carrying different versions (1 or 2) of the reporter constructs indicted above the panels.


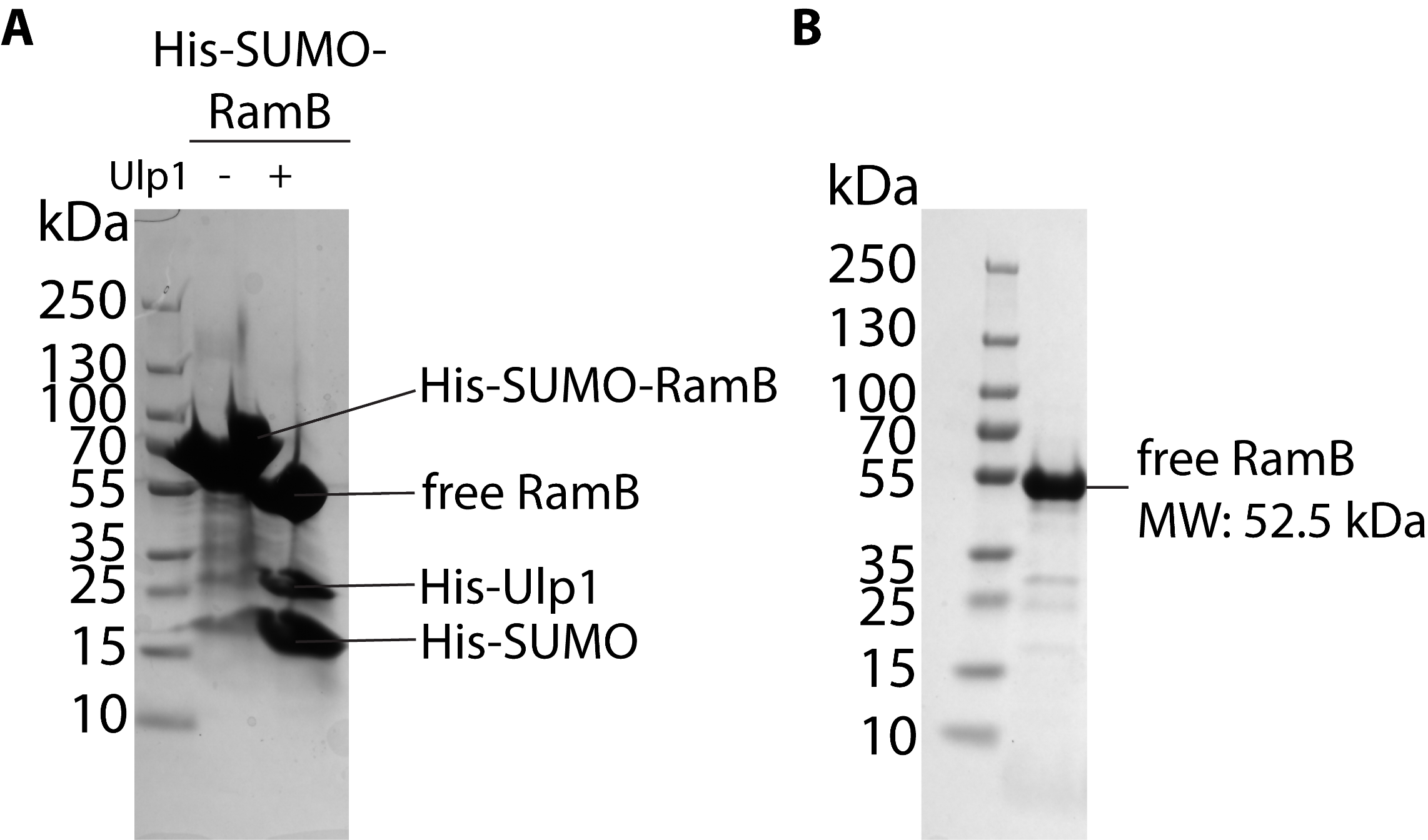


Supplementary Figure 4: Purification of free Pden_1365 (RamB_Pd_). Molecular weights: His-SUMO-Pden_1365 (65.2 kDa), free Pden_1365 (52.5 kDa), His-Ulp1 (27.0 kDa), His-SUMO-tag (12.7 kDa). (A) SDS gel with His-SUMO-Pden_1365 after purification via affinity chromatography from BL21 AI cell extracts before (-) and after proteolytic cleavage with His-Ulp1 (+). (B) SDS-gel with free Pden_1365 after removal of His-Ulp1 and the cleaved His-SUMO-tag.


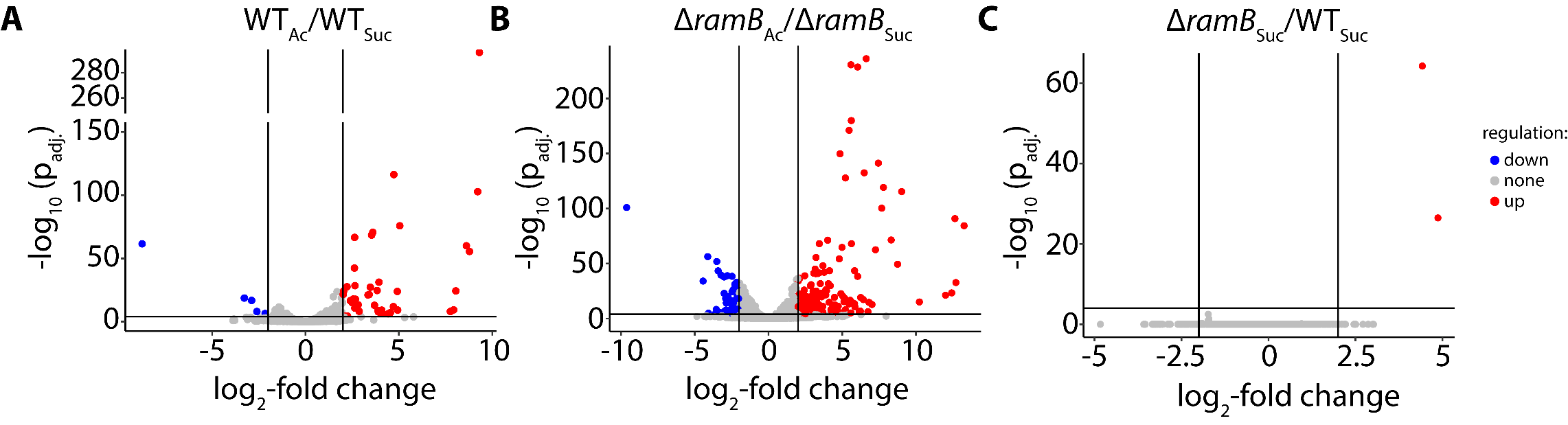


Supplementary Figure 5: Volcano plots showing the regulation of genes in Pd1222 WT and ΔramB under different conditions. Dots represent individual genes. Thresholds above which genes were considered significantly up- or downregulated are indicated by black lines. (A) Regulation of genes in Pd1222 WT during growth on acetate vs. succinate. (B) Regulation of genes in Pd1222 ΔramB during growth on acetate vs. succinate. (C) Regulation of genes on succinate in Pd1222 ΔramB vs. WT.


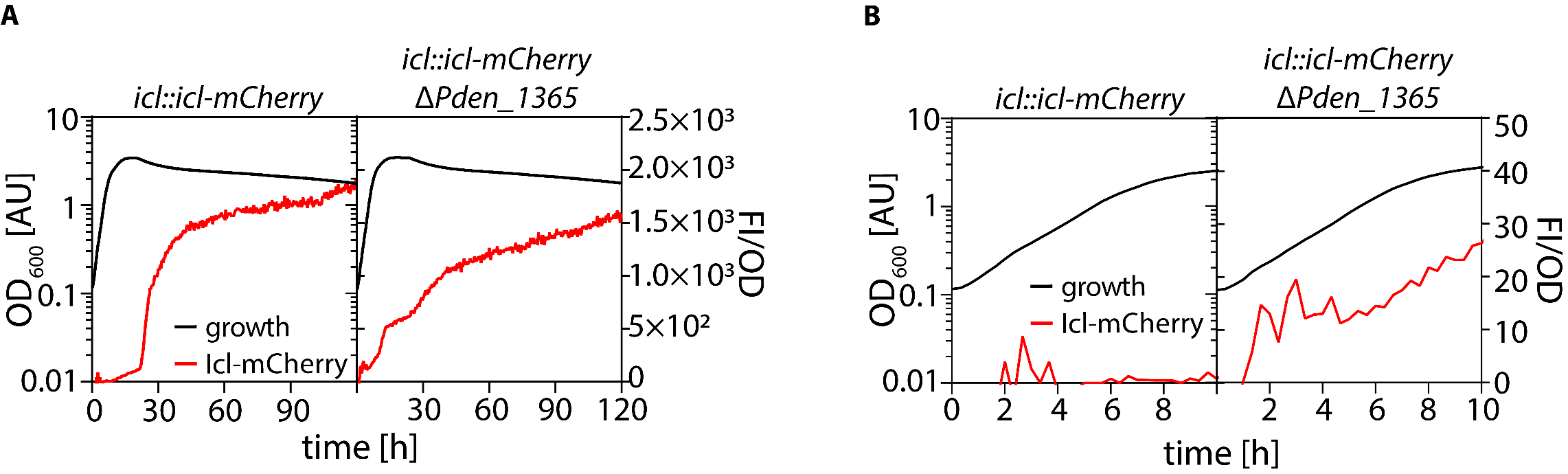


Supplementary Figure 6: Growth and fluorescence of Pd1222 icl::icl-mCherry (TJE-KK3) and Pd1222 icl::icl-mCherry ΔPden_1365 (TJE-KK16) on succinate. Growth is depicted as OD_600_ on the left y-axes, fluorescence normalized to OD_600_ is given as on the right y-axes. Shown are data from the same experiment as in Figure 2 with an adjusted right y-axis (A) and adjusted right y- and x-axes (B) for a better representation of the basal mCherry fluorescence observed during exponential growth of the strains on succinate.


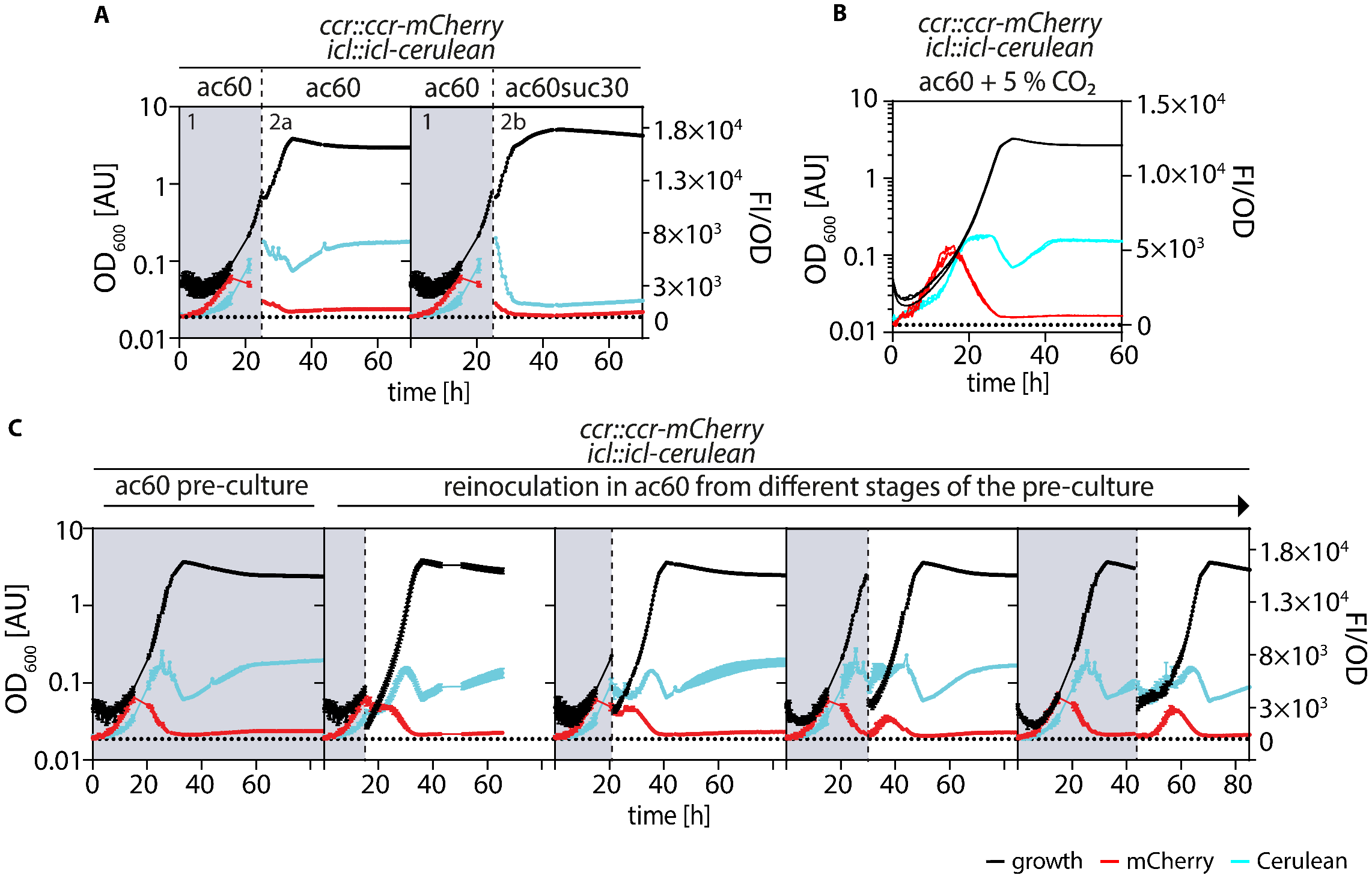


Supplementary Figure 7: Influence of additional carbon sources and pre-culture growth state on ccr and icl expression. Growth and fluorescence of Pd1222 ccr::ccr-mCherry icl::icl-cerulean (TJE-KK12) on different carbon sources. Growth is given as OD_600_ on the left y-axes, fluorescence normalized to OD_600_ is depicted as on the right y-axes. (A) Cells were grown to mid-exponential phase on 60 mM acetate (1; gray background), then washed and resuspended in fresh 60 mM acetate medium (2a; white background) or 60 mM acetate medium supplemented with 30 mM succinate (2b; white background). (B) Cells were grown on 60 mM acetate with a constant supply with 5 % CO_2_ in the atmosphere. (C) Cells were grown to different growth stages in 60 mM acetate medium (gray background) and used to re-inoculate fresh cultures to a starting OD_600_ of 0.05 (white background).


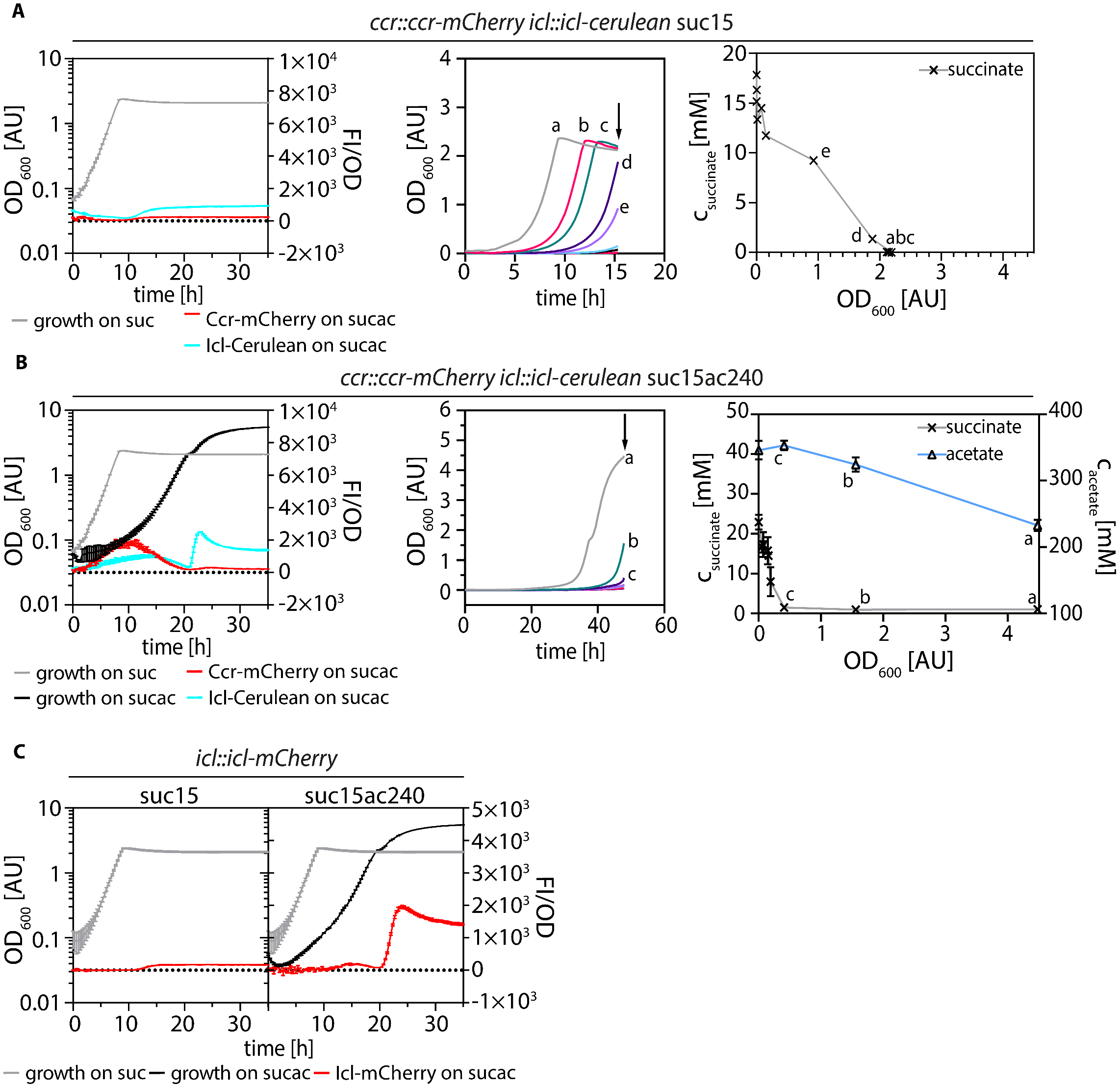


Supplementary Figure 8: Expression of the GC occurs only upon metabolization of acetate. Growth is depicted as OD_600_ on the left y-axes, fluorescence normalized to OD_600_ is depicted as on the right y-axes. (A) Left panel: Growth and fluorescence of Pd1222 ccr::ccr-mCherry icl::icl-cerulean (TJE-KK12) on 15 mM succinate. Middle panel: Growth of TJE-KK12 on 15 mM succinate to different optical densities. At the timepoints indicated by arrows, growth was stopped and the cultures were passed through a MultiScreen_HTS_-GV 0.22 µM filter plate (Millipore, Germany) to remove cells from the spent medium. Spent medium was then analyzed for the concentrations of succinate and acetate by LC-MS as shown in the right panel. Right panel: Concentrations of succinate and acetate in spent medium of the cultures shown in the middle panel after removal of cells. The measured carbon source concentrations are plotted versus the corresponding OD_600_ of the cultures prior to filtration. (B) Left panel: Growth and fluorescence of TJE-KK12 on 15 mM succinate plus 240 mM acetate (black). To demonstrate the maximal reachable OD_600_ on 15 mM succinate only, the growth of TJE-KK12 on 15 mM succinate is shown in addition (gray). Middle and right panels: as described in (A). (C) Growth and fluorescence of Pd1222 icl::icl-mCherry (TJE-KK3) on 15 mM succinate (left panel, gray curve) and 15 mM succinate plus 240 mM acetate (right panel, black curve). To demonstrate the maximal reachable OD_600_ on 15 mM succinate only, the growth of TJE-KK12 on 15 mM succinate is additionally shown in the right panel (gray).


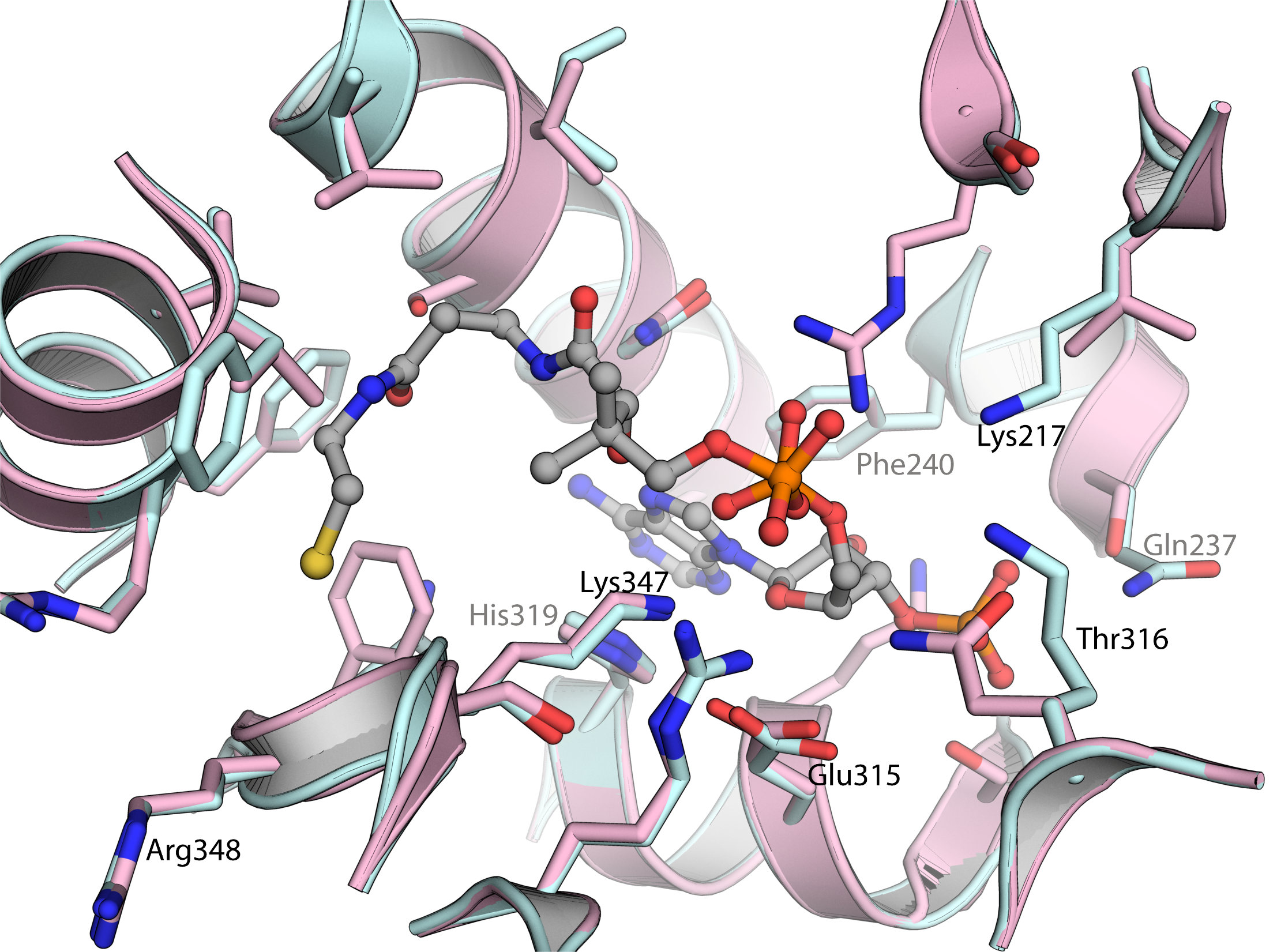


Supplementary Figure 9: Pd1222 RamB_Pd_ contains a CoA binding cavity. Superposition of a Pd1222 RamB_Pd_ homology model (pink) and M. tuberculosis PrpR (teal, PDB 6CYY). Depicted is the CoA binding site with bound CoA (grey) as observed in PrpR (39). Amino acid residues described to be engaged in the coordination of CoA in M. tuberculosis PrpR (39) are labeled. These binding site residues are well-conserved in RamB_Pd_ with only few exceptions. Lys217 is replaced by Leu211, Gln237 is replaced by Ser231, and Thr316 is replaced by Gln310 in RamB_Pd_.Supplementary Table 1: Top regulated genes WT_Ac_/WT_Suc_. Listed are all genes regulated above a threshold of log_2_-fold 2.0 in the Pd1222 wild type on acetate compared to Pd1222 on succinate.

| gene_id | Gene | padj | neg. log10(padjust) | pvalue | log2FoldChange | regulation | CDS/product description | old locus tag | new locus tag |
| --- | --- | --- | --- | --- | --- | --- | --- | --- | --- |
| cds-WP_086000156.1 | *aceA* | 9.73E-297 | 296 | 2.76E-300 | 9.3 | up | aceA | Pden_1363 | PDEN_RS06725 |
| cds-WP_011747683.1 | *aceB* | 1.61E-103 | 103 | 1.37E-106 | 9.2 | up | aceB | Pden_1364 | PDEN_RS06730 |
| cds-WP_011747670.1 | *prpD* | 3.09E-56 | 56 | 8.77E-59 | 8.8 | up | prpD | Pden_1348 | PDEN_RS06660 |
| cds-WP_011747669.1 | *prpC* | 9.99E-61 | 60 | 2.55E-63 | 8.6 | up | prpC | Pden_1347 | PDEN_RS06655 |
| cds-WP_041530157.1 | *prpB* | 4.97E-25 | 24 | 2.40E-27 | 8.0 | up | prpB | Pden_1346 | PDEN_RS06650 |
| cds-PDEN_RS26015 |  | 5.16E-10 | 9 | 6.59E-12 | 7.9 | up | transcriptional repressor | - | PDEN_RS26015 |
| cds-WP_011747671.1 |  | 9.57E-09 | 8 | 1.47E-10 | 7.8 | up | thioesterase | Pden_1349 | PDEN_RS06665 |
| cds-WP_011750441.1 |  | 1.50E-76 | 76 | 1.71E-79 | 5.0 | up | DUF485 domain-containing protein CDS | Pden_4212 | PDEN_RS20985 |
| cds-WP_011751202.1 |  | 7.18E-10 | 9 | 9.37E-12 | 4.9 | up | alphakteoacid-dh subunit beta | Pden_4984 | PDEN_RS24785 |
| cds-WP_011750439.1 |  | 1.34E-24 | 24 | 7.21E-27 | 4.9 | up | response regulator | Pden_4210 | PDEN_RS20975 |
| cds-WP_011750440.1 |  | 4.74E-117 | 116 | 2.69E-120 | 4.7 | up | cation acetate symporter | Pden_4211 | PDEN_RS20980 |
| cds-WP_011746654.1 |  | 1.39E-12 | 12 | 1.46E-14 | 4.7 | up | tricarboxylate transporter | Pden_0307 | PDEN_RS01490 |
| WP_011751203.1 |  | 7.25E-08 | 7 | 1.30E-09 | 4.5 | up | PDH E1 suA | Pden_4985 | PDEN_RS24790 |
| cds-WP_011751200.1 |  | 6.28E-07 | 6 | 1.30E-08 | 4.4 | up | Glc1-DH | Pden_4982 | PDEN_RS24775 |
| cds-WP_011747582.1 |  | 2.12E-07 | 7 | 4.04E-09 | 4.1 | up | metal ABC transporter substrate-binding protein CDS | Pden_1259 | PDEN_RS06215 |
| cds-WP_011747581.1 |  | 8.73E-10 | 9 | 1.16E-11 | 4.1 | up | manganese/iron ABC transporter ATP-binding protein CDS | Pden_1258 | PDEN_RS06210 |
| cds-WP_011746655.1 |  | 6.40E-32 | 31 | 2.18E-34 | 3.9 | up | tripartite tricarboxylate transporter substrate binding protein CDS | Pden_0308 | PDEN_RS01495 |
| cds-WP_011747580.1 |  | 1.81E-08 | 8 | 2.93E-10 | 3.9 | up | metal ABC transporter permease CDS | Pden_1257 | PDEN_RS06205 |
| cds-WP_049792306.1 |  | 1.53E-09 | 9 | 2.13E-11 | 3.9 | up | hypothetical protein | Pden_1351 | PDEN_RS06675 |
| cds-WP_011746653.1 |  | 2.40E-25 | 25 | 1.09E-27 | 3.8 | up | tripartite tricarboxylate transporter permease CDS | Pden_0306 | PDEN_RS01485 |
| cds-WP_011747579.1 |  | 1.24E-13 | 13 | 1.23E-15 | 3.7 | up | metal ABC transporter permease CDS | Pden_1256 | PDEN_RS06200 |
| cds-WP_011750442.1 |  | 2.35E-71 | 71 | 3.34E-74 | 3.6 | up | acs (acetate CoA ligase) | Pden_4213 | PDEN_RS20990 |
| cds-WP_011750774.1 |  | 3.47E-69 | 68 | 5.90E-72 | 3.5 | up | acs (acetate CoA ligase) | Pden_4550 | PDEN_RS22665 |
| cds-WP_041530430.1 |  | 6.20E-28 | 27 | 2.64E-30 | 3.5 | up | PALP-dep enzyme | Pden_3920 | PDEN_RS19505 |
| cds-WP_011750154.1 |  | 3.85E-22 | 21 | 2.40E-24 | 3.4 | up | aminotransferase class V-fold PLP-dependent enzyme CDS | Pden_3921 | PDEN_RS19510 |
| cds-WP_011750438.1 |  | 8.16E-22 | 21 | 5.32E-24 | 3.3 | up | bifunctional enoyl-CoA hydratase/phosphate acetyltransferase CDS | Pden_4209 | PDEN_RS20970 |
| cds-WP_011751214.1 |  | 7.01E-09 | 8 | 1.06E-10 | 2.9 | up | acs (acetate CoA ligase) | Pden_4996 | PDEN_RS24845 |
| cds-WP_011750151.1 |  | 5.19E-14 | 13 | 5.01E-16 | 2.8 | up | ornithine cyclodeaminase family protein CDS | Pden_3918 | PDEN_RS19495 |
| cds-WP_011750150.1 |  | 1.87E-18 | 18 | 1.48E-20 | 2.7 | up | purine permease CDS | Pden_3917 | PDEN_RS19490 |
| cds-WP_011750630.1 |  | 3.59E-29 | 28 | 1.32E-31 | 2.6 | up | hypothetical protein | Pden_4404 | PDEN_RS21930 |
| cds-WP_011750629.1 |  | 1.85E-11 | 11 | 2.04E-13 | 2.6 | up | tripartite tricarboxylate transporter TctB family protein CDS | Pden_4403 | PDEN_RS21925 |
| cds-WP_011750527.1 |  | 2.31E-67 | 67 | 4.58E-70 | 2.6 | up | acnA (aconitat hydratase) | Pden_4301 | PDEN_RS21425 |
| cds-WP_041530425.1 |  | 4.35E-43 | 42 | 1.36E-45 | 2.6 | up | ecm | Pden_3875 | PDEN_RS19280 |
| cds-WP_011750152.1 |  | 1.38E-18 | 18 | 1.06E-20 | 2.6 | up | DSD1 family PLP-dependent enzyme CDS | Pden_3919 | PDEN_RS19500 |
| cds-WP_011750790.1 |  | 1.39E-15 | 15 | 1.26E-17 | 2.5 | up | EamA family transporter CDS | Pden_4566 | PDEN_RS22745 |
| cds-WP_011750791.1 |  | 2.52E-17 | 17 | 2.15E-19 | 2.4 | up | bifunctional aconitate hydratase 2/2-methylisocitrate dehydratase CDS | Pden_4567 | PDEN_RS22750 |
| cds-WP_011750599.1 |  | 5.04E-05 | 4 | 1.32E-06 | 2.3 | up | TonB-dependent siderophore receptor CDS | Pden_4373 | PDEN_RS21785 |
| cds-WP_011750106.1 | *ccrA* | 2.52E-28 | 28 | 1.00E-30 | 2.2 | up | Crotonyl-CoA carboxylase/reductase | Pden_3873 | PDEN_RS19270 |
| cds-WP_011750149.1 |  | 5.04E-05 | 4 | 1.33E-06 | 2.1 | up | 8-oxoguanine deaminase CDS | Pden_3916 | PDEN_RS19485 |
| cds-WP_011749235.1 |  | 6.77E-25 | 24 | 3.46E-27 | 2.0 | up | NADP-dependent isocitrate dehydrogenase CDS | Pden_2961 | PDEN_RS14750 |
| cds-WP_041529775.1 |  | 2.72E-22 | 22 | 1.62E-24 | 2.0 | up | L-malyl-CoA/beta-methylmalyl-CoA lyase CDS | Pden_0799 | PDEN_RS03970 |
| cds-WP_011747496.1 |  | 3.95E-07 | 6 | 7.97E-09 | -2.2 | down | TonB-dependent receptor CDS | Pden_1171 | PDEN_RS05800 |
| cds-WP_128492943.1 |  | 1.05E-08 | 8 | 1.65E-10 | -2.6 | down | ammonium transporter CDS | Pden_2032 | PDEN_RS10105 |
| cds-WP_011750899.1 |  | 1.83E-17 | 17 | 1.50E-19 | -2.9 | down | rpmC CDS ribosomal protein | Pden_0767 | PDEN_RS03810 |
| cds-WP_164901616.1 |  | 2.35E-19 | 19 | 1.73E-21 | -3.3 | down | TonB-dependent siderophore receptor CDS | Pden_3029 | PDEN_RS15110 |
| cds-WP_011750985.1 |  | 2.88E-62 | 62 | 6.54E-65 | -8.7 | down | dicarboxylate/amino acid:cation symporter CDS | Pden_4765 | PDEN_RS23720 |

Supplementary Table 2: Top regulated genes KO_Ac_/KO_Suc_. Listed are all genes regulated above a threshold of log_2_-fold 2.0 in Pd1222 ΔramB on acetate compared to Pd1222 ΔramB on succinate.

| gene_id | Gene | padj | neg. log10(padjust) | pvalue | log2FoldChange | regulation | CDS/product description | Old locus tag | New locus tag |
| --- | --- | --- | --- | --- | --- | --- | --- | --- | --- |
| cds-WP_011747670.1 | *prpD* | 4.30972E-85 | 84 | 1.40169E-87 | 13.3 | up | MmgE/PrpD family protein CDS | Pden_1348 |  |
| cds-WP_041530157.1 | *prpB* | 1.85554E-33 | 33 | 1.97141E-35 | 12.7 | up | prpB CDS | Pden_1346 |  |
| cds-WP_011747669.1 | *prpC* | 1.37001E-91 | 91 | 4.15876E-94 | 12.6 | up | prpC | Pden_1347 |  |
| cds-PDEN_RS26015 | *-* | 4.46679E-24 | 23 | 8.23238E-26 | 12.4 | up | transcriptional repressor prp-operon | - | PDEN_RS26015 |
| cds-WP_011747671.1 | *-* | 5.70502E-22 | 21 | 1.28648E-23 | 12.0 | up | acyl-CoA thioesterase CDS | Pden_1349 | PDEN_RS06665 |
| cds-WP_011750346.1 | *-* | 7.43866E-16 | 15 | 2.91934E-17 | 10.2 | up | VOC family protein CDS | Pden_4115 | PDEN_RS20505 |
| cds-PDEN_RS20500 | *-* | 4.0324E-116 | 115 | 9.6177E-119 | 9.0 | up | acyl-CoA/acyl-ACP dehydrogenase CDS | Pden_4113 | PDEN_RS20500 |
| cds-WP_049792306.1 | *-* | 4.03678E-50 | 49 | 2.27572E-52 | 8.8 | up | hypothetical protein CDS | Pden_1351 | PDEN_RS06675 |
| cds-WP_011750347.1 | *-* | 3.70507E-72 | 71 | 1.28537E-74 | 8.3 | up | hypothetical protein CDS | Pden_4116 | PDEN_RS20510 |
| cds-WP_011750351.1 | *-* | 6.5218E-120 | 119 | 1.4141E-122 | 7.8 | up | TRAP transporter large permease subunit CDS | Pden_4120 | PDEN_RS20530 |
| cds-WP_198140555.1 | *-* | 4.8799E-101 | 100 | 1.3755E-103 | 7.7 | up | TRAP transporter small permease subunit CDS | Pden_4121 | PDEN_RS20535 |
| cds-WP_011750353.1 | *-* | 5.4393E-142 | 141 | 8.2557E-145 | 7.4 | up | TRAP transporter substrate-binding protein CDS | Pden_4122 | PDEN_RS20540 |
| cds-WP_011750439.1 | *-* | 2.9281E-63 | 63 | 1.33326E-65 | 7.3 | up | response regulator CDS | Pden_4210 | PDEN_RS20975 |
| cds-WP_011750635.1 | *-* | 1.61018E-13 | 13 | 8.55364E-15 | 7.0 | up | hydantoinase B/oxoprolinase family protein CDS | Pden_4409 | PDEN_RS21955 |
| cds-WP_011750345.1 | *-* | 1.94225E-15 | 15 | 8.0436E-17 | 6.8 | up | maoC family dehydratase | Pden_4111 | PDEN_RS20490 |
| cds-WP_011751204.1 | *-* | 5.17068E-07 | 6 | 6.9062E-08 | 6.7 | up | NAD(+)/NADH kinase CDS | Pden_4986 | PDEN_RS24795 |
| cds-WP_011750440.1 | *-* | 6.8547E-237 | 236 | 1.4863E-240 | 6.6 | up | cation acetate symporter CDS | Pden_4211 | PDEN_RS20980 |
| cds-WP_011750441.1 | *-* | 3.4732E-133 | 132 | 6.0246E-136 | 6.5 | up | DUF485 domain-containing protein CDS | Pden_4212 | PDEN_RS20985 |
| cds-WP_011746370.1 | *-* | 5.13834E-18 | 17 | 1.57092E-19 | 6.4 | up | methanol/ethanol family PQQ-dependent dehydrogenase CDS | Pden_0020 | PDEN_RS00105 |
| cds-WP_011750437.1 | *-* | 6.91563E-20 | 19 | 1.84437E-21 | 6.3 | up | acetate/propionate family kinase CDS | Pden_4208 | PDEN_RS20965 |
| cds-WP_011748741.1 | *-* | 1.67472E-07 | 7 | 2.04074E-08 | 6.2 | up | filamentous hemagglutinin N-terminal domain-containing protein CDS | Pden_2461 | PDEN_RS12235 |
| cds-WP_011746372.1 | *-* | 6.01885E-07 | 6 | 8.14346E-08 | 6.1 | up | substrate-binding domain-containing protein CDS | Pden_0022 | PDEN_RS00115 |
| cds-WP_011750350.1 | *-* | 4.48668E-39 | 38 | 3.98859E-41 | 6.0 | up | fumarat hydratase | Pden_4119 | PDEN_RS20525 |
| cds-WP_011750106.1 | *ccrA* | 2.5411E-229 | 229 | 1.6529E-232 | 6.0 | up | ccrA | Pden_3873 | PDEN_RS18165 |
| cds-WP_011751203.1 | *-* | 2.28313E-11 | 11 | 1.63767E-12 | 5.9 | up | thiamine pyrophosphate-dependent dehydrogenase E1 component subunit alpha CDS | Pden_4985 | PDEN_RS24790 |
| cds-WP_011750195.1 | *-* | 3.23154E-44 | 43 | 2.10204E-46 | 5.8 | up | coA transferase | Pden_4112 | PDEN_RS20495 |
| cds-WP_011750344.1 | *-* | 4.55829E-14 | 13 | 2.18426E-15 | 5.8 | up | coA ester lyase | Pden_4110 | PDEN_RS20485 |
| cds-WP_011751200.1 | *-* | 8.77269E-15 | 14 | 3.95646E-16 | 5.7 | up | glucose 1-dehydrogenase CDS | Pden_4982 | PDEN_RS24775 |
| cds-WP_011750438.1 | *-* | 8.75216E-69 | 68 | 3.60562E-71 | 5.6 | up | bifunctional enoyl-CoA hydratase/phosphate acetyltransferase CDS | Pden_4209 | PDEN_RS20970 |
| cds-WP_011750442.1 | *acs* | 1.1766E-180 | 180 | 1.0205E-183 | 5.6 | up | acetyl-CoA ligase | Pden_4213 | PDEN_RS20990 |
| cds-WP_041530425.1 | *-* | 2.2765E-231 | 231 | 9.8719E-235 | 5.6 | up | protein meaA CDS/mcm | Pden_3875 | PDEN_RS19280 |
| cds-WP_011748740.1 | *-* | 6.56687E-06 | 5 | 1.04369E-06 | 5.6 | up | ShlB/FhaC/HecB family hemolysin secretion/activation protein CDS | Pden_2460 | PDEN_RS12230 |
| cds-WP_011746374.1 | *-* | 2.45305E-17 | 17 | 7.92506E-19 | 5.5 | up | SRPBCC family protein CDS | Pden_0024 | PDEN_RS00125 |
| cds-WP_011750774.1 | *acs* | 9.1521E-172 | 171 | 9.9221E-175 | 5.5 | up | acetate coA ligase CDS | Pden_4550 | PDEN_RS22665 |
| cds-WP_041530124.1 | *hydA* | 4.9607E-14 | 13 | 2.39861E-15 | 5.5 | up | dihidropyrimidinase | Pden_1112 | PDEN_RS05510 |
| cds-WP_011749115.1 | *-* | 1.7495E-128 | 128 | 3.4141E-131 | 5.2 | up | acyl-CoA/acyl-ACP dehydrogenase CDS | Pden_2840 | PDEN_RS14140 |
| cds-WP_011747441.1 | *-* | 9.94657E-09 | 8 | 1.00285E-09 | 5.2 | up | Zn dependent hydrolase | Pden_1113 | PDEN_RS05515 |
| cds-WP_081465060.1 | *-* | 4.66576E-19 | 18 | 1.3455E-20 | 5.2 | up | ompA family protein | Pden_2459 | PDEN_RS25615 |
| cds-WP_011751202.1 | *-* | 4.32877E-16 | 15 | 1.58621E-17 | 5.1 | up | coA ester lyase | Pden_4110 | PDEN_RS20485 |
| cds-WP_011748724.1 | *tssC* | 6.37613E-21 | 20 | 1.54841E-22 | 5.0 | up | tssC CDS (part of type VI secretion system) | Pden_2444 | PDEN_RS12150 |
| cds-WP_011750349.1 | *-* | 1.99488E-65 | 65 | 8.65081E-68 | 5.0 | up | acyl-CoA/Acyl-ACP-DH | Pden_4113 | PDEN_RS20500 |
| cds-PDEN_RS26020 | *-* | 5.51764E-06 | 5 | 8.63777E-07 | 5.0 | up | ribonucleoside triphosphate reductase CDS | Pden_1345 | PDEN_RS26020 |
| cds-WP_011749103.1 | *fdxH* | 2.50497E-23 | 23 | 4.9969E-25 | 4.9 | up | fdxH CDS (formate dh) | Pden_2828 | PDEN_RS14080 |
| cds-WP_041529775.1 | *-* | 1.7325E-150 | 150 | 2.2538E-153 | 4.9 | up | L-malyl-CoA/beta-methylmalyl-CoA lyase CDS = mcl-1 | Pden_0799 | PDEN_RS03970 |
| cds-WP_011750348.1 | *-* | 5.14592E-55 | 54 | 2.67784E-57 | 4.8 | up | class I SAM-dependent methyltransferase CDS | Pden_4117 | PDEN_RS20515 |
| cds-WP_011751201.1 | *-* | 6.59275E-12 | 11 | 4.3742E-13 | 4.7 | up | acetoin dehydrogenase dihydrolipoyllysine-residue acetyltransferase subunit CDS | Pden_4983 | PDEN_RS24780 |
| cds-WP_011748644.1 | *pqqC* | 6.64845E-07 | 6 | 9.09621E-08 | 4.7 | up | pyrroloquinoline-quinone synthase PqqC | Pden_2361 | PDEN_RS11725 |
| cds-WP_011749179.1 | *-* | 1.03188E-08 | 8 | 1.04261E-09 | 4.7 | up | enoyl-CoA hydratase/isomerase family protein CDS | Pden_2905 | PDEN_RS14470 |
| cds-WP_011750428.1 | *-* | 1.94702E-12 | 12 | 1.20739E-13 | 4.6 | up | hypothetical protein CDS | Pden_4199 | PDEN_RS20920 |
| cds-WP_049792275.1 | *-* | 4.54269E-13 | 12 | 2.59048E-14 | 4.5 | up | ABC transporter substrate-binding protein CDS | Pden_0026 | PDEN_RS00135 |
| cds-WP_011748651.1 | *-* | 4.6819E-20 | 19 | 1.23849E-21 | 4.5 | up | DUF779 protein | Pden_2368 | PDEN_RS11765 |
| cds-WP_011746378.1 | *-* | 2.3877E-13 | 13 | 1.30464E-14 | 4.4 | up | hypothetical protein CDS | Pden_0028 | PDEN_RS00145 |
| cds-WP_011748177.1 | *-* | 3.11737E-07 | 7 | 4.00148E-08 | 4.4 | up | DUF2147 domain-containing protein CDS | Pden_1885 | PDEN_RS09315 |
| cds-WP_011748723.1 | *tssB* | 1.23541E-08 | 8 | 1.26702E-09 | 4.4 | up | type VI secretion system contractile sheath small subunit | Pden_2443 | PDEN_RS12145 |
| cds-WP_011748725.1 | *-* | 7.58753E-13 | 12 | 4.54067E-14 | 4.4 | up | type VI secretion system tube protein Hcp CDS | Pden_2445 | PDEN_RS12155 |
| cds-WP_011750780.1 | *-* | 1.16669E-05 | 5 | 1.94027E-06 | 4.4 | up | 3'-5' exonuclease CDS | Pden_4556 | PDEN_RS22695 |
| cds-WP_049792433.1 | *-* | 9.07072E-12 | 11 | 6.13631E-13 | 4.4 | up | hydantoinase/oxoprolinase family protein CDS | Pden_4408 | PDEN_RS21950 |
| cds-WP_024843519.1 | *pqqA* | 1.79816E-07 | 7 | 2.20676E-08 | 4.3 | up | pqqA CDS (pyroloquinone synthesis) | - | PDEN_RS11735 |
| cds-WP_011748645.1 | *pqqB* | 7.90747E-09 | 8 | 7.86975E-10 | 4.2 | up | pyrroloquinoline quinone biosynthesis protein PqqB | Pden_2362 | PDEN_RS11730 |
| cds-WP_011748641.1 | *-* | 2.18742E-05 | 5 | 3.81803E-06 | 4.2 | up | quinoprotein dehydrogenase-associated SoxYZ-likecarrier CDS | Pden_2358 | PDEN_RS11710 |
| cds-WP_041530319.1 | *-* | 1.33483E-30 | 30 | 1.59184E-32 | 4.2 | up | AMP-binding protein CDS | Pden_2909 | PDEN_RS14490 |
| cds-WP_041530599.1 | *-* | 3.16588E-44 | 43 | 1.99069E-46 | 4.1 | up | MaoC family dehydratase CDS | Pden_3661 | PDEN_RS18165 |
| cds-WP_011746377.1 | *-* | 1.5629E-15 | 15 | 6.40476E-17 | 4.1 | up | YVTN family beta-propeller repeat protein CDS | Pden_0027 | PDEN_RS00140 |
| cds-WP_011746655.1 | *-* | 1.8323E-32 | 32 | 2.06591E-34 | 4.1 | up | tripartite tricarboxylate transporter substrate binding protein CDS | Pden_0308 | PDEN_RS01495 |
| cds-WP_198140528.1 | *fdnG* | 6.11179E-72 | 71 | 2.25283E-74 | 4.0 | up | fdnG CDS (formate dh) | Pden_2829 | PDEN_RS14085 |
| cds-WP_011748650.1 | *adhP* | 5.07158E-26 | 25 | 8.35732E-28 | 3.9 | up | alcohol-DH | Pden_2367 | PDEN_RS11760 |
| cds-WP_011748649.1 | *-* | 6.78671E-27 | 26 | 1.00064E-28 | 3.9 | up | aldehyde dehydrogenase family protein CDS | Pden_2366 | PDEN_RS11755 |
| cds-WP_011749181.1 | *-* | 2.93138E-22 | 22 | 6.48311E-24 | 3.9 | up | acetyl-CoA C-acyltransferase CDS | Pden_2907 | PDEN_RS14480 |
| cds-WP_011749960.1 | *-* | 9.56337E-43 | 42 | 6.63547E-45 | 3.8 | up | LuxR family transcriptional regulator CDS | Pden_3724 | PDEN_RS18480 |
| cds-WP_011746908.1 | *mcl-2* | 1.01917E-48 | 48 | 5.9665E-51 | 3.7 | up | CoA ester lyase CDS | Pden_0563 | PDEN_RS02805 |
| cds-WP_011751217.1 | *-* | 1.01323E-06 | 6 | 1.43021E-07 | 3.7 | up | branched-chain amino acid ABC transporter permease CDS | Pden_4999 | PDEN_RS24860 |
| cds-WP_155984334.1 | *-* | 7.53288E-12 | 11 | 5.0633E-13 | 3.7 | up | hypothetical protein CDS | - | PDEN_RS26985 |
| cds-WP_011750691.1 | *-* | 1.80466E-20 | 20 | 4.61729E-22 | 3.6 | up | hypothetical protein CDS | Pden_4466 | PDEN_RS22255 |
| cds-WP_011750942.1 | *-* | 8.5441E-24 | 23 | 1.61174E-25 | 3.6 | up | nitrate reductase cytochrome c-type subunit CDS | Pden_4722 | PDEN_RS23505 |
| cds-WP_011747066.1 | *-* | 1.75818E-32 | 32 | 1.94422E-34 | 3.5 | up | patatin-like phospholipase family protein CDS | Pden_0721 | PDEN_RS03585 |
| cds-WP_011746869.1 | *-* | 1.01414E-10 | 10 | 7.78413E-12 | 3.5 | up | purine-nucleoside phosphorylase CDS | Pden_0524 | PDEN_RS02610 |
| cds-WP_011750427.1 | *-* | 7.30621E-06 | 5 | 1.16912E-06 | 3.5 | up | RiPP maturation radical SAM C-methyltransferase CDS | Pden_4198 | PDEN_RS20915 |
| cds-WP_011746654.1 | *-* | 9.80012E-09 | 8 | 9.85962E-10 | 3.5 | up | tripartite tricarboxylate transporter TctB family protein CDS | Pden_0307 | PDEN_RS01490 |
| cds-WP_011748720.1 | *tssH* | 1.43033E-10 | 10 | 1.11958E-11 | 3.5 | up | tssH CDS (type IV secretion system ATPase) | Pden_2440 | PDEN_RS12130 |
| cds-WP_011746911.1 | *-* | 7.95539E-69 | 68 | 3.10488E-71 | 3.4 | up | MaoC family dehydratase CDS | Pden_0566 | PDEN_RS02820 |
| cds-WP_011749579.1 | *-* | 4.64028E-11 | 10 | 3.43091E-12 | 3.4 | up | hypothetical protein CDS | Pden_3318 | PDEN_RS16475 |
| cds-WP_011748365.1 | *-* | 3.04568E-42 | 42 | 2.17926E-44 | 3.4 | up | hypothetical protein CDS | Pden_2078 | PDEN_RS10325 |
| cds-WP_011749102.1 | *-* | 2.5488E-13 | 13 | 1.40924E-14 | 3.4 | up | formate dehydrogenase subunit gamma CDS | Pden_2827 | PDEN_RS14075 |
| cds-WP_011746653.1 | *-* | 1.13916E-21 | 21 | 2.64288E-23 | 3.4 | up | tripartite tricarboxylate transporter permease CDS | Pden_0306 | PDEN_RS01485 |
| cds-WP_011749182.1 | *-* | 6.91107E-11 | 10 | 5.15483E-12 | 3.4 | up | SDR family NAD(P)-dependent oxidoreductase CDS | Pden_2908 | PDEN_RS14485 |
| cds-WP_011750943.1 | *-* | 1.13687E-41 | 41 | 8.6276E-44 | 3.4 | up | cytochrome c3 family protein CDS | Pden_4723 | PDEN_RS23510 |
| cds-WP_011750777.1 | *-* | 1.54807E-11 | 11 | 1.09761E-12 | 3.3 | up | hypothetical protein CDS | Pden_4553 | PDEN_RS22680 |
| cds-WP_011749876.1 | *-* | 1.72693E-06 | 6 | 2.52001E-07 | 3.3 | up | glutathione S-transferase CDS | Pden_3640 | PDEN_RS18050 |
| cds-WP_011749180.1 | *-* | 4.30404E-16 | 15 | 1.56782E-17 | 3.3 | up | acyl-CoA dehydrogenase family protein CDS | Pden_2906 | PDEN_RS14475 |
| cds-WP_011751197.1 | *-* | 1.07524E-09 | 9 | 9.62865E-11 | 3.3 | up | sigma-54-dependent Fis family transcriptional regulator CDS | Pden_4979 | PDEN_RS24760 |
| cds-WP_011746754.1 | *-* | 4.65539E-07 | 6 | 6.1574E-08 | 3.3 | up | glutathione S-transferase family protein CDS | Pden_0407 | PDEN_RS02020 |
| cds-WP_011748943.1 | *-* | 9.8708E-26 | 25 | 1.66939E-27 | 3.2 | up | acetyl-CoA C-acyltransferase family protein CDS | Pden_2663 | PDEN_RS13255 |
| cds-WP_011748761.1 | *-* | 3.21781E-06 | 5 | 4.87694E-07 | 3.2 | up | VWA domain-containing protein CDS | Pden_2481 | PDEN_RS12330 |
| cds-WP_011750941.1 | *napA* | 3.08196E-56 | 56 | 1.53697E-58 | 3.2 | up | nitrate reductase | Pden_4721 | PDEN_RS23500 |
| cds-WP_011750776.1 | *-* | 1.51575E-41 | 41 | 1.18315E-43 | 3.2 | up | cation acetate symporter CDS | Pden_4552 | PDEN_RS22675 |
| cds-WP_011750775.1 | *-* | 7.95356E-16 | 15 | 3.13865E-17 | 3.2 | up | DUF4212 domain-containing protein CDS | Pden_4551 | PDEN_RS22670 |
| cds-WP_011749819.1 | *-* | 1.9589E-05 | 5 | 3.38517E-06 | 3.2 | up | helix-turn-helix transcriptional regulator CDS | Pden_3582 | PDEN_RS17750 |
| cds-WP_011749580.1 | *-* | 9.75971E-11 | 10 | 7.46512E-12 | 3.2 | up | hypothetical protein CDS | Pden_3319 | PDEN_RS16480 |
| cds-WP_011747141.1 | *-* | 1.29467E-05 | 5 | 2.16995E-06 | 3.2 | up | ABC transporter permease CDS | Pden_0801 | PDEN_RS03985 |
| cds-WP_011750768.1 | *-* | 9.64117E-07 | 6 | 1.3567E-07 | 3.2 | up | TRAP transporter small permease subunit CDS | Pden_4544 | PDEN_RS22635 |
| cds-WP_041530739.1 | *-* | 1.80032E-05 | 5 | 3.09551E-06 | 3.2 | up | branched-chain amino acid ABC transporter permease CDS | Pden_5000 | PDEN_RS24865 |
| cds-WP_011746909.1 | *-* | 1.03606E-45 | 45 | 6.29002E-48 | 3.2 | up | NnrU family protein CDS | Pden_0564 | PDEN_RS02810 |
| cds-WP_011749262.1 | *-* | 5.60611E-13 | 12 | 3.24551E-14 | 3.1 | up | response regulator transcription factor CDS | Pden_2990 | PDEN_RS14925 |
| cds-WP_011749695.1 | *-* | 7.58016E-09 | 8 | 7.52756E-10 | 3.1 | up | efflux RND transporter periplasmic adaptor subunit CDS | Pden_3447 | PDEN_RS17075 |
| cds-WP_011750423.1 | *-* | 1.23293E-17 | 17 | 3.90302E-19 | 3.1 | up | SARP family transcriptional regulator CDS | Pden_4194 | PDEN_RS20895 |
| cds-WP_011749875.1 | *-* | 2.94793E-08 | 8 | 3.21511E-09 | 3.1 | up | ATP-grasp domain-containing protein CDS | Pden_3639 | PDEN_RS18045 |
| cds-WP_011749887.1 | *aqpZ* | 1.05782E-11 | 11 | 7.40844E-13 | 3.1 | up | aqpZ CDS (aquaporin) | Pden_3651 | PDEN_RS18105 |
| cds-WP_011748964.1 | *-* | 5.80276E-42 | 41 | 4.27784E-44 | 3.1 | up | DUF2853 family protein CDS | Pden_2684 | PDEN_RS13360 |
| cds-WP_011749581.1 | *-* | 3.95201E-24 | 23 | 7.19794E-26 | 3.1 | up | VWA domain-containing protein CDS | Pden_3320 | PDEN_RS16485 |
| cds-WP_011746380.1 | *-* | 6.05177E-05 | 4 | 1.14947E-05 | 3.0 | up | ABC transporter permease CDS | Pden_0030 | PDEN_RS00155 |
| cds-WP_011749577.1 | *-* | 3.20521E-08 | 7 | 3.51656E-09 | 3.0 | up | hypothetical protein CDS | Pden_3316 | PDEN_RS16465 |
| cds-WP_074810587.1 | *-* | 1.11666E-25 | 25 | 1.91275E-27 | 3.0 | up | YdcH family protein CDS (methyltransferase) | Pden_2029 | PDEN_RS10090 |
| cds-WP_011749106.1 | *-* | 2.39457E-17 | 17 | 7.68422E-19 | 3.0 | up | hybrid-cluster NAD(P)-dependent oxidoreductase CDS | Pden_2831 | PDEN_RS14095 |
| cds-WP_011746935.1 | *-* | 4.84807E-15 | 14 | 2.08675E-16 | 3.0 | up | outer membrane protein transport protein CDS | Pden_0590 | PDEN_RS02940 |
| cds-WP_011750977.1 | *-* | 1.56669E-22 | 22 | 3.36301E-24 | 3.0 | up | 3-methyl-2-oxobutanoate dehydrogenase (2-methylpropanoyl-transferring) subunit alpha CDS | Pden_4757 | PDEN_RS23680 |
| cds-WP_011749391.1 | *-* | 1.08733E-14 | 14 | 4.97455E-16 | 3.0 | up | recombinase family protein CDS | Pden_3123 | PDEN_RS15550 |
| cds-WP_011750769.1 | *-* | 5.31956E-16 | 15 | 1.98388E-17 | 3.0 | up | TRAP transporter large permease subunit CDS | Pden_4545 | PDEN_RS22640 |
| cds-WP_011748715.1 | *-* | 4.62323E-05 | 4 | 8.55078E-06 | 3.0 | up | hypothetical protein CDS | Pden_2435 | PDEN_RS12105 |
| cds-WP_011750436.1 | *-* | 6.0014E-08 | 7 | 6.88365E-09 | 3.0 | up | polyhydroxyalkanoic acid synthase CDS | Pden_4207 | PDEN_RS20960 |
| cds-WP_011749263.1 | *-* | 1.51237E-07 | 7 | 1.83308E-08 | 2.9 | up | HAMP domain-containing protein CDS | Pden_2991 | PDEN_RS14930 |
| cds-WP_011749578.1 | *-* | 3.98472E-09 | 8 | 3.87067E-10 | 2.9 | up | hypothetical protein CDS | Pden_3317 | PDEN_RS16470 |
| cds-WP_011749835.1 | *-* | 5.12081E-10 | 9 | 4.38578E-11 | 2.9 | up | hypothetical protein CDS | Pden_3599 | PDEN_RS27125 |
| cds-WP_011749582.1 | *-* | 5.81499E-08 | 7 | 6.6194E-09 | 2.9 | up | hypothetical protein CDS | Pden_3321 | PDEN_RS16490 |
| cds-WP_011748551.1 | *mscL* | 1.59977E-31 | 31 | 1.8731E-33 | 2.9 | up | mscL CDS (mechanosensitive channel protein) | Pden_2265 | PDEN_RS11260 |
| cds-WP_011751071.1 | *-* | 3.42961E-13 | 12 | 1.93343E-14 | 2.9 | up | caspase family protein | Pden_4852 | PDEN_RS24150 |
| cds-WP_011746365.1 | *gfa* | 4.61173E-07 | 6 | 6.08965E-08 | 2.9 | up | gfa CDS (glutathion synthase) | Pden_0015 | PDEN_RS00080 |
| cds-WP_011749647.1 | *-* | 5.68427E-05 | 4 | 1.07843E-05 | 2.9 | up | twin-arginine translocation pathway signal CDS | Pden_3398 | PDEN_RS16825 |
| cds-WP_011747579.1 | *-* | 3.74546E-09 | 8 | 3.63014E-10 | 2.9 | up | metal ABC transporter permease CDS | Pden_1256 | PDEN_RS06200 |
| cds-WP_011746795.1 | *-* | 5.42676E-13 | 12 | 3.12992E-14 | 2.9 | up | hypothetical protein CDS | Pden_0448 | PDEN_RS02230 |
| cds-WP_011749944.1 | *-* | 1.00326E-09 | 9 | 8.89709E-11 | 2.9 | up | DUF1330 domain-containing protein CDS | Pden_3708 | PDEN_RS18410 |
| cds-WP_011749576.1 | *-* | 9.90065E-13 | 12 | 5.98933E-14 | 2.8 | up | hypothetical protein CDS | Pden_3315 | PDEN_RS27080 |
| cds-WP_011746910.1 | *-* | 1.93264E-30 | 30 | 2.38856E-32 | 2.8 | up | DUF1737 domain-containing protein CDS | Pden_0565 | PDEN_RS02815 |
| cds-WP_011748722.1 | *-* | 4.96211E-05 | 4 | 9.26361E-06 | 2.8 | up | type VI secretion system ImpA family N-terminal domain-containing protein CDS | Pden_2442 | PDEN_RS12140 |
| cds-WP_011751215.1 | *-* | 5.21718E-05 | 4 | 9.83029E-06 | 2.8 | up | ABC transporter ATP-binding protein CDS | Pden_4997 | PDEN_RS24850 |
| cds-WP_011749873.1 | *-* | 6.00185E-13 | 12 | 3.50064E-14 | 2.8 | up | methylcrotonoyl-CoA carboxylase CDS | Pden_3637 | PDEN_RS18035 |
| cds-WP_049792328.1 | *-* | 3.82534E-06 | 5 | 5.88896E-07 | 2.8 | up | sigma-54-dependent Fis family transcriptional regulator CDS | Pden_2365 | PDEN_RS11750 |
| cds-WP_011748735.1 | *tssI* | 3.57935E-09 | 8 | 3.4381E-10 | 2.8 | up | type VI secretion system tip protein VgrG | Pden_2455 | PDEN_RS12205 |
| cds-WP_011748395.1 | *-* | 3.31588E-07 | 6 | 4.27786E-08 | 2.8 | up | Crp/Fnr family transcriptional regulator CDS | Pden_2108 | PDEN_RS10470 |
| cds-WP_011750754.1 | *-* | 5.86663E-08 | 7 | 6.70363E-09 | 2.8 | up | sodium:solute symporter family protein CDS | Pden_4529 | PDEN_RS22575 |
| cds-WP_011746794.1 | *-* | 3.60495E-20 | 19 | 9.37976E-22 | 2.8 | up | hypothetical protein CDS | Pden_0447 | PDEN_RS02225 |
| cds-WP_011750770.1 | *dctP* | 1.08124E-18 | 18 | 3.16493E-20 | 2.8 | up | dctP CDS | Pden_4546 | PDEN_RS22645 |
| cds-WP_164901615.1 | *-* | 6.64984E-08 | 7 | 7.65626E-09 | 2.8 | up | hypothetical protein CDS | - | PDEN_RS27345 |
| cds-WP_011750560.1 | *-* | 6.2623E-13 | 12 | 3.66614E-14 | 2.7 | up | APC family permease CDS | Pden_4334 | PDEN_RS21580 |
| cds-WP_011750863.1 | *-* | 1.06349E-12 | 12 | 6.47964E-14 | 2.7 | up | tyrosinase family protein CDS | Pden_4643 | PDEN_RS23115 |
| cds-WP_011749264.1 | *-* | 3.38897E-08 | 7 | 3.74756E-09 | 2.7 | up | protein MoxZ CDS | Pden_2992 | PDEN_RS14935 |
| cds-WP_049792297.1 | *-* | 8.8447E-06 | 5 | 1.43832E-06 | 2.7 | up | DNA/RNA non-specific endonuclease CDS | Pden_0949 | PDEN_RS04715 |
| cds-WP_011750773.1 | *-* | 1.26297E-13 | 13 | 6.54488E-15 | 2.6 | up | iron-containing alcohol dehydrogenase CDS | Pden_4549 | PDEN_RS22660 |
| cds-WP_011749107.1 | *-* | 1.78847E-20 | 20 | 4.49832E-22 | 2.6 | up | aromatic ring-hydroxylating dioxygenase subunit alpha CDS | Pden_2832 | PDEN_RS14100 |
| cds-WP_011746953.1 | *-* | 2.64213E-19 | 19 | 7.3329E-21 | 2.6 | up | peptide ABC transporter substrate-binding protein CDS | Pden_0608 | PDEN_RS03025 |
| cds-WP_011750740.1 | *-* | 4.10865E-17 | 16 | 1.37193E-18 | 2.6 | up | VOC family protein CDS | Pden_4515 | PDEN_RS22505 |
| cds-WP_011750633.1 | *-* | 2.99821E-05 | 5 | 5.35674E-06 | 2.6 | up | allantoin permease CDS | Pden_4407 | PDEN_RS21945 |
| cds-WP_011746952.1 | *-* | 6.55491E-16 | 15 | 2.51565E-17 | 2.6 | up | ABC transporter permease CDS | Pden_0607 | PDEN_RS03020 |
| cds-WP_011750487.1 | *xdhB* | 1.1606E-13 | 13 | 5.98923E-15 | 2.6 | up | xanthine dehydrogenase molybdopterin binding subunit | Pden_4258 | PDEN_RS21210 |
| cds-WP_011750418.1 | *ugpA* | 4.02813E-19 | 18 | 1.13542E-20 | 2.6 | up | sn-glycerol-3-phosphate ABC transporter permeaseUgpA | Pden_4189 | PDEN_RS20870 |
| cds-WP_011750978.1 | *-* | 2.46167E-09 | 9 | 2.30582E-10 | 2.6 | up | alpha-ketoacid dehydrogenase subunit beta CDS | Pden_4758 | PDEN_RS23685 |
| cds-WP_011746738.1 | *-* | 4.50678E-05 | 4 | 8.2963E-06 | 2.6 | up | hypothetical protein CDS | Pden_0391 | PDEN_RS01930 |
| cds-WP_011746796.1 | *-* | 1.38646E-13 | 13 | 7.27501E-15 | 2.6 | up | DNA polymerase III subunit epsilon CDS | Pden_0449 | PDEN_RS25930 |
| cds-WP_011750417.1 | *ugpE* | 1.59096E-18 | 18 | 4.69147E-20 | 2.6 | up | sn-glycerol-3-phosphate ABC transporter permeaseUgpE | Pden_4188 | PDEN_RS20865 |
| cds-WP_011746954.1 | *-* | 8.56217E-15 | 14 | 3.84295E-16 | 2.6 | up | ABC transporter ATP-binding protein CDS | Pden_0609 | PDEN_RS03030 |
| cds-WP_128492982.1 | *-* | 5.46752E-08 | 7 | 6.20016E-09 | 2.6 | up | hypothetical protein CDS | - | PDEN_RS04720 |
| cds-WP_011747391.1 | *-* | 4.88201E-09 | 8 | 4.79521E-10 | 2.6 | up | flavin reductase family protein CDS | Pden_1062 | PDEN_RS05275 |
| cds-WP_011750940.1 | *-* | 4.0844E-11 | 10 | 2.99334E-12 | 2.6 | up | chaperone NapD CDS | Pden_4720 | PDEN_RS23495 |
| cds-WP_011750059.1 | *-* | 1.01888E-11 | 11 | 7.11358E-13 | 2.6 | up | CoA-binding protein CDS | Pden_3824 | PDEN_RS19025 |
| cds-WP_011746951.1 | *-* | 7.02985E-07 | 6 | 9.63327E-08 | 2.6 | up | ABC transporter permease CDS | Pden_0606 | PDEN_RS03015 |
| cds-WP_011746870.1 | *-* | 5.34176E-17 | 16 | 1.80684E-18 | 2.5 | up | ABC transporter permease CDS | Pden_0525 | PDEN_RS02615 |
| cds-WP_041529929.1 | *-* | 7.64945E-10 | 9 | 6.71732E-11 | 2.5 | up | hypothetical protein CDS | - | PDEN_RS10215 |
| cds-WP_011749574.1 | *-* | 9.43538E-11 | 10 | 7.18087E-12 | 2.5 | up | molybdopterin-dependent oxidoreductase CDS | Pden_3313 | PDEN_RS16450 |
| cds-WP_011747752.1 | *bioB* | 2.76344E-15 | 15 | 1.15643E-16 | 2.5 | up | bioB CDS (part of biotin synthesis/import cluster) | Pden_1433 | PDEN_RS07085 |
| cds-WP_198140556.1 | *-* | 5.14456E-05 | 4 | 9.67116E-06 | 2.5 | up | hydantoinase/oxoprolinase family protein CDS | Pden_4263 | PDEN_RS21235 |
| cds-WP_011751198.1 | *-* | 2.51754E-08 | 8 | 2.71296E-09 | 2.5 | up | 2,3-butanediol dehydrogenase CDS | Pden_4980 | PDEN_RS24765 |
| cds-WP_011746553.1 | *-* | 1.33024E-05 | 5 | 2.23821E-06 | 2.5 | up | SDR family oxidoreductase CDS | Pden_0206 | PDEN_RS00990 |
| cds-WP_011748717.1 | *-* | 2.12119E-05 | 5 | 3.69783E-06 | 2.5 | up | DUF4150 domain-containing protein CDS | Pden_2437 | PDEN_RS25610 |
| cds-WP_011748261.1 | *-* | 5.64885E-05 | 4 | 1.07008E-05 | 2.5 | up | recombinase family protein CDS | Pden_1972 | PDEN_RS26105 |
| cds-WP_011750702.1 | *-* | 7.23386E-10 | 9 | 6.28963E-11 | 2.5 | up | CBS domain-containing protein CDS | Pden_4477 | PDEN_RS22310 |
| cds-WP_011747298.1 | *-* | 1.31044E-39 | 39 | 1.10813E-41 | 2.5 | up | hypothetical protein CDS | Pden_0959 | PDEN_RS04770 |
| cds-WP_041530327.1 | *-* | 1.54435E-05 | 5 | 2.63002E-06 | 2.5 | up | hypothetical protein CDS | - | PDEN_RS15600 |
| cds-WP_011747675.1 | *-* | 1.78372E-07 | 7 | 2.18517E-08 | 2.4 | up | methionine synthase CDS | Pden_1355 | PDEN_RS06690 |
| cds-WP_011748716.1 | *-* | 6.6643E-12 | 11 | 4.43612E-13 | 2.4 | up | hypothetical protein CDS | Pden_2436 | PDEN_RS12110 |
| cds-WP_011748294.1 | *-* | 1.52114E-08 | 8 | 1.56996E-09 | 2.4 | up | acyl-CoA synthetase CDS | Pden_2005 | PDEN_RS09970 |
| cds-WP_011748341.1 | *-* | 2.10131E-07 | 7 | 2.60614E-08 | 2.4 | up | hypothetical protein CDS | Pden_2053 | PDEN_RS10205 |
| cds-WP_041530076.1 | *-* | 8.93522E-22 | 21 | 2.03425E-23 | 2.4 | up | ABC transporter permease CDS | Pden_0526 | PDEN_RS02620 |
| cds-WP_011748015.1 | *-* | 3.9757E-08 | 7 | 4.46534E-09 | 2.4 | up | malonyl-CoA decarboxylase CDS | Pden_1720 | PDEN_RS08455 |
| cds-WP_011750419.1 | *ugpB* | 1.17588E-26 | 26 | 1.81022E-28 | 2.4 | up | ugpB CDS (glycerol-3-p permease) | Pden_4190 | PDEN_RS20875 |
| cds-WP_011747674.1 | *-* | 3.06796E-07 | 7 | 3.93141E-08 | 2.4 | up | DUF1852 domain-containing protein CDS | Pden_1354 | PDEN_RS06685 |
| cds-WP_128492935.1 | *-* | 2.92407E-12 | 12 | 1.864E-13 | 2.4 | up | hypothetical protein CDS | Pden_1356 | PDEN_RS26870 |
| cds-WP_011747140.1 | *-* | 4.98541E-07 | 6 | 6.6155E-08 | 2.4 | up | ABC transporter ATP-binding protein CDS | Pden_0800 | PDEN_RS03980 |
| cds-WP_011750862.1 | *-* | 3.99193E-18 | 17 | 1.21177E-19 | 2.4 | up | hypothetical protein CDS | Pden_4642 | PDEN_RS23110 |
| cds-WP_011751042.1 | *-* | 8.26721E-07 | 6 | 1.14992E-07 | 2.4 | up | SDR family oxidoreductase CDS | Pden_4822 | PDEN_RS24005 |
| cds-WP_011750489.1 | *guaD* | 5.69731E-12 | 11 | 3.74303E-13 | 2.4 | up | guaD CDS | Pden_4260 | PDEN_RS21220 |
| cds-WP_011747600.1 | *-* | 5.66681E-23 | 22 | 1.16727E-24 | 2.4 | up | purine permease CDS | Pden_1277 | PDEN_RS06305 |
| cds-WP_011750082.1 | *-* | 2.45053E-12 | 12 | 1.54619E-13 | 2.4 | up | site-specific integrase CDS | Pden_3849 | PDEN_RS19160 |
| cds-WP_011750861.1 | *-* | 1.49889E-10 | 10 | 1.17649E-11 | 2.4 | up | hypothetical protein CDS | Pden_4641 | PDEN_RS23105 |
| cds-WP_011751214.1 | *-* | 2.19471E-08 | 8 | 2.32224E-09 | 2.4 | up | acetate--CoA ligase family protein CDS | Pden_4996 | PDEN_RS24845 |
| cds-WP_011749717.1 | *-* | 8.16135E-16 | 15 | 3.25605E-17 | 2.3 | up | hypothetical protein CDS | Pden_3470 | PDEN_RS17190 |
| cds-WP_011750980.1 | *lpdA* | 3.72321E-06 | 5 | 5.7156E-07 | 2.3 | up | lpdA CDS | Pden_4760 | PDEN_RS23695 |
| cds-WP_011748944.1 | *-* | 3.69179E-10 | 9 | 3.09784E-11 | 2.3 | up | LysR family transcriptional regulator CDS | Pden_2664 | PDEN_RS13260 |
| cds-WP_011750771.1 | *-* | 9.48495E-12 | 11 | 6.49879E-13 | 2.3 | up | glutamine synthetase CDS | Pden_4547 | PDEN_RS22650 |
| cds-WP_011749534.1 | *-* | 1.38238E-09 | 9 | 1.2439E-10 | 2.3 | up | LysR family transcriptional regulator CDS | Pden_3271 | PDEN_RS16245 |
| cds-WP_011750772.1 | *-* | 6.89098E-08 | 7 | 7.97871E-09 | 2.3 | up | aldehyde dehydrogenase family protein CDS | Pden_4548 | PDEN_RS22655 |
| cds-WP_011746947.1 | *-* | 4.88398E-14 | 13 | 2.35092E-15 | 2.3 | up | hypothetical protein CDS | Pden_0602 | PDEN_RS02995 |
| cds-WP_041530454.1 | *-* | 3.6689E-08 | 7 | 4.10484E-09 | 2.3 | up | glycosyltransferase family 2 protein CDS | Pden_4162 | PDEN_RS20735 |
| cds-WP_011747779.1 | *-* | 8.09818E-05 | 4 | 1.56275E-05 | 2.3 | up | GNAT family N-acetyltransferase CDS | Pden_1461 | PDEN_RS07200 |
| cds-WP_011748633.1 | *-* | 5.56367E-05 | 4 | 1.05193E-05 | 2.3 | up | hypothetical protein CDS | Pden_2350 | PDEN_RS11670 |
| cds-WP_011751039.1 | *-* | 9.28236E-10 | 9 | 8.21162E-11 | 2.3 | up | acetyl-CoA acetyltransferase CDS | Pden_4819 | PDEN_RS23990 |
| cds-WP_011748185.1 | *msrB* | 1.28044E-05 | 5 | 2.14332E-06 | 2.3 | up | msrB CDS | Pden_1893 | PDEN_RS09355 |
| cds-WP_128493090.1 | *-* | 2.5118E-06 | 6 | 3.74156E-07 | 2.3 | up | hypothetical protein CDS | Pden_3848 | PDEN_RS19155 |
| cds-WP_011748543.1 | *-* | 9.43538E-11 | 10 | 7.16121E-12 | 2.2 | up | NAD-dependent succinate-semialdehyde dehydrogenase CDS | Pden_2257 | PDEN_RS11220 |
| cds-WP_011750493.1 | *-* | 7.28019E-23 | 22 | 1.51539E-24 | 2.2 | up | sigma-54-dependent Fis family transcriptional regulator CDS | Pden_4264 | PDEN_RS21240 |
| cds-WP_011749132.1 | *-* | 2.86647E-10 | 10 | 2.35558E-11 | 2.2 | up | formate dehydrogenase beta subunit CDS | Pden_2857 | PDEN_RS14225 |
| cds-WP_041529877.1 | *-* | 2.15456E-10 | 10 | 1.73318E-11 | 2.2 | up | LacI family DNA-binding transcriptional regulator CDS | Pden_1685 | PDEN_RS08280 |
| cds-WP_011749587.1 | *-* | 1.56436E-05 | 5 | 2.66945E-06 | 2.2 | up | recombinase family protein CDS | Pden_0106 | PDEN_RS00535 |
| cds-WP_011749390.1 | *-* | 1.1514E-08 | 8 | 1.17337E-09 | 2.2 | up | tyrosine-type recombinase/integrase CDS | Pden_3122 | PDEN_RS15545 |
| cds-WP_011750707.1 | *mmsB* | 1.76933E-11 | 11 | 1.26216E-12 | 2.2 | up | 3-hydroxyisobutyrate dehydrogenase | Pden_4482 | PDEN_RS22335 |
| cds-WP_011748636.1 | *-* | 3.39695E-08 | 7 | 3.76375E-09 | 2.2 | up | FIST C-terminal domain-containing protein CDS | Pden_2353 | PDEN_RS11685 |
| cds-WP_074810797.1 | *-* | 5.71194E-13 | 12 | 3.31917E-14 | 2.2 | up | hypothetical protein CDS | Pden_0973 | PDEN_RS04850 |
| cds-WP_128493044.1 | *-* | 2.07601E-05 | 5 | 3.60555E-06 | 2.2 | up | hypothetical protein CDS | Pden_3322 | PDEN_RS16495 |
| cds-WP_011747100.1 | *-* | 4.53575E-10 | 9 | 3.84536E-11 | 2.2 | up | hypothetical protein CDS | Pden_0757 | PDEN_RS03760 |
| cds-WP_157137424.1 | *-* | 1.35322E-20 | 20 | 3.37425E-22 | 2.2 | up | hypothetical protein CDS |  | PDEN_RS26815 |
| cds-WP_011747881.1 | *-* | 1.44805E-15 | 15 | 5.90274E-17 | 2.2 | up | TRAP transporter substrate-binding protein CDS | Pden_1563 | PDEN_RS07680 |
| cds-WP_011747751.1 | *-* | 8.98662E-05 | 4 | 1.75757E-05 | 2.2 | up | biotin transporter BioY CDS | Pden_1432 | PDEN_RS07080 |
| cds-WP_157137429.1 | *-* | 8.70516E-06 | 5 | 1.41374E-06 | 2.2 | up | hypothetical protein CDS |  | PDEN_RS26875 |
| cds-WP_011750416.1 | *-* | 1.88572E-09 | 9 | 1.73771E-10 | 2.2 | up | sn-glycerol-3-phosphate import ATP-binding protein UgpC CDS | Pden_4187 | PDEN_RS20860 |
| cds-WP_011748634.1 | *-* | 4.83278E-06 | 5 | 7.54467E-07 | 2.2 | up | hypothetical protein CDS | Pden_2351 | PDEN_RS11675 |
| cds-WP_011750486.1 | *xdhA* | 3.23361E-08 | 7 | 3.55473E-09 | 2.2 | up | xanthine dehydrogenase small subunit | Pden_4257 | PDEN_RS21205 |
| cds-WP_011749823.1 | *-* | 8.16135E-16 | 15 | 3.25456E-17 | 2.2 | up | transglutaminase domain-containing protein CDS | Pden_3586 | PDEN_RS17770 |
| cds-WP_011751037.1 | *-* | 9.94574E-12 | 11 | 6.92234E-13 | 2.2 | up | TRAP transporter substrate-binding protein CDS | Pden_4817 | PDEN_RS23980 |
| cds-WP_011746571.1 | *-* | 1.80288E-21 | 21 | 4.22184E-23 | 2.2 | up | sigma-54-dependent Fis family transcriptional regulator CDS | Pden_0224 | PDEN_RS01080 |
| cds-WP_011749101.1 | *fdhE* | 4.08026E-09 | 8 | 3.97233E-10 | 2.2 | up | fdhE CDS | Pden_2826 | PDEN_RS14070 |
| cds-WP_198140519.1 | *-* | 3.56041E-12 | 11 | 2.29281E-13 | 2.1 | up | Na/Pi symporter CDS | Pden_0816 | PDEN_RS04055 |
| cds-WP_011749131.1 | *fdhF* | 2.5938E-15 | 15 | 1.07981E-16 | 2.1 | up | formate dehydrogenase subunit alpha | Pden_2856 | PDEN_RS14220 |
| cds-WP_011749201.1 | *-* | 3.42724E-17 | 16 | 1.13696E-18 | 2.1 | up | L-lactate permease CDS | Pden_2927 | PDEN_RS14580 |
| cds-WP_011749696.1 | *-* | 3.18621E-23 | 22 | 6.494E-25 | 2.1 | up | multidrug efflux RND transporter permease subunit CDS | Pden_3448 | PDEN_RS17080 |
| cds-WP_011746900.1 | *-* | 2.84316E-37 | 37 | 2.65082E-39 | 2.1 | up | 2-oxoglutarate dehydrogenase E1 component CDS | Pden_0555 | PDEN_RS02770 |
| cds-WP_011746873.1 | *-* | 2.76775E-14 | 14 | 1.30826E-15 | 2.1 | up | BMP family ABC transporter substrate-binding protein CDS | Pden_0528 | PDEN_RS02630 |
| cds-WP_011748622.1 | *-* | 1.33414E-07 | 7 | 1.60548E-08 | 2.1 | up | 4-aminobutyrate--2-oxoglutarate transaminase CDS | Pden_2338 | PDEN_RS11610 |
| cds-WP_011749743.1 | *-* | 1.36678E-10 | 10 | 1.06687E-11 | 2.1 | up | TRAP transporter substrate-binding protein CDS | Pden_3497 | PDEN_RS17330 |
| cds-WP_011751040.1 | *-* | 4.57072E-13 | 12 | 2.61637E-14 | 2.1 | up | acyl-CoA synthetase CDS | Pden_4820 | PDEN_RS23995 |
| cds-WP_011749869.1 | *-* | 1.64513E-15 | 15 | 6.77741E-17 | 2.1 | up | isovaleryl-CoA dehydrogenase CDS | Pden_3633 | PDEN_RS18015 |
| cds-WP_011750239.1 | *-* | 9.57349E-08 | 7 | 1.12715E-08 | 2.1 | up | DUF2945 domain-containing protein CDS | Pden_4005 | PDEN_RS19945 |
| cds-WP_011747578.1 | *-* | 8.65655E-09 | 8 | 8.65279E-10 | 2.1 | up | SDR family oxidoreductase CDS | Pden_1255 | PDEN_RS06195 |
| cds-WP_011746872.1 | *-* | 9.5675E-09 | 8 | 9.60484E-10 | 2.1 | up | ABC transporter ATP-binding protein CDS | Pden_0527 | PDEN_RS02625 |
| cds-WP_041529795.1 | *-* | 9.51651E-07 | 6 | 1.33503E-07 | 2.1 | up | hypothetical protein CDS | Pden_0978 | PDEN_RS04875 |
| cds-WP_011747142.1 | *-* | 6.56117E-05 | 4 | 1.25334E-05 | 2.1 | up | ABC transporter permease CDS | Pden_0802 | PDEN_RS03990 |
| cds-WP_011750101.1 | *-* | 4.08146E-10 | 9 | 3.43366E-11 | 2.1 | up | zinc metallopeptidase CDS | Pden_3868 | PDEN_RS19245 |
| cds-WP_041529728.1 | *-* | 4.24381E-08 | 7 | 4.78487E-09 | 2.0 | up | hypothetical protein CDS | Pden_0347 | PDEN_RS01705 |
| cds-WP_011750741.1 | *-* | 3.14314E-10 | 10 | 2.58975E-11 | 2.0 | up | VOC family protein CDS | Pden_4516 | PDEN_RS22510 |
| cds-WP_128492954.1 | *-* | 5.9106E-10 | 9 | 5.10065E-11 | 2.0 | up | tyrosine-type recombinase/integrase CDS | Pden_2419 | PDEN_RS12025 |
| cds-WP_011746899.1 | *odhB* | 6.06734E-35 | 34 | 6.05156E-37 | 2.0 | up | 2-oxoglutarate dehydrogenase complex dihydrolipoyllysine-residue succinyltransferase | Pden_0554 | PDEN_RS02765 |
| cds-WP_104493368.1 | *-* | 9.22046E-12 | 11 | 6.26583E-13 | 2.0 | up | hypothetical protein CDS | Pden_0393 | PDEN_RS01940 |
| cds-WP_011749712.1 | *-* | 2.10941E-05 | 5 | 3.66814E-06 | 2.0 | up | Rieske 2Fe-2S domain-containing protein CDS | Pden_3465 | PDEN_RS17165 |
| cds-WP_011749575.1 | *-* | 3.78018E-07 | 6 | 4.94243E-08 | 2.0 | up | hypothetical protein CDS | Pden_3314 | PDEN_RS16455 |
| cds-WP_081465042.1 | *-* | 3.96294E-06 | 5 | 6.11799E-07 | 2.0 | up | rhomboid family intramembrane serine protease CDS | Pden_2337 | PDEN_RS11605 |
| cds-WP_041529953.1 | *-* | 1.12582E-09 | 9 | 1.0106E-10 | 2.0 | up | Arc family DNA-binding protein CDS | Pden_2281 | PDEN_RS26955 |
| cds-WP_164901601.1 | *-* | 5.09035E-05 | 4 | 9.54717E-06 | 2.0 | up | hypothetical protein CDS |  | PDEN_RS27300 |
| cds-WP_011747299.1 | *phaR* | 2.14211E-21 | 21 | 5.1091E-23 | 2.0 | up | polyhydroxyalkanoate synthesis repressor PhaR | Pden_0960 | PDEN_RS04775 |
| cds-WP_011748334.1 | *-* | 6.65088E-08 | 7 | 7.67188E-09 | -2.0 | down | TonB-dependent receptor CDS | Pden_2046 | PDEN_RS10170 |
| cds-WP_011747230.1 | *rpsF* | 1.62712E-26 | 26 | 2.54017E-28 | -2.0 | down | rpsF CDS | Pden_0891 | PDEN_RS04425 |
| cds-WP_011748873.1 | *-* | 1.56791E-13 | 13 | 8.2951E-15 | -2.0 | down | RNA-binding protein CDS | Pden_2593 | PDEN_RS12885 |
| cds-WP_011749934.1 | *-* | 3.25276E-07 | 6 | 4.18232E-08 | -2.0 | down | RNA methyltransferase CDS | Pden_3698 | PDEN_RS18360 |
| cds-WP_011748055.1 | *ilvC* | 2.75472E-10 | 10 | 2.25777E-11 | -2.0 | down | ketol-acid reductoisomerase | Pden_1760 | PDEN_RS08665 |
| cds-WP_011747229.1 | *-* | 7.58753E-13 | 12 | 4.53118E-14 | -2.0 | down | 30S ribosomal protein S18 CDS | Pden_0890 | PDEN_RS04420 |
| cds-WP_041530668.1 | *-* | 2.35576E-06 | 6 | 3.48869E-07 | -2.0 | down | hypothetical protein CDS | Pden_4302 | PDEN_RS21430 |
| cds-WP_041530731.1 | *-* | 6.49124E-19 | 18 | 1.88601E-20 | -2.0 | down | altronate dehydratase CDS | Pden_4928 | PDEN_RS24515 |
| cds-WP_010400236.1 | *rpsS* | 2.86461E-07 | 7 | 3.64597E-08 | -2.0 | down | 30S ribosomal protein S19 | Pden_0763 | PDEN_RS03790 |
| cds-WP_011748577.1 | *-* | 8.03332E-05 | 4 | 1.54849E-05 | -2.0 | down | glycosyl transferase CDS | Pden_2293 | PDEN_RS11390 |
| cds-WP_011747088.1 | *rplK* | 5.48385E-17 | 16 | 1.86679E-18 | -2.0 | down | 50S ribosomal protein L11 | Pden_0743 | PDEN_RS03695 |
| cds-WP_011747028.1 | *tolQ* | 9.96023E-13 | 12 | 6.04698E-14 | -2.0 | down | tolQ CDS | Pden_0683 | PDEN_RS03395 |
| cds-WP_011747109.1 | *rpsQ* | 1.96741E-07 | 7 | 2.423E-08 | -2.0 | down | 30S ribosomal protein S17 | Pden_0768 | PDEN_RS03815 |
| cds-WP_011750311.1 | *-* | 6.36432E-18 | 17 | 1.97333E-19 | -2.0 | down | 50S ribosomal protein L25/general stress proteinCtc CDS | Pden_4077 | PDEN_RS20310 |
| cds-WP_041530730.1 | *-* | 1.00529E-07 | 7 | 1.19099E-08 | -2.0 | down | amino acid ABC transporter permease CDS | Pden_4924 | PDEN_RS24495 |
| cds-WP_011750529.1 | *rpsD* | 7.40185E-13 | 12 | 4.39745E-14 | -2.1 | down | 30S ribosomal protein S4 | Pden_4303 | PDEN_RS21435 |
| cds-WP_011748890.1 | *-* | 2.60821E-06 | 6 | 3.89083E-07 | -2.1 | down | TonB-dependent receptor CDS | Pden_2610 | PDEN_RS12975 |
| cds-WP_104491135.1 | *-* | 5.30849E-09 | 8 | 5.23713E-10 | -2.1 | down | H-type lectin domain-containing protein CDS | Pden_3820 | PDEN_RS19005 |
| cds-WP_041529773.1 | *rpmC* | 7.36309E-13 | 12 | 4.35846E-14 | -2.1 | down | 50S ribosomal protein L29 | Pden_0767 | PDEN_RS03810 |
| cds-WP_011749035.1 | *rpsO* | 1.66471E-10 | 10 | 1.31387E-11 | -2.1 | down | 30S ribosomal protein S15 | Pden_2760 | PDEN_RS13740 |
| cds-WP_011750809.1 | *-* | 1.94114E-09 | 9 | 1.79299E-10 | -2.1 | down | co-chaperone GroES CDS | Pden_4586 | PDEN_RS22840 |
| cds-WP_011747105.1 | *rplV* | 3.39651E-10 | 9 | 2.82061E-11 | -2.1 | down | 50S ribosomal protein L22 | Pden_0764 | PDEN_RS03795 |
| cds-WP_011751150.1 | *-* | 1.12779E-05 | 5 | 1.86824E-06 | -2.1 | down | SDR family oxidoreductase CDS | Pden_4932 | PDEN_RS24535 |
| cds-WP_011747103.1 | *-* | 1.04265E-13 | 13 | 5.35793E-15 | -2.1 | down | 50S ribosomal protein L23 CDS | Pden_0761 | PDEN_RS03780 |
| cds-WP_010400243.1 | *rplN* | 1.79749E-20 | 20 | 4.55998E-22 | -2.1 | down | rplN CDS | Pden_0769 | PDEN_RS03820 |
| cds-WP_011747115.1 | *rplR* | 1.77872E-14 | 14 | 8.3305E-16 | -2.1 | down | rplR CDS | Pden_0775 | PDEN_RS03850 |
| cds-WP_041529977.1 | *-* | 4.64011E-11 | 10 | 3.42072E-12 | -2.1 | down | non-ribosomal peptide synthetase CDS | Pden_2564 | PDEN_RS12745 |
| cds-WP_011748559.1 | *rplT* | 1.39224E-17 | 17 | 4.43754E-19 | -2.1 | down | 50S ribosomal protein L20 | Pden_2273 | PDEN_RS11300 |
| cds-WP_011748041.1 | *ndk* | 9.24805E-22 | 21 | 2.12553E-23 | -2.1 | down | nucleoside-diphosphate kinase | Pden_1746 | PDEN_RS08590 |
| cds-WP_041530417.1 | *-* | 3.39964E-06 | 5 | 5.17465E-07 | -2.1 | down | hypothetical protein CDS |  | PDEN_RS19080 |
| cds-WP_041530427.1 | *-* | 1.52466E-09 | 9 | 1.37854E-10 | -2.1 | down | chorismate mutase CDS | Pden_3880 | PDEN_RS19305 |
| cds-WP_011747113.1 | *rpsH* | 1.50647E-19 | 19 | 4.08301E-21 | -2.1 | down | 30S ribosomal protein S8 | Pden_0773 | PDEN_RS03840 |
| cds-WP_011746353.1 | *mnmE* | 5.01848E-15 | 14 | 2.17627E-16 | -2.1 | down | tRNA uridine-5-carboxymethylaminomethyl(34) synthesis GTPase MnmE | Pden_0003 | PDEN_RS00020 |
| cds-WP_011747114.1 | *rplF* | 8.58302E-24 | 23 | 1.6377E-25 | -2.1 | down | 50S ribosomal protein L6 | Pden_0774 | PDEN_RS03845 |
| cds-WP_011750114.1 | *rpsP* | 1.87962E-27 | 27 | 2.64908E-29 | -2.1 | down | 30S ribosomal protein S16 | Pden_3881 | PDEN_RS19310 |
| cds-WP_011749243.1 | *rplM* | 7.8E-29 | 28 | 1.03166E-30 | -2.1 | down | 50S ribosomal protein L13 | Pden_2969 | PDEN_RS14790 |
| cds-WP_011750112.1 | *-* | 9.79608E-07 | 6 | 1.38063E-07 | -2.1 | down | GNAT family N-acetyltransferase CDS | Pden_3879 | PDEN_RS19300 |
| cds-WP_011749491.1 | *-* | 4.78384E-15 | 14 | 2.0434E-16 | -2.1 | down | enoyl-CoA hydratase/isomerase family protein CDS | Pden_3225 | PDEN_RS16020 |
| cds-WP_011747231.1 | *-* | 3.199E-10 | 9 | 2.64272E-11 | -2.2 | down | polyisoprenoid-binding protein CDS | Pden_0892 | PDEN_RS04430 |
| cds-WP_011749482.1 | *-* | 5.07436E-14 | 13 | 2.46998E-15 | -2.2 | down | NAD(P)-dependent oxidoreductase CDS | Pden_3256 | PDEN_RS16165 |
| cds-WP_011749481.1 | *-* | 4.4001E-16 | 15 | 1.62189E-17 | -2.2 | down | carboxymuconolactone decarboxylase family protein CDS | Pden_3257 | PDEN_RS16170 |
| cds-WP_011747030.1 | *tolA* | 1.28906E-12 | 12 | 7.88194E-14 | -2.2 | down | cell envelope integrity protein TolA | Pden_0685 | PDEN_RS03405 |
| cds-WP_011747029.1 | *-* | 9.12503E-11 | 10 | 6.90511E-12 | -2.2 | down | ExbD/TolR family protein CDS | Pden_0684 | PDEN_RS03400 |
| cds-WP_011750115.1 | *rimM* | 1.00562E-33 | 33 | 1.04661E-35 | -2.2 | down | 16S rRNA processing protein RimM | Pden_3882 | PDEN_RS19315 |
| cds-WP_011751144.1 | *-* | 1.27925E-06 | 6 | 1.8279E-07 | -2.2 | down | amino acid ABC transporter ATP-binding protein CDS | Pden_4926 | PDEN_RS24505 |
| cds-WP_011748418.1 | *-* | 4.72334E-10 | 9 | 4.02488E-11 | -2.2 | down | GNAT family N-acetyltransferase CDS | Pden_2131 | PDEN_RS10585 |
| cds-WP_011749734.1 | *-* | 4.17521E-06 | 5 | 6.48189E-07 | -2.2 | down | FAD-dependent monooxygenase CDS | Pden_3488 | PDEN_RS17285 |
| cds-WP_011747112.1 | *rpsN* | 6.33318E-15 | 14 | 2.80132E-16 | -2.2 | down | 30S ribosomal protein S14 | Pden_0772 | PDEN_RS03835 |
| cds-WP_011749475.1 | *dctP* | 2.00951E-08 | 8 | 2.11756E-09 | -2.2 | down | TRAP transporter substrate-binding protein DctP | Pden_3209 | PDEN_RS15945 |
| cds-WP_011746516.1 | *-* | 7.18256E-13 | 12 | 4.23603E-14 | -2.2 | down | cupin domain-containing protein CDS | Pden_0167 | PDEN_RS00805 |
| cds-WP_011750335.1 | *-* | 4.7603E-27 | 26 | 6.91545E-29 | -2.2 | down | GNAT family N-acetyltransferase CDS | Pden_4101 | PDEN_RS20440 |
| cds-WP_011746938.1 | *-* | 1.06554E-09 | 9 | 9.51867E-11 | -2.2 | down | hypothetical protein CDS | Pden_0593 | PDEN_RS02955 |
| cds-WP_011747080.1 | *-* | 4.42051E-16 | 15 | 1.639E-17 | -2.3 | down | dienelactone hydrolase family protein CDS | Pden_0735 | PDEN_RS03650 |
| cds-WP_011747110.1 | *-* | 3.64938E-15 | 14 | 1.54299E-16 | -2.3 | down | 50S ribosomal protein L24 CDS | Pden_0770 | PDEN_RS03825 |
| cds-WP_011750599.1 | *-* | 1.11461E-05 | 5 | 1.84275E-06 | -2.3 | down | TonB-dependent siderophore receptor CDS | Pden_4373 | PDEN_RS21785 |
| cds-WP_011747395.1 | *-* | 2.50982E-06 | 6 | 3.73317E-07 | -2.3 | down | hypothetical protein CDS | Pden_1066 | PDEN_RS05295 |
| cds-WP_011748329.1 | *-* | 1.05151E-31 | 31 | 1.20837E-33 | -2.3 | down | aspartate aminotransferase family protein CDS | Pden_2041 | PDEN_RS10145 |
| cds-WP_011749962.1 | *rpsB* | 2.85785E-26 | 26 | 4.58545E-28 | -2.3 | down | 30S ribosomal protein S2 | Pden_3726 | PDEN_RS18490 |
| cds-WP_011750900.1 | *-* | 3.40184E-07 | 6 | 4.4091E-08 | -2.3 | down | MaoC family dehydratase CDS | Pden_4680 | PDEN_RS23300 |
| cds-WP_011747117.1 | *rpmD* | 3.34279E-16 | 15 | 1.21042E-17 | -2.3 | down | 50S ribosomal protein L30 | Pden_0777 | PDEN_RS03860 |
| cds-WP_011750640.1 | *gabT* | 3.41155E-08 | 7 | 3.78733E-09 | -2.3 | down | 4-aminobutyrate--2-oxoglutarate transaminase | Pden_4414 | PDEN_RS21980 |
| cds-WP_019353022.1 | *groES* | 3.56599E-10 | 9 | 2.97681E-11 | -2.3 | down | co-chaperone GroES |  | PDEN_RS18130 |
| cds-WP_011749493.1 | *-* | 6.23656E-14 | 13 | 3.05608E-15 | -2.4 | down | CoA transferase CDS | Pden_3227 | PDEN_RS16030 |
| cds-WP_041530483.1 | *-* | 3.94797E-15 | 14 | 1.6778E-16 | -2.4 | down | DUF1775 domain-containing protein CDS | Pden_4444 | PDEN_RS22135 |
| cds-WP_011749303.1 | *-* | 6.3387E-06 | 5 | 1.00468E-06 | -2.4 | down | ABC transporter substrate-binding protein CDS | Pden_3033 | PDEN_RS15130 |
| cds-WP_011749492.1 | *-* | 2.61895E-16 | 16 | 9.31284E-18 | -2.4 | down | thiolase family protein CDS | Pden_3226 | PDEN_RS16025 |
| cds-WP_011747619.1 | *-* | 3.76874E-07 | 6 | 4.91931E-08 | -2.4 | down | DegT/DnrJ/EryC1/StrS family aminotransferase CDS | Pden_1296 | PDEN_RS06400 |
| cds-WP_011750312.1 | *trmFO* | 6.17576E-17 | 16 | 2.11572E-18 | -2.4 | down | methylenetetrahydrofolate--tRNA-(uracil(54)- C(5))-methyltransferase (FADH(2)-oxidizing) TrmFO | Pden_4078 | PDEN_RS20315 |
| cds-WP_011747084.1 | *-* | 6.95284E-27 | 26 | 1.04021E-28 | -2.4 | down | ABC transporter ATP-binding protein CDS | Pden_0739 | PDEN_RS03670 |
| cds-WP_011750569.1 | *cysT* | 2.98425E-06 | 6 | 4.48414E-07 | -2.4 | down | sulfate ABC transporter permease subunit CysT | Pden_4343 | PDEN_RS21630 |
| cds-WP_198140538.1 | *-* | 6.71859E-16 | 15 | 2.59304E-17 | -2.4 | down | AMP-binding protein CDS | Pden_3230 | PDEN_RS16045 |
| cds-WP_011751149.1 | *-* | 1.80674E-09 | 9 | 1.66101E-10 | -2.4 | down | zinc-binding alcohol dehydrogenase family protein CDS | Pden_4931 | PDEN_RS24530 |
| cds-WP_011749495.1 | *-* | 2.28546E-16 | 16 | 8.02786E-18 | -2.4 | down | MaoC family dehydratase N-terminal domain-containing protein CDS | Pden_3229 | PDEN_RS16040 |
| cds-WP_011747111.1 | *rplE* | 1.40972E-11 | 11 | 9.96462E-13 | -2.5 | down | rplE CDS | Pden_0771 | PDEN_RS03830 |
| cds-WP_011750669.1 | *-* | 3.24357E-15 | 14 | 1.36438E-16 | -2.5 | down | SCO family protein CDS | Pden_4443 | PDEN_RS22130 |
| cds-WP_011750945.1 | *-* | 5.97674E-07 | 6 | 8.06057E-08 | -2.5 | down | hypothetical protein CDS | Pden_4725 | PDEN_RS23520 |
| cds-WP_011749961.1 | *-* | 3.7282E-39 | 38 | 3.23348E-41 | -2.5 | down | elongation factor Ts CDS | Pden_3725 | PDEN_RS18485 |
| cds-WP_011746417.1 | *tnpB* | 2.53926E-08 | 8 | 2.74188E-09 | -2.5 | down | IS66 family insertion sequence element accessoryprotein TnpB | Pden_0067 | PDEN_RS00375 |
| cds-WP_011747189.1 | *-* | 4.2325E-25 | 24 | 7.43348E-27 | -2.5 | down | porin family protein CDS | Pden_0849 | PDEN_RS04220 |
| cds-WP_011746845.1 | *-* | 1.7296E-09 | 9 | 1.57884E-10 | -2.5 | down | VOC family protein CDS | Pden_0500 | PDEN_RS02485 |
| cds-PDEN_RS14835 | *-* | 9.02857E-06 | 5 | 1.47018E-06 | -2.5 | down | ? | Pden_2973 | PDEN_RS14835 |
| cds-WP_011751143.1 | *-* | 2.50718E-07 | 7 | 3.14757E-08 | -2.6 | down | amino acid ABC transporter permease CDS | Pden_4925 | PDEN_RS24500 |
| cds-WP_011750484.1 | *-* | 1.07338E-05 | 5 | 1.76646E-06 | -2.6 | down | Hsp70 family protein CDS | Pden_4255 | PDEN_RS21195 |
| cds-WP_011747262.1 | *-* | 5.76323E-16 | 15 | 2.16184E-17 | -2.6 | down | hypothetical protein CDS | Pden_0923 | PDEN_RS04585 |
| cds-WP_011747154.1 | *-* | 2.90382E-19 | 19 | 8.12215E-21 | -2.7 | down | phosphoglycerate dehydrogenase CDS | Pden_0814 | PDEN_RS04045 |
| cds-WP_104494388.1 | *-* | 1.52458E-06 | 6 | 2.2115E-07 | -2.7 | down | TetR family transcriptional regulator CDS | Pden_2940 | PDEN_RS14645 |
| cds-WP_011749494.1 | *-* | 8.69252E-21 | 20 | 2.12978E-22 | -2.7 | down | aldehyde dehydrogenase CDS | Pden_3228 | PDEN_RS16035 |
| cds-WP_011749216.1 | *-* | 1.0367E-39 | 39 | 8.5418E-42 | -2.8 | down | multidrug efflux RND transporter permease subunit CDS | Pden_2942 | PDEN_RS14655 |
| cds-WP_011747531.1 | *ureC* | 3.40184E-07 | 6 | 4.41089E-08 | -2.8 | down | urease subunit alpha | Pden_1208 | PDEN_RS05985 |
| cds-WP_041529781.1 | *-* | 2.97041E-07 | 7 | 3.78992E-08 | -2.8 | down | hypothetical protein CDS |  | PDEN_RS04285 |
| cds-WP_011748469.1 | *-* | 7.60771E-15 | 14 | 3.38157E-16 | -2.9 | down | DUF1800 domain-containing protein CDS | Pden_2182 | PDEN_RS10840 |
| cds-WP_011747118.1 | *-* | 1.8687E-08 | 8 | 1.95703E-09 | -2.9 | down | hypothetical protein CDS | Pden_0778 | PDEN_RS03865 |
| cds-WP_128492943.1 | *-* | 4.32341E-19 | 18 | 1.22803E-20 | -2.9 | down | ammonium transporter CDS | Pden_2032 | PDEN_RS10105 |
| cds-WP_011749215.1 | *-* | 1.18288E-07 | 7 | 1.41507E-08 | -3.0 | down | efflux RND transporter periplasmic adaptor subunit CDS | Pden_2941 | PDEN_RS14650 |
| cds-WP_011750899.1 | *-* | 1.09018E-23 | 23 | 2.10377E-25 | -3.0 | down | 6,7-dimethyl-8-ribityllumazine synthase CDS | Pden_4679 | PDEN_RS23295 |
| cds-WP_011750642.1 | *-* | 1.61074E-38 | 38 | 1.46685E-40 | -3.0 | down | NAD-dependent succinate-semialdehyde dehydrogenase CDS | Pden_4416 | PDEN_RS21990 |
| cds-WP_011750125.1 | *pdhA* | 2.83234E-40 | 40 | 2.27226E-42 | -3.2 | down | pyruvate dehydrogenase (acetyl-transferring) E1 component subunit alpha | Pden_3892 | PDEN_RS19370 |
| cds-WP_011749301.1 | *-* | 2.84838E-06 | 6 | 4.26145E-07 | -3.3 | down | iron ABC transporter permease CDS | Pden_3031 | PDEN_RS15120 |
| cds-WP_011750124.1 | *-* | 3.9234E-44 | 43 | 2.63715E-46 | -3.4 | down | pyruvate dehydrogenase complex E1 component subunit beta CDS | Pden_3891 | PDEN_RS19365 |
| cds-WP_011750123.1 | *-* | 1.41783E-52 | 52 | 7.68553E-55 | -3.5 | down | pyruvate dehydrogenase complex dihydrolipoamide acetyltransferase CDS | Pden_3890 | PDEN_RS19360 |
| cds-WP_011749300.1 | *-* | 4.60306E-09 | 8 | 4.50125E-10 | -3.5 | down | class I SAM-dependent methyltransferase CDS | Pden_3030 | PDEN_RS15115 |
| cds-WP_011748558.1 | *-* | 3.00914E-05 | 5 | 5.38278E-06 | -3.6 | down | cytochrome P450 CDS | Pden_2272 | PDEN_RS11295 |
| cds-WP_011747493.1 | *-* | 1.13413E-05 | 5 | 1.88119E-06 | -4.1 | down | ABC transporter substrate-binding protein CDS | Pden_1168 | PDEN_RS05785 |
| cds-WP_011748511.1 | *-* | 5.21305E-57 | 56 | 2.48671E-59 | -4.1 | down | NADP-dependent malic enzyme CDS | Pden_2224 | PDEN_RS11050 |
| cds-WP_164901616.1 | *-* | 8.65261E-35 | 34 | 8.81771E-37 | -4.4 | down | TonB-dependent siderophore receptor CDS | Pden_3029 | PDEN_RS15110 |
| cds-WP_011750985.1 | *-* | 1.2001E-101 | 101 | 3.1225E-104 | -9.6 | down | dicarboxylate/amino acid:cation symporter CDS | Pden_4765 | PDEN_RS23720 |

Supplementary Table 3: Top regulated genes KO_Ac_/WT_Ac_. Listed are all genes regulated above a threshold of log_2_-fold 2.0 in Pd1222 ΔramB on acetate in relation to Pd1222 wildtype on acetate.

| gene_id | Gene | padj | neg. log10(padjust) | pvalue | log2FoldChange | regulation | CDS/product description | Old locus tag | New locus tag |
| --- | --- | --- | --- | --- | --- | --- | --- | --- | --- |
| cds-WP_011747670.1 | *prpD* | 4.30972E-85 | 84 | 1.40169E-87 | 13.3 | up | MmgE/PrpD family protein CDS | Pden_1348 |  |
| cds-WP_041530157.1 | *prpB* | 1.85554E-33 | 33 | 1.97141E-35 | 12.7 | up | prpB CDS | Pden_1346 |  |
| cds-WP_011747669.1 | *prpC* | 1.37001E-91 | 91 | 4.15876E-94 | 12.6 | up | prpC | Pden_1347 |  |
| cds-PDEN_RS26015 | *-* | 4.46679E-24 | 23 | 8.23238E-26 | 12.4 | up | transcriptional repressor prp-operon | - | PDEN_RS26015 |
| cds-WP_011747671.1 | *-* | 5.70502E-22 | 21 | 1.28648E-23 | 12.0 | up | acyl-CoA thioesterase CDS | Pden_1349 | PDEN_RS06665 |
| cds-WP_011750346.1 | *-* | 7.43866E-16 | 15 | 2.91934E-17 | 10.2 | up | VOC family protein CDS | Pden_4115 | PDEN_RS20505 |
| cds-PDEN_RS20500 | *-* | 4.0324E-116 | 115 | 9.6177E-119 | 9.0 | up | acyl-CoA/acyl-ACP dehydrogenase CDS | Pden_4113 | PDEN_RS20500 |
| cds-WP_049792306.1 | *-* | 4.03678E-50 | 49 | 2.27572E-52 | 8.8 | up | hypothetical protein CDS | Pden_1351 | PDEN_RS06675 |
| cds-WP_011750347.1 | *-* | 3.70507E-72 | 71 | 1.28537E-74 | 8.3 | up | hypothetical protein CDS | Pden_4116 | PDEN_RS20510 |
| cds-WP_011750351.1 | *-* | 6.5218E-120 | 119 | 1.4141E-122 | 7.8 | up | TRAP transporter large permease subunit CDS | Pden_4120 | PDEN_RS20530 |
| cds-WP_198140555.1 | *-* | 4.8799E-101 | 100 | 1.3755E-103 | 7.7 | up | TRAP transporter small permease subunit CDS | Pden_4121 | PDEN_RS20535 |
| cds-WP_011750353.1 | *-* | 5.4393E-142 | 141 | 8.2557E-145 | 7.4 | up | TRAP transporter substrate-binding protein CDS | Pden_4122 | PDEN_RS20540 |
| cds-WP_011750439.1 | *-* | 2.9281E-63 | 63 | 1.33326E-65 | 7.3 | up | response regulator CDS | Pden_4210 | PDEN_RS20975 |
| cds-WP_011750635.1 | *-* | 1.61018E-13 | 13 | 8.55364E-15 | 7.0 | up | hydantoinase B/oxoprolinase family protein CDS | Pden_4409 | PDEN_RS21955 |
| cds-WP_011750345.1 | *-* | 1.94225E-15 | 15 | 8.0436E-17 | 6.8 | up | maoC family dehydratase | Pden_4111 | PDEN_RS20490 |
| cds-WP_011751204.1 | *-* | 5.17068E-07 | 6 | 6.9062E-08 | 6.7 | up | NAD(+)/NADH kinase CDS | Pden_4986 | PDEN_RS24795 |
| cds-WP_011750440.1 | *-* | 6.8547E-237 | 236 | 1.4863E-240 | 6.6 | up | cation acetate symporter CDS | Pden_4211 | PDEN_RS20980 |
| cds-WP_011750441.1 | *-* | 3.4732E-133 | 132 | 6.0246E-136 | 6.5 | up | DUF485 domain-containing protein CDS | Pden_4212 | PDEN_RS20985 |
| cds-WP_011746370.1 | *-* | 5.13834E-18 | 17 | 1.57092E-19 | 6.4 | up | methanol/ethanol family PQQ-dependent dehydrogenase CDS | Pden_0020 | PDEN_RS00105 |
| cds-WP_011750437.1 | *-* | 6.91563E-20 | 19 | 1.84437E-21 | 6.3 | up | acetate/propionate family kinase CDS | Pden_4208 | PDEN_RS20965 |
| cds-WP_011748741.1 | *-* | 1.67472E-07 | 7 | 2.04074E-08 | 6.2 | up | filamentous hemagglutinin N-terminal domain-containing protein CDS | Pden_2461 | PDEN_RS12235 |
| cds-WP_011746372.1 | *-* | 6.01885E-07 | 6 | 8.14346E-08 | 6.1 | up | substrate-binding domain-containing protein CDS | Pden_0022 | PDEN_RS00115 |
| cds-WP_011750350.1 | *-* | 4.48668E-39 | 38 | 3.98859E-41 | 6.0 | up | fumarat hydratase | Pden_4119 | PDEN_RS20525 |
| cds-WP_011750106.1 | *ccrA* | 2.5411E-229 | 229 | 1.6529E-232 | 6.0 | up | ccrA | Pden_3873 | PDEN_RS18165 |
| cds-WP_011751203.1 | *-* | 2.28313E-11 | 11 | 1.63767E-12 | 5.9 | up | thiamine pyrophosphate-dependent dehydrogenase E1 component subunit alpha CDS | Pden_4985 | PDEN_RS24790 |
| cds-WP_011750195.1 | *-* | 3.23154E-44 | 43 | 2.10204E-46 | 5.8 | up | coA transferase | Pden_4112 | PDEN_RS20495 |
| cds-WP_011750344.1 | *-* | 4.55829E-14 | 13 | 2.18426E-15 | 5.8 | up | coA ester lyase | Pden_4110 | PDEN_RS20485 |
| cds-WP_011751200.1 | *-* | 8.77269E-15 | 14 | 3.95646E-16 | 5.7 | up | glucose 1-dehydrogenase CDS | Pden_4982 | PDEN_RS24775 |
| cds-WP_011750438.1 | *-* | 8.75216E-69 | 68 | 3.60562E-71 | 5.6 | up | bifunctional enoyl-CoA hydratase/phosphate acetyltransferase CDS | Pden_4209 | PDEN_RS20970 |
| cds-WP_011750442.1 | *acs* | 1.1766E-180 | 180 | 1.0205E-183 | 5.6 | up | acetyl-CoA ligase | Pden_4213 | PDEN_RS20990 |
| cds-WP_041530425.1 | *-* | 2.2765E-231 | 231 | 9.8719E-235 | 5.6 | up | protein meaA CDS/mcm | Pden_3875 | PDEN_RS19280 |
| cds-WP_011748740.1 | *-* | 6.56687E-06 | 5 | 1.04369E-06 | 5.6 | up | ShlB/FhaC/HecB family hemolysin secretion/activation protein CDS | Pden_2460 | PDEN_RS12230 |
| cds-WP_011746374.1 | *-* | 2.45305E-17 | 17 | 7.92506E-19 | 5.5 | up | SRPBCC family protein CDS | Pden_0024 | PDEN_RS00125 |
| cds-WP_011750774.1 | *acs* | 9.1521E-172 | 171 | 9.9221E-175 | 5.5 | up | acetate coA ligase CDS | Pden_4550 | PDEN_RS22665 |
| cds-WP_041530124.1 | *hydA* | 4.9607E-14 | 13 | 2.39861E-15 | 5.5 | up | dihidropyrimidinase | Pden_1112 | PDEN_RS05510 |
| cds-WP_011749115.1 | *-* | 1.7495E-128 | 128 | 3.4141E-131 | 5.2 | up | acyl-CoA/acyl-ACP dehydrogenase CDS | Pden_2840 | PDEN_RS14140 |
| cds-WP_011747441.1 | *-* | 9.94657E-09 | 8 | 1.00285E-09 | 5.2 | up | Zn dependent hydrolase | Pden_1113 | PDEN_RS05515 |
| cds-WP_081465060.1 | *-* | 4.66576E-19 | 18 | 1.3455E-20 | 5.2 | up | ompA family protein | Pden_2459 | PDEN_RS25615 |
| cds-WP_011751202.1 | *-* | 4.32877E-16 | 15 | 1.58621E-17 | 5.1 | up | coA ester lyase | Pden_4110 | PDEN_RS20485 |
| cds-WP_011748724.1 | *tssC* | 6.37613E-21 | 20 | 1.54841E-22 | 5.0 | up | tssC CDS (part of type VI secretion system) | Pden_2444 | PDEN_RS12150 |
| cds-WP_011750349.1 | *-* | 1.99488E-65 | 65 | 8.65081E-68 | 5.0 | up | acyl-CoA/Acyl-ACP-DH | Pden_4113 | PDEN_RS20500 |
| cds-PDEN_RS26020 | *-* | 5.51764E-06 | 5 | 8.63777E-07 | 5.0 | up | ribonucleoside triphosphate reductase CDS | Pden_1345 | PDEN_RS26020 |
| cds-WP_011749103.1 | *fdxH* | 2.50497E-23 | 23 | 4.9969E-25 | 4.9 | up | fdxH CDS (formate dh) | Pden_2828 | PDEN_RS14080 |
| cds-WP_041529775.1 | *-* | 1.7325E-150 | 150 | 2.2538E-153 | 4.9 | up | L-malyl-CoA/beta-methylmalyl-CoA lyase CDS = mcl-1 | Pden_0799 | PDEN_RS03970 |
| cds-WP_011750348.1 | *-* | 5.14592E-55 | 54 | 2.67784E-57 | 4.8 | up | class I SAM-dependent methyltransferase CDS | Pden_4117 | PDEN_RS20515 |
| cds-WP_011751201.1 | *-* | 6.59275E-12 | 11 | 4.3742E-13 | 4.7 | up | acetoin dehydrogenase dihydrolipoyllysine-residue acetyltransferase subunit CDS | Pden_4983 | PDEN_RS24780 |
| cds-WP_011748644.1 | *pqqC* | 6.64845E-07 | 6 | 9.09621E-08 | 4.7 | up | pyrroloquinoline-quinone synthase PqqC | Pden_2361 | PDEN_RS11725 |
| cds-WP_011749179.1 | *-* | 1.03188E-08 | 8 | 1.04261E-09 | 4.7 | up | enoyl-CoA hydratase/isomerase family protein CDS | Pden_2905 | PDEN_RS14470 |
| cds-WP_011750428.1 | *-* | 1.94702E-12 | 12 | 1.20739E-13 | 4.6 | up | hypothetical protein CDS | Pden_4199 | PDEN_RS20920 |
| cds-WP_049792275.1 | *-* | 4.54269E-13 | 12 | 2.59048E-14 | 4.5 | up | ABC transporter substrate-binding protein CDS | Pden_0026 | PDEN_RS00135 |
| cds-WP_011748651.1 | *-* | 4.6819E-20 | 19 | 1.23849E-21 | 4.5 | up | DUF779 protein | Pden_2368 | PDEN_RS11765 |
| cds-WP_011746378.1 | *-* | 2.3877E-13 | 13 | 1.30464E-14 | 4.4 | up | hypothetical protein CDS | Pden_0028 | PDEN_RS00145 |
| cds-WP_011748177.1 | *-* | 3.11737E-07 | 7 | 4.00148E-08 | 4.4 | up | DUF2147 domain-containing protein CDS | Pden_1885 | PDEN_RS09315 |
| cds-WP_011748723.1 | *tssB* | 1.23541E-08 | 8 | 1.26702E-09 | 4.4 | up | type VI secretion system contractile sheath small subunit | Pden_2443 | PDEN_RS12145 |
| cds-WP_011748725.1 | *-* | 7.58753E-13 | 12 | 4.54067E-14 | 4.4 | up | type VI secretion system tube protein Hcp CDS | Pden_2445 | PDEN_RS12155 |
| cds-WP_011750780.1 | *-* | 1.16669E-05 | 5 | 1.94027E-06 | 4.4 | up | 3'-5' exonuclease CDS | Pden_4556 | PDEN_RS22695 |
| cds-WP_049792433.1 | *-* | 9.07072E-12 | 11 | 6.13631E-13 | 4.4 | up | hydantoinase/oxoprolinase family protein CDS | Pden_4408 | PDEN_RS21950 |
| cds-WP_024843519.1 | *pqqA* | 1.79816E-07 | 7 | 2.20676E-08 | 4.3 | up | pqqA CDS (pyroloquinone synthesis) | - | PDEN_RS11735 |
| cds-WP_011748645.1 | *pqqB* | 7.90747E-09 | 8 | 7.86975E-10 | 4.2 | up | pyrroloquinoline quinone biosynthesis protein PqqB | Pden_2362 | PDEN_RS11730 |
| cds-WP_011748641.1 | *-* | 2.18742E-05 | 5 | 3.81803E-06 | 4.2 | up | quinoprotein dehydrogenase-associated SoxYZ-likecarrier CDS | Pden_2358 | PDEN_RS11710 |
| cds-WP_041530319.1 | *-* | 1.33483E-30 | 30 | 1.59184E-32 | 4.2 | up | AMP-binding protein CDS | Pden_2909 | PDEN_RS14490 |
| cds-WP_041530599.1 | *-* | 3.16588E-44 | 43 | 1.99069E-46 | 4.1 | up | MaoC family dehydratase CDS | Pden_3661 | PDEN_RS18165 |
| cds-WP_011746377.1 | *-* | 1.5629E-15 | 15 | 6.40476E-17 | 4.1 | up | YVTN family beta-propeller repeat protein CDS | Pden_0027 | PDEN_RS00140 |
| cds-WP_011746655.1 | *-* | 1.8323E-32 | 32 | 2.06591E-34 | 4.1 | up | tripartite tricarboxylate transporter substrate binding protein CDS | Pden_0308 | PDEN_RS01495 |
| cds-WP_198140528.1 | *fdnG* | 6.11179E-72 | 71 | 2.25283E-74 | 4.0 | up | fdnG CDS (formate dh) | Pden_2829 | PDEN_RS14085 |
| cds-WP_011748650.1 | *adhP* | 5.07158E-26 | 25 | 8.35732E-28 | 3.9 | up | alcohol-DH | Pden_2367 | PDEN_RS11760 |
| cds-WP_011748649.1 | *-* | 6.78671E-27 | 26 | 1.00064E-28 | 3.9 | up | aldehyde dehydrogenase family protein CDS | Pden_2366 | PDEN_RS11755 |
| cds-WP_011749181.1 | *-* | 2.93138E-22 | 22 | 6.48311E-24 | 3.9 | up | acetyl-CoA C-acyltransferase CDS | Pden_2907 | PDEN_RS14480 |
| cds-WP_011749960.1 | *-* | 9.56337E-43 | 42 | 6.63547E-45 | 3.8 | up | LuxR family transcriptional regulator CDS | Pden_3724 | PDEN_RS18480 |
| cds-WP_011746908.1 | *mcl-2* | 1.01917E-48 | 48 | 5.9665E-51 | 3.7 | up | CoA ester lyase CDS | Pden_0563 | PDEN_RS02805 |
| cds-WP_011751217.1 | *-* | 1.01323E-06 | 6 | 1.43021E-07 | 3.7 | up | branched-chain amino acid ABC transporter permease CDS | Pden_4999 | PDEN_RS24860 |
| cds-WP_155984334.1 | *-* | 7.53288E-12 | 11 | 5.0633E-13 | 3.7 | up | hypothetical protein CDS | - | PDEN_RS26985 |
| cds-WP_011750691.1 | *-* | 1.80466E-20 | 20 | 4.61729E-22 | 3.6 | up | hypothetical protein CDS | Pden_4466 | PDEN_RS22255 |
| cds-WP_011750942.1 | *-* | 8.5441E-24 | 23 | 1.61174E-25 | 3.6 | up | nitrate reductase cytochrome c-type subunit CDS | Pden_4722 | PDEN_RS23505 |
| cds-WP_011747066.1 | *-* | 1.75818E-32 | 32 | 1.94422E-34 | 3.5 | up | patatin-like phospholipase family protein CDS | Pden_0721 | PDEN_RS03585 |
| cds-WP_011746869.1 | *-* | 1.01414E-10 | 10 | 7.78413E-12 | 3.5 | up | purine-nucleoside phosphorylase CDS | Pden_0524 | PDEN_RS02610 |
| cds-WP_011750427.1 | *-* | 7.30621E-06 | 5 | 1.16912E-06 | 3.5 | up | RiPP maturation radical SAM C-methyltransferase CDS | Pden_4198 | PDEN_RS20915 |
| cds-WP_011746654.1 | *-* | 9.80012E-09 | 8 | 9.85962E-10 | 3.5 | up | tripartite tricarboxylate transporter TctB family protein CDS | Pden_0307 | PDEN_RS01490 |
| cds-WP_011748720.1 | *tssH* | 1.43033E-10 | 10 | 1.11958E-11 | 3.5 | up | tssH CDS (type IV secretion system ATPase) | Pden_2440 | PDEN_RS12130 |
| cds-WP_011746911.1 | *-* | 7.95539E-69 | 68 | 3.10488E-71 | 3.4 | up | MaoC family dehydratase CDS | Pden_0566 | PDEN_RS02820 |
| cds-WP_011749579.1 | *-* | 4.64028E-11 | 10 | 3.43091E-12 | 3.4 | up | hypothetical protein CDS | Pden_3318 | PDEN_RS16475 |
| cds-WP_011748365.1 | *-* | 3.04568E-42 | 42 | 2.17926E-44 | 3.4 | up | hypothetical protein CDS | Pden_2078 | PDEN_RS10325 |
| cds-WP_011749102.1 | *-* | 2.5488E-13 | 13 | 1.40924E-14 | 3.4 | up | formate dehydrogenase subunit gamma CDS | Pden_2827 | PDEN_RS14075 |
| cds-WP_011746653.1 | *-* | 1.13916E-21 | 21 | 2.64288E-23 | 3.4 | up | tripartite tricarboxylate transporter permease CDS | Pden_0306 | PDEN_RS01485 |
| cds-WP_011749182.1 | *-* | 6.91107E-11 | 10 | 5.15483E-12 | 3.4 | up | SDR family NAD(P)-dependent oxidoreductase CDS | Pden_2908 | PDEN_RS14485 |
| cds-WP_011750943.1 | *-* | 1.13687E-41 | 41 | 8.6276E-44 | 3.4 | up | cytochrome c3 family protein CDS | Pden_4723 | PDEN_RS23510 |
| cds-WP_011750777.1 | *-* | 1.54807E-11 | 11 | 1.09761E-12 | 3.3 | up | hypothetical protein CDS | Pden_4553 | PDEN_RS22680 |
| cds-WP_011749876.1 | *-* | 1.72693E-06 | 6 | 2.52001E-07 | 3.3 | up | glutathione S-transferase CDS | Pden_3640 | PDEN_RS18050 |
| cds-WP_011749180.1 | *-* | 4.30404E-16 | 15 | 1.56782E-17 | 3.3 | up | acyl-CoA dehydrogenase family protein CDS | Pden_2906 | PDEN_RS14475 |
| cds-WP_011751197.1 | *-* | 1.07524E-09 | 9 | 9.62865E-11 | 3.3 | up | sigma-54-dependent Fis family transcriptional regulator CDS | Pden_4979 | PDEN_RS24760 |
| cds-WP_011746754.1 | *-* | 4.65539E-07 | 6 | 6.1574E-08 | 3.3 | up | glutathione S-transferase family protein CDS | Pden_0407 | PDEN_RS02020 |
| cds-WP_011748943.1 | *-* | 9.8708E-26 | 25 | 1.66939E-27 | 3.2 | up | acetyl-CoA C-acyltransferase family protein CDS | Pden_2663 | PDEN_RS13255 |
| cds-WP_011748761.1 | *-* | 3.21781E-06 | 5 | 4.87694E-07 | 3.2 | up | VWA domain-containing protein CDS | Pden_2481 | PDEN_RS12330 |
| cds-WP_011750941.1 | *napA* | 3.08196E-56 | 56 | 1.53697E-58 | 3.2 | up | nitrate reductase | Pden_4721 | PDEN_RS23500 |
| cds-WP_011750776.1 | *-* | 1.51575E-41 | 41 | 1.18315E-43 | 3.2 | up | cation acetate symporter CDS | Pden_4552 | PDEN_RS22675 |
| cds-WP_011750775.1 | *-* | 7.95356E-16 | 15 | 3.13865E-17 | 3.2 | up | DUF4212 domain-containing protein CDS | Pden_4551 | PDEN_RS22670 |
| cds-WP_011749819.1 | *-* | 1.9589E-05 | 5 | 3.38517E-06 | 3.2 | up | helix-turn-helix transcriptional regulator CDS | Pden_3582 | PDEN_RS17750 |
| cds-WP_011749580.1 | *-* | 9.75971E-11 | 10 | 7.46512E-12 | 3.2 | up | hypothetical protein CDS | Pden_3319 | PDEN_RS16480 |
| cds-WP_011747141.1 | *-* | 1.29467E-05 | 5 | 2.16995E-06 | 3.2 | up | ABC transporter permease CDS | Pden_0801 | PDEN_RS03985 |
| cds-WP_011750768.1 | *-* | 9.64117E-07 | 6 | 1.3567E-07 | 3.2 | up | TRAP transporter small permease subunit CDS | Pden_4544 | PDEN_RS22635 |
| cds-WP_041530739.1 | *-* | 1.80032E-05 | 5 | 3.09551E-06 | 3.2 | up | branched-chain amino acid ABC transporter permease CDS | Pden_5000 | PDEN_RS24865 |
| cds-WP_011746909.1 | *-* | 1.03606E-45 | 45 | 6.29002E-48 | 3.2 | up | NnrU family protein CDS | Pden_0564 | PDEN_RS02810 |
| cds-WP_011749262.1 | *-* | 5.60611E-13 | 12 | 3.24551E-14 | 3.1 | up | response regulator transcription factor CDS | Pden_2990 | PDEN_RS14925 |
| cds-WP_011749695.1 | *-* | 7.58016E-09 | 8 | 7.52756E-10 | 3.1 | up | efflux RND transporter periplasmic adaptor subunit CDS | Pden_3447 | PDEN_RS17075 |
| cds-WP_011750423.1 | *-* | 1.23293E-17 | 17 | 3.90302E-19 | 3.1 | up | SARP family transcriptional regulator CDS | Pden_4194 | PDEN_RS20895 |
| cds-WP_011749875.1 | *-* | 2.94793E-08 | 8 | 3.21511E-09 | 3.1 | up | ATP-grasp domain-containing protein CDS | Pden_3639 | PDEN_RS18045 |
| cds-WP_011749887.1 | *aqpZ* | 1.05782E-11 | 11 | 7.40844E-13 | 3.1 | up | aqpZ CDS (aquaporin) | Pden_3651 | PDEN_RS18105 |
| cds-WP_011748964.1 | *-* | 5.80276E-42 | 41 | 4.27784E-44 | 3.1 | up | DUF2853 family protein CDS | Pden_2684 | PDEN_RS13360 |
| cds-WP_011749581.1 | *-* | 3.95201E-24 | 23 | 7.19794E-26 | 3.1 | up | VWA domain-containing protein CDS | Pden_3320 | PDEN_RS16485 |
| cds-WP_011746380.1 | *-* | 6.05177E-05 | 4 | 1.14947E-05 | 3.0 | up | ABC transporter permease CDS | Pden_0030 | PDEN_RS00155 |
| cds-WP_011749577.1 | *-* | 3.20521E-08 | 7 | 3.51656E-09 | 3.0 | up | hypothetical protein CDS | Pden_3316 | PDEN_RS16465 |
| cds-WP_074810587.1 | *-* | 1.11666E-25 | 25 | 1.91275E-27 | 3.0 | up | YdcH family protein CDS (methyltransferase) | Pden_2029 | PDEN_RS10090 |
| cds-WP_011749106.1 | *-* | 2.39457E-17 | 17 | 7.68422E-19 | 3.0 | up | hybrid-cluster NAD(P)-dependent oxidoreductase CDS | Pden_2831 | PDEN_RS14095 |
| cds-WP_011746935.1 | *-* | 4.84807E-15 | 14 | 2.08675E-16 | 3.0 | up | outer membrane protein transport protein CDS | Pden_0590 | PDEN_RS02940 |
| cds-WP_011750977.1 | *-* | 1.56669E-22 | 22 | 3.36301E-24 | 3.0 | up | 3-methyl-2-oxobutanoate dehydrogenase (2-methylpropanoyl-transferring) subunit alpha CDS | Pden_4757 | PDEN_RS23680 |
| cds-WP_011749391.1 | *-* | 1.08733E-14 | 14 | 4.97455E-16 | 3.0 | up | recombinase family protein CDS | Pden_3123 | PDEN_RS15550 |
| cds-WP_011750769.1 | *-* | 5.31956E-16 | 15 | 1.98388E-17 | 3.0 | up | TRAP transporter large permease subunit CDS | Pden_4545 | PDEN_RS22640 |
| cds-WP_011748715.1 | *-* | 4.62323E-05 | 4 | 8.55078E-06 | 3.0 | up | hypothetical protein CDS | Pden_2435 | PDEN_RS12105 |
| cds-WP_011750436.1 | *-* | 6.0014E-08 | 7 | 6.88365E-09 | 3.0 | up | polyhydroxyalkanoic acid synthase CDS | Pden_4207 | PDEN_RS20960 |
| cds-WP_011749263.1 | *-* | 1.51237E-07 | 7 | 1.83308E-08 | 2.9 | up | HAMP domain-containing protein CDS | Pden_2991 | PDEN_RS14930 |
| cds-WP_011749578.1 | *-* | 3.98472E-09 | 8 | 3.87067E-10 | 2.9 | up | hypothetical protein CDS | Pden_3317 | PDEN_RS16470 |
| cds-WP_011749835.1 | *-* | 5.12081E-10 | 9 | 4.38578E-11 | 2.9 | up | hypothetical protein CDS | Pden_3599 | PDEN_RS27125 |
| cds-WP_011749582.1 | *-* | 5.81499E-08 | 7 | 6.6194E-09 | 2.9 | up | hypothetical protein CDS | Pden_3321 | PDEN_RS16490 |
| cds-WP_011748551.1 | *mscL* | 1.59977E-31 | 31 | 1.8731E-33 | 2.9 | up | mscL CDS (mechanosensitive channel protein) | Pden_2265 | PDEN_RS11260 |
| cds-WP_011751071.1 | *-* | 3.42961E-13 | 12 | 1.93343E-14 | 2.9 | up | caspase family protein | Pden_4852 | PDEN_RS24150 |
| cds-WP_011746365.1 | *gfa* | 4.61173E-07 | 6 | 6.08965E-08 | 2.9 | up | gfa CDS (glutathion synthase) | Pden_0015 | PDEN_RS00080 |
| cds-WP_011749647.1 | *-* | 5.68427E-05 | 4 | 1.07843E-05 | 2.9 | up | twin-arginine translocation pathway signal CDS | Pden_3398 | PDEN_RS16825 |
| cds-WP_011747579.1 | *-* | 3.74546E-09 | 8 | 3.63014E-10 | 2.9 | up | metal ABC transporter permease CDS | Pden_1256 | PDEN_RS06200 |
| cds-WP_011746795.1 | *-* | 5.42676E-13 | 12 | 3.12992E-14 | 2.9 | up | hypothetical protein CDS | Pden_0448 | PDEN_RS02230 |
| cds-WP_011749944.1 | *-* | 1.00326E-09 | 9 | 8.89709E-11 | 2.9 | up | DUF1330 domain-containing protein CDS | Pden_3708 | PDEN_RS18410 |
| cds-WP_011749576.1 | *-* | 9.90065E-13 | 12 | 5.98933E-14 | 2.8 | up | hypothetical protein CDS | Pden_3315 | PDEN_RS27080 |
| cds-WP_011746910.1 | *-* | 1.93264E-30 | 30 | 2.38856E-32 | 2.8 | up | DUF1737 domain-containing protein CDS | Pden_0565 | PDEN_RS02815 |
| cds-WP_011748722.1 | *-* | 4.96211E-05 | 4 | 9.26361E-06 | 2.8 | up | type VI secretion system ImpA family N-terminal domain-containing protein CDS | Pden_2442 | PDEN_RS12140 |
| cds-WP_011751215.1 | *-* | 5.21718E-05 | 4 | 9.83029E-06 | 2.8 | up | ABC transporter ATP-binding protein CDS | Pden_4997 | PDEN_RS24850 |
| cds-WP_011749873.1 | *-* | 6.00185E-13 | 12 | 3.50064E-14 | 2.8 | up | methylcrotonoyl-CoA carboxylase CDS | Pden_3637 | PDEN_RS18035 |
| cds-WP_049792328.1 | *-* | 3.82534E-06 | 5 | 5.88896E-07 | 2.8 | up | sigma-54-dependent Fis family transcriptional regulator CDS | Pden_2365 | PDEN_RS11750 |
| cds-WP_011748735.1 | *tssI* | 3.57935E-09 | 8 | 3.4381E-10 | 2.8 | up | type VI secretion system tip protein VgrG | Pden_2455 | PDEN_RS12205 |
| cds-WP_011748395.1 | *-* | 3.31588E-07 | 6 | 4.27786E-08 | 2.8 | up | Crp/Fnr family transcriptional regulator CDS | Pden_2108 | PDEN_RS10470 |
| cds-WP_011750754.1 | *-* | 5.86663E-08 | 7 | 6.70363E-09 | 2.8 | up | sodium:solute symporter family protein CDS | Pden_4529 | PDEN_RS22575 |
| cds-WP_011746794.1 | *-* | 3.60495E-20 | 19 | 9.37976E-22 | 2.8 | up | hypothetical protein CDS | Pden_0447 | PDEN_RS02225 |
| cds-WP_011750770.1 | *dctP* | 1.08124E-18 | 18 | 3.16493E-20 | 2.8 | up | dctP CDS | Pden_4546 | PDEN_RS22645 |
| cds-WP_164901615.1 | *-* | 6.64984E-08 | 7 | 7.65626E-09 | 2.8 | up | hypothetical protein CDS | - | PDEN_RS27345 |
| cds-WP_011750560.1 | *-* | 6.2623E-13 | 12 | 3.66614E-14 | 2.7 | up | APC family permease CDS | Pden_4334 | PDEN_RS21580 |
| cds-WP_011750863.1 | *-* | 1.06349E-12 | 12 | 6.47964E-14 | 2.7 | up | tyrosinase family protein CDS | Pden_4643 | PDEN_RS23115 |
| cds-WP_011749264.1 | *-* | 3.38897E-08 | 7 | 3.74756E-09 | 2.7 | up | protein MoxZ CDS | Pden_2992 | PDEN_RS14935 |
| cds-WP_049792297.1 | *-* | 8.8447E-06 | 5 | 1.43832E-06 | 2.7 | up | DNA/RNA non-specific endonuclease CDS | Pden_0949 | PDEN_RS04715 |
| cds-WP_011750773.1 | *-* | 1.26297E-13 | 13 | 6.54488E-15 | 2.6 | up | iron-containing alcohol dehydrogenase CDS | Pden_4549 | PDEN_RS22660 |
| cds-WP_011749107.1 | *-* | 1.78847E-20 | 20 | 4.49832E-22 | 2.6 | up | aromatic ring-hydroxylating dioxygenase subunit alpha CDS | Pden_2832 | PDEN_RS14100 |
| cds-WP_011746953.1 | *-* | 2.64213E-19 | 19 | 7.3329E-21 | 2.6 | up | peptide ABC transporter substrate-binding protein CDS | Pden_0608 | PDEN_RS03025 |
| cds-WP_011750740.1 | *-* | 4.10865E-17 | 16 | 1.37193E-18 | 2.6 | up | VOC family protein CDS | Pden_4515 | PDEN_RS22505 |
| cds-WP_011750633.1 | *-* | 2.99821E-05 | 5 | 5.35674E-06 | 2.6 | up | allantoin permease CDS | Pden_4407 | PDEN_RS21945 |
| cds-WP_011746952.1 | *-* | 6.55491E-16 | 15 | 2.51565E-17 | 2.6 | up | ABC transporter permease CDS | Pden_0607 | PDEN_RS03020 |
| cds-WP_011750487.1 | *xdhB* | 1.1606E-13 | 13 | 5.98923E-15 | 2.6 | up | xanthine dehydrogenase molybdopterin binding subunit | Pden_4258 | PDEN_RS21210 |
| cds-WP_011750418.1 | *ugpA* | 4.02813E-19 | 18 | 1.13542E-20 | 2.6 | up | sn-glycerol-3-phosphate ABC transporter permeaseUgpA | Pden_4189 | PDEN_RS20870 |
| cds-WP_011750978.1 | *-* | 2.46167E-09 | 9 | 2.30582E-10 | 2.6 | up | alpha-ketoacid dehydrogenase subunit beta CDS | Pden_4758 | PDEN_RS23685 |
| cds-WP_011746738.1 | *-* | 4.50678E-05 | 4 | 8.2963E-06 | 2.6 | up | hypothetical protein CDS | Pden_0391 | PDEN_RS01930 |
| cds-WP_011746796.1 | *-* | 1.38646E-13 | 13 | 7.27501E-15 | 2.6 | up | DNA polymerase III subunit epsilon CDS | Pden_0449 | PDEN_RS25930 |
| cds-WP_011750417.1 | *ugpE* | 1.59096E-18 | 18 | 4.69147E-20 | 2.6 | up | sn-glycerol-3-phosphate ABC transporter permeaseUgpE | Pden_4188 | PDEN_RS20865 |
| cds-WP_011746954.1 | *-* | 8.56217E-15 | 14 | 3.84295E-16 | 2.6 | up | ABC transporter ATP-binding protein CDS | Pden_0609 | PDEN_RS03030 |
| cds-WP_128492982.1 | *-* | 5.46752E-08 | 7 | 6.20016E-09 | 2.6 | up | hypothetical protein CDS | - | PDEN_RS04720 |
| cds-WP_011747391.1 | *-* | 4.88201E-09 | 8 | 4.79521E-10 | 2.6 | up | flavin reductase family protein CDS | Pden_1062 | PDEN_RS05275 |
| cds-WP_011750940.1 | *-* | 4.0844E-11 | 10 | 2.99334E-12 | 2.6 | up | chaperone NapD CDS | Pden_4720 | PDEN_RS23495 |
| cds-WP_011750059.1 | *-* | 1.01888E-11 | 11 | 7.11358E-13 | 2.6 | up | CoA-binding protein CDS | Pden_3824 | PDEN_RS19025 |
| cds-WP_011746951.1 | *-* | 7.02985E-07 | 6 | 9.63327E-08 | 2.6 | up | ABC transporter permease CDS | Pden_0606 | PDEN_RS03015 |
| cds-WP_011746870.1 | *-* | 5.34176E-17 | 16 | 1.80684E-18 | 2.5 | up | ABC transporter permease CDS | Pden_0525 | PDEN_RS02615 |
| cds-WP_041529929.1 | *-* | 7.64945E-10 | 9 | 6.71732E-11 | 2.5 | up | hypothetical protein CDS | - | PDEN_RS10215 |
| cds-WP_011749574.1 | *-* | 9.43538E-11 | 10 | 7.18087E-12 | 2.5 | up | molybdopterin-dependent oxidoreductase CDS | Pden_3313 | PDEN_RS16450 |
| cds-WP_011747752.1 | *bioB* | 2.76344E-15 | 15 | 1.15643E-16 | 2.5 | up | bioB CDS (part of biotin synthesis/import cluster) | Pden_1433 | PDEN_RS07085 |
| cds-WP_198140556.1 | *-* | 5.14456E-05 | 4 | 9.67116E-06 | 2.5 | up | hydantoinase/oxoprolinase family protein CDS | Pden_4263 | PDEN_RS21235 |
| cds-WP_011751198.1 | *-* | 2.51754E-08 | 8 | 2.71296E-09 | 2.5 | up | 2,3-butanediol dehydrogenase CDS | Pden_4980 | PDEN_RS24765 |
| cds-WP_011746553.1 | *-* | 1.33024E-05 | 5 | 2.23821E-06 | 2.5 | up | SDR family oxidoreductase CDS | Pden_0206 | PDEN_RS00990 |
| cds-WP_011748717.1 | *-* | 2.12119E-05 | 5 | 3.69783E-06 | 2.5 | up | DUF4150 domain-containing protein CDS | Pden_2437 | PDEN_RS25610 |
| cds-WP_011748261.1 | *-* | 5.64885E-05 | 4 | 1.07008E-05 | 2.5 | up | recombinase family protein CDS | Pden_1972 | PDEN_RS26105 |
| cds-WP_011750702.1 | *-* | 7.23386E-10 | 9 | 6.28963E-11 | 2.5 | up | CBS domain-containing protein CDS | Pden_4477 | PDEN_RS22310 |
| cds-WP_011747298.1 | *-* | 1.31044E-39 | 39 | 1.10813E-41 | 2.5 | up | hypothetical protein CDS | Pden_0959 | PDEN_RS04770 |
| cds-WP_041530327.1 | *-* | 1.54435E-05 | 5 | 2.63002E-06 | 2.5 | up | hypothetical protein CDS | - | PDEN_RS15600 |
| cds-WP_011747675.1 | *-* | 1.78372E-07 | 7 | 2.18517E-08 | 2.4 | up | methionine synthase CDS | Pden_1355 | PDEN_RS06690 |
| cds-WP_011748716.1 | *-* | 6.6643E-12 | 11 | 4.43612E-13 | 2.4 | up | hypothetical protein CDS | Pden_2436 | PDEN_RS12110 |
| cds-WP_011748294.1 | *-* | 1.52114E-08 | 8 | 1.56996E-09 | 2.4 | up | acyl-CoA synthetase CDS | Pden_2005 | PDEN_RS09970 |
| cds-WP_011748341.1 | *-* | 2.10131E-07 | 7 | 2.60614E-08 | 2.4 | up | hypothetical protein CDS | Pden_2053 | PDEN_RS10205 |
| cds-WP_041530076.1 | *-* | 8.93522E-22 | 21 | 2.03425E-23 | 2.4 | up | ABC transporter permease CDS | Pden_0526 | PDEN_RS02620 |
| cds-WP_011748015.1 | *-* | 3.9757E-08 | 7 | 4.46534E-09 | 2.4 | up | malonyl-CoA decarboxylase CDS | Pden_1720 | PDEN_RS08455 |
| cds-WP_011750419.1 | *ugpB* | 1.17588E-26 | 26 | 1.81022E-28 | 2.4 | up | ugpB CDS (glycerol-3-p permease) | Pden_4190 | PDEN_RS20875 |
| cds-WP_011747674.1 | *-* | 3.06796E-07 | 7 | 3.93141E-08 | 2.4 | up | DUF1852 domain-containing protein CDS | Pden_1354 | PDEN_RS06685 |
| cds-WP_128492935.1 | *-* | 2.92407E-12 | 12 | 1.864E-13 | 2.4 | up | hypothetical protein CDS | Pden_1356 | PDEN_RS26870 |
| cds-WP_011747140.1 | *-* | 4.98541E-07 | 6 | 6.6155E-08 | 2.4 | up | ABC transporter ATP-binding protein CDS | Pden_0800 | PDEN_RS03980 |
| cds-WP_011750862.1 | *-* | 3.99193E-18 | 17 | 1.21177E-19 | 2.4 | up | hypothetical protein CDS | Pden_4642 | PDEN_RS23110 |
| cds-WP_011751042.1 | *-* | 8.26721E-07 | 6 | 1.14992E-07 | 2.4 | up | SDR family oxidoreductase CDS | Pden_4822 | PDEN_RS24005 |
| cds-WP_011750489.1 | *guaD* | 5.69731E-12 | 11 | 3.74303E-13 | 2.4 | up | guaD CDS | Pden_4260 | PDEN_RS21220 |
| cds-WP_011747600.1 | *-* | 5.66681E-23 | 22 | 1.16727E-24 | 2.4 | up | purine permease CDS | Pden_1277 | PDEN_RS06305 |
| cds-WP_011750082.1 | *-* | 2.45053E-12 | 12 | 1.54619E-13 | 2.4 | up | site-specific integrase CDS | Pden_3849 | PDEN_RS19160 |
| cds-WP_011750861.1 | *-* | 1.49889E-10 | 10 | 1.17649E-11 | 2.4 | up | hypothetical protein CDS | Pden_4641 | PDEN_RS23105 |
| cds-WP_011751214.1 | *-* | 2.19471E-08 | 8 | 2.32224E-09 | 2.4 | up | acetate--CoA ligase family protein CDS | Pden_4996 | PDEN_RS24845 |
| cds-WP_011749717.1 | *-* | 8.16135E-16 | 15 | 3.25605E-17 | 2.3 | up | hypothetical protein CDS | Pden_3470 | PDEN_RS17190 |
| cds-WP_011750980.1 | *lpdA* | 3.72321E-06 | 5 | 5.7156E-07 | 2.3 | up | lpdA CDS | Pden_4760 | PDEN_RS23695 |
| cds-WP_011748944.1 | *-* | 3.69179E-10 | 9 | 3.09784E-11 | 2.3 | up | LysR family transcriptional regulator CDS | Pden_2664 | PDEN_RS13260 |
| cds-WP_011750771.1 | *-* | 9.48495E-12 | 11 | 6.49879E-13 | 2.3 | up | glutamine synthetase CDS | Pden_4547 | PDEN_RS22650 |
| cds-WP_011749534.1 | *-* | 1.38238E-09 | 9 | 1.2439E-10 | 2.3 | up | LysR family transcriptional regulator CDS | Pden_3271 | PDEN_RS16245 |
| cds-WP_011750772.1 | *-* | 6.89098E-08 | 7 | 7.97871E-09 | 2.3 | up | aldehyde dehydrogenase family protein CDS | Pden_4548 | PDEN_RS22655 |
| cds-WP_011746947.1 | *-* | 4.88398E-14 | 13 | 2.35092E-15 | 2.3 | up | hypothetical protein CDS | Pden_0602 | PDEN_RS02995 |
| cds-WP_041530454.1 | *-* | 3.6689E-08 | 7 | 4.10484E-09 | 2.3 | up | glycosyltransferase family 2 protein CDS | Pden_4162 | PDEN_RS20735 |
| cds-WP_011747779.1 | *-* | 8.09818E-05 | 4 | 1.56275E-05 | 2.3 | up | GNAT family N-acetyltransferase CDS | Pden_1461 | PDEN_RS07200 |
| cds-WP_011748633.1 | *-* | 5.56367E-05 | 4 | 1.05193E-05 | 2.3 | up | hypothetical protein CDS | Pden_2350 | PDEN_RS11670 |
| cds-WP_011751039.1 | *-* | 9.28236E-10 | 9 | 8.21162E-11 | 2.3 | up | acetyl-CoA acetyltransferase CDS | Pden_4819 | PDEN_RS23990 |
| cds-WP_011748185.1 | *msrB* | 1.28044E-05 | 5 | 2.14332E-06 | 2.3 | up | msrB CDS | Pden_1893 | PDEN_RS09355 |
| cds-WP_128493090.1 | *-* | 2.5118E-06 | 6 | 3.74156E-07 | 2.3 | up | hypothetical protein CDS | Pden_3848 | PDEN_RS19155 |
| cds-WP_011748543.1 | *-* | 9.43538E-11 | 10 | 7.16121E-12 | 2.2 | up | NAD-dependent succinate-semialdehyde dehydrogenase CDS | Pden_2257 | PDEN_RS11220 |
| cds-WP_011750493.1 | *-* | 7.28019E-23 | 22 | 1.51539E-24 | 2.2 | up | sigma-54-dependent Fis family transcriptional regulator CDS | Pden_4264 | PDEN_RS21240 |
| cds-WP_011749132.1 | *-* | 2.86647E-10 | 10 | 2.35558E-11 | 2.2 | up | formate dehydrogenase beta subunit CDS | Pden_2857 | PDEN_RS14225 |
| cds-WP_041529877.1 | *-* | 2.15456E-10 | 10 | 1.73318E-11 | 2.2 | up | LacI family DNA-binding transcriptional regulator CDS | Pden_1685 | PDEN_RS08280 |
| cds-WP_011749587.1 | *-* | 1.56436E-05 | 5 | 2.66945E-06 | 2.2 | up | recombinase family protein CDS | Pden_0106 | PDEN_RS00535 |
| cds-WP_011749390.1 | *-* | 1.1514E-08 | 8 | 1.17337E-09 | 2.2 | up | tyrosine-type recombinase/integrase CDS | Pden_3122 | PDEN_RS15545 |
| cds-WP_011750707.1 | *mmsB* | 1.76933E-11 | 11 | 1.26216E-12 | 2.2 | up | 3-hydroxyisobutyrate dehydrogenase | Pden_4482 | PDEN_RS22335 |
| cds-WP_011748636.1 | *-* | 3.39695E-08 | 7 | 3.76375E-09 | 2.2 | up | FIST C-terminal domain-containing protein CDS | Pden_2353 | PDEN_RS11685 |
| cds-WP_074810797.1 | *-* | 5.71194E-13 | 12 | 3.31917E-14 | 2.2 | up | hypothetical protein CDS | Pden_0973 | PDEN_RS04850 |
| cds-WP_128493044.1 | *-* | 2.07601E-05 | 5 | 3.60555E-06 | 2.2 | up | hypothetical protein CDS | Pden_3322 | PDEN_RS16495 |
| cds-WP_011747100.1 | *-* | 4.53575E-10 | 9 | 3.84536E-11 | 2.2 | up | hypothetical protein CDS | Pden_0757 | PDEN_RS03760 |
| cds-WP_157137424.1 | *-* | 1.35322E-20 | 20 | 3.37425E-22 | 2.2 | up | hypothetical protein CDS |  | PDEN_RS26815 |
| cds-WP_011747881.1 | *-* | 1.44805E-15 | 15 | 5.90274E-17 | 2.2 | up | TRAP transporter substrate-binding protein CDS | Pden_1563 | PDEN_RS07680 |
| cds-WP_011747751.1 | *-* | 8.98662E-05 | 4 | 1.75757E-05 | 2.2 | up | biotin transporter BioY CDS | Pden_1432 | PDEN_RS07080 |
| cds-WP_157137429.1 | *-* | 8.70516E-06 | 5 | 1.41374E-06 | 2.2 | up | hypothetical protein CDS |  | PDEN_RS26875 |
| cds-WP_011750416.1 | *-* | 1.88572E-09 | 9 | 1.73771E-10 | 2.2 | up | sn-glycerol-3-phosphate import ATP-binding protein UgpC CDS | Pden_4187 | PDEN_RS20860 |
| cds-WP_011748634.1 | *-* | 4.83278E-06 | 5 | 7.54467E-07 | 2.2 | up | hypothetical protein CDS | Pden_2351 | PDEN_RS11675 |
| cds-WP_011750486.1 | *xdhA* | 3.23361E-08 | 7 | 3.55473E-09 | 2.2 | up | xanthine dehydrogenase small subunit | Pden_4257 | PDEN_RS21205 |
| cds-WP_011749823.1 | *-* | 8.16135E-16 | 15 | 3.25456E-17 | 2.2 | up | transglutaminase domain-containing protein CDS | Pden_3586 | PDEN_RS17770 |
| cds-WP_011751037.1 | *-* | 9.94574E-12 | 11 | 6.92234E-13 | 2.2 | up | TRAP transporter substrate-binding protein CDS | Pden_4817 | PDEN_RS23980 |
| cds-WP_011746571.1 | *-* | 1.80288E-21 | 21 | 4.22184E-23 | 2.2 | up | sigma-54-dependent Fis family transcriptional regulator CDS | Pden_0224 | PDEN_RS01080 |
| cds-WP_011749101.1 | *fdhE* | 4.08026E-09 | 8 | 3.97233E-10 | 2.2 | up | fdhE CDS | Pden_2826 | PDEN_RS14070 |
| cds-WP_198140519.1 | *-* | 3.56041E-12 | 11 | 2.29281E-13 | 2.1 | up | Na/Pi symporter CDS | Pden_0816 | PDEN_RS04055 |
| cds-WP_011749131.1 | *fdhF* | 2.5938E-15 | 15 | 1.07981E-16 | 2.1 | up | formate dehydrogenase subunit alpha | Pden_2856 | PDEN_RS14220 |
| cds-WP_011749201.1 | *-* | 3.42724E-17 | 16 | 1.13696E-18 | 2.1 | up | L-lactate permease CDS | Pden_2927 | PDEN_RS14580 |
| cds-WP_011749696.1 | *-* | 3.18621E-23 | 22 | 6.494E-25 | 2.1 | up | multidrug efflux RND transporter permease subunit CDS | Pden_3448 | PDEN_RS17080 |
| cds-WP_011746900.1 | *-* | 2.84316E-37 | 37 | 2.65082E-39 | 2.1 | up | 2-oxoglutarate dehydrogenase E1 component CDS | Pden_0555 | PDEN_RS02770 |
| cds-WP_011746873.1 | *-* | 2.76775E-14 | 14 | 1.30826E-15 | 2.1 | up | BMP family ABC transporter substrate-binding protein CDS | Pden_0528 | PDEN_RS02630 |
| cds-WP_011748622.1 | *-* | 1.33414E-07 | 7 | 1.60548E-08 | 2.1 | up | 4-aminobutyrate--2-oxoglutarate transaminase CDS | Pden_2338 | PDEN_RS11610 |
| cds-WP_011749743.1 | *-* | 1.36678E-10 | 10 | 1.06687E-11 | 2.1 | up | TRAP transporter substrate-binding protein CDS | Pden_3497 | PDEN_RS17330 |
| cds-WP_011751040.1 | *-* | 4.57072E-13 | 12 | 2.61637E-14 | 2.1 | up | acyl-CoA synthetase CDS | Pden_4820 | PDEN_RS23995 |
| cds-WP_011749869.1 | *-* | 1.64513E-15 | 15 | 6.77741E-17 | 2.1 | up | isovaleryl-CoA dehydrogenase CDS | Pden_3633 | PDEN_RS18015 |
| cds-WP_011750239.1 | *-* | 9.57349E-08 | 7 | 1.12715E-08 | 2.1 | up | DUF2945 domain-containing protein CDS | Pden_4005 | PDEN_RS19945 |
| cds-WP_011747578.1 | *-* | 8.65655E-09 | 8 | 8.65279E-10 | 2.1 | up | SDR family oxidoreductase CDS | Pden_1255 | PDEN_RS06195 |
| cds-WP_011746872.1 | *-* | 9.5675E-09 | 8 | 9.60484E-10 | 2.1 | up | ABC transporter ATP-binding protein CDS | Pden_0527 | PDEN_RS02625 |
| cds-WP_041529795.1 | *-* | 9.51651E-07 | 6 | 1.33503E-07 | 2.1 | up | hypothetical protein CDS | Pden_0978 | PDEN_RS04875 |
| cds-WP_011747142.1 | *-* | 6.56117E-05 | 4 | 1.25334E-05 | 2.1 | up | ABC transporter permease CDS | Pden_0802 | PDEN_RS03990 |
| cds-WP_011750101.1 | *-* | 4.08146E-10 | 9 | 3.43366E-11 | 2.1 | up | zinc metallopeptidase CDS | Pden_3868 | PDEN_RS19245 |
| cds-WP_041529728.1 | *-* | 4.24381E-08 | 7 | 4.78487E-09 | 2.0 | up | hypothetical protein CDS | Pden_0347 | PDEN_RS01705 |
| cds-WP_011750741.1 | *-* | 3.14314E-10 | 10 | 2.58975E-11 | 2.0 | up | VOC family protein CDS | Pden_4516 | PDEN_RS22510 |
| cds-WP_128492954.1 | *-* | 5.9106E-10 | 9 | 5.10065E-11 | 2.0 | up | tyrosine-type recombinase/integrase CDS | Pden_2419 | PDEN_RS12025 |
| cds-WP_011746899.1 | *odhB* | 6.06734E-35 | 34 | 6.05156E-37 | 2.0 | up | 2-oxoglutarate dehydrogenase complex dihydrolipoyllysine-residue succinyltransferase | Pden_0554 | PDEN_RS02765 |
| cds-WP_104493368.1 | *-* | 9.22046E-12 | 11 | 6.26583E-13 | 2.0 | up | hypothetical protein CDS | Pden_0393 | PDEN_RS01940 |
| cds-WP_011749712.1 | *-* | 2.10941E-05 | 5 | 3.66814E-06 | 2.0 | up | Rieske 2Fe-2S domain-containing protein CDS | Pden_3465 | PDEN_RS17165 |
| cds-WP_011749575.1 | *-* | 3.78018E-07 | 6 | 4.94243E-08 | 2.0 | up | hypothetical protein CDS | Pden_3314 | PDEN_RS16455 |
| cds-WP_081465042.1 | *-* | 3.96294E-06 | 5 | 6.11799E-07 | 2.0 | up | rhomboid family intramembrane serine protease CDS | Pden_2337 | PDEN_RS11605 |
| cds-WP_041529953.1 | *-* | 1.12582E-09 | 9 | 1.0106E-10 | 2.0 | up | Arc family DNA-binding protein CDS | Pden_2281 | PDEN_RS26955 |
| cds-WP_164901601.1 | *-* | 5.09035E-05 | 4 | 9.54717E-06 | 2.0 | up | hypothetical protein CDS |  | PDEN_RS27300 |
| cds-WP_011747299.1 | *phaR* | 2.14211E-21 | 21 | 5.1091E-23 | 2.0 | up | polyhydroxyalkanoate synthesis repressor PhaR | Pden_0960 | PDEN_RS04775 |
| cds-WP_011748334.1 | *-* | 6.65088E-08 | 7 | 7.67188E-09 | -2.0 | down | TonB-dependent receptor CDS | Pden_2046 | PDEN_RS10170 |
| cds-WP_011747230.1 | *rpsF* | 1.62712E-26 | 26 | 2.54017E-28 | -2.0 | down | rpsF CDS | Pden_0891 | PDEN_RS04425 |
| cds-WP_011748873.1 | *-* | 1.56791E-13 | 13 | 8.2951E-15 | -2.0 | down | RNA-binding protein CDS | Pden_2593 | PDEN_RS12885 |
| cds-WP_011749934.1 | *-* | 3.25276E-07 | 6 | 4.18232E-08 | -2.0 | down | RNA methyltransferase CDS | Pden_3698 | PDEN_RS18360 |
| cds-WP_011748055.1 | *ilvC* | 2.75472E-10 | 10 | 2.25777E-11 | -2.0 | down | ketol-acid reductoisomerase | Pden_1760 | PDEN_RS08665 |
| cds-WP_011747229.1 | *-* | 7.58753E-13 | 12 | 4.53118E-14 | -2.0 | down | 30S ribosomal protein S18 CDS | Pden_0890 | PDEN_RS04420 |
| cds-WP_041530668.1 | *-* | 2.35576E-06 | 6 | 3.48869E-07 | -2.0 | down | hypothetical protein CDS | Pden_4302 | PDEN_RS21430 |
| cds-WP_041530731.1 | *-* | 6.49124E-19 | 18 | 1.88601E-20 | -2.0 | down | altronate dehydratase CDS | Pden_4928 | PDEN_RS24515 |
| cds-WP_010400236.1 | *rpsS* | 2.86461E-07 | 7 | 3.64597E-08 | -2.0 | down | 30S ribosomal protein S19 | Pden_0763 | PDEN_RS03790 |
| cds-WP_011748577.1 | *-* | 8.03332E-05 | 4 | 1.54849E-05 | -2.0 | down | glycosyl transferase CDS | Pden_2293 | PDEN_RS11390 |
| cds-WP_011747088.1 | *rplK* | 5.48385E-17 | 16 | 1.86679E-18 | -2.0 | down | 50S ribosomal protein L11 | Pden_0743 | PDEN_RS03695 |
| cds-WP_011747028.1 | *tolQ* | 9.96023E-13 | 12 | 6.04698E-14 | -2.0 | down | tolQ CDS | Pden_0683 | PDEN_RS03395 |
| cds-WP_011747109.1 | *rpsQ* | 1.96741E-07 | 7 | 2.423E-08 | -2.0 | down | 30S ribosomal protein S17 | Pden_0768 | PDEN_RS03815 |
| cds-WP_011750311.1 | *-* | 6.36432E-18 | 17 | 1.97333E-19 | -2.0 | down | 50S ribosomal protein L25/general stress proteinCtc CDS | Pden_4077 | PDEN_RS20310 |
| cds-WP_041530730.1 | *-* | 1.00529E-07 | 7 | 1.19099E-08 | -2.0 | down | amino acid ABC transporter permease CDS | Pden_4924 | PDEN_RS24495 |
| cds-WP_011750529.1 | *rpsD* | 7.40185E-13 | 12 | 4.39745E-14 | -2.1 | down | 30S ribosomal protein S4 | Pden_4303 | PDEN_RS21435 |
| cds-WP_011748890.1 | *-* | 2.60821E-06 | 6 | 3.89083E-07 | -2.1 | down | TonB-dependent receptor CDS | Pden_2610 | PDEN_RS12975 |
| cds-WP_104491135.1 | *-* | 5.30849E-09 | 8 | 5.23713E-10 | -2.1 | down | H-type lectin domain-containing protein CDS | Pden_3820 | PDEN_RS19005 |
| cds-WP_041529773.1 | *rpmC* | 7.36309E-13 | 12 | 4.35846E-14 | -2.1 | down | 50S ribosomal protein L29 | Pden_0767 | PDEN_RS03810 |
| cds-WP_011749035.1 | *rpsO* | 1.66471E-10 | 10 | 1.31387E-11 | -2.1 | down | 30S ribosomal protein S15 | Pden_2760 | PDEN_RS13740 |
| cds-WP_011750809.1 | *-* | 1.94114E-09 | 9 | 1.79299E-10 | -2.1 | down | co-chaperone GroES CDS | Pden_4586 | PDEN_RS22840 |
| cds-WP_011747105.1 | *rplV* | 3.39651E-10 | 9 | 2.82061E-11 | -2.1 | down | 50S ribosomal protein L22 | Pden_0764 | PDEN_RS03795 |
| cds-WP_011751150.1 | *-* | 1.12779E-05 | 5 | 1.86824E-06 | -2.1 | down | SDR family oxidoreductase CDS | Pden_4932 | PDEN_RS24535 |
| cds-WP_011747103.1 | *-* | 1.04265E-13 | 13 | 5.35793E-15 | -2.1 | down | 50S ribosomal protein L23 CDS | Pden_0761 | PDEN_RS03780 |
| cds-WP_010400243.1 | *rplN* | 1.79749E-20 | 20 | 4.55998E-22 | -2.1 | down | rplN CDS | Pden_0769 | PDEN_RS03820 |
| cds-WP_011747115.1 | *rplR* | 1.77872E-14 | 14 | 8.3305E-16 | -2.1 | down | rplR CDS | Pden_0775 | PDEN_RS03850 |
| cds-WP_041529977.1 | *-* | 4.64011E-11 | 10 | 3.42072E-12 | -2.1 | down | non-ribosomal peptide synthetase CDS | Pden_2564 | PDEN_RS12745 |
| cds-WP_011748559.1 | *rplT* | 1.39224E-17 | 17 | 4.43754E-19 | -2.1 | down | 50S ribosomal protein L20 | Pden_2273 | PDEN_RS11300 |
| cds-WP_011748041.1 | *ndk* | 9.24805E-22 | 21 | 2.12553E-23 | -2.1 | down | nucleoside-diphosphate kinase | Pden_1746 | PDEN_RS08590 |
| cds-WP_041530417.1 | *-* | 3.39964E-06 | 5 | 5.17465E-07 | -2.1 | down | hypothetical protein CDS |  | PDEN_RS19080 |
| cds-WP_041530427.1 | *-* | 1.52466E-09 | 9 | 1.37854E-10 | -2.1 | down | chorismate mutase CDS | Pden_3880 | PDEN_RS19305 |
| cds-WP_011747113.1 | *rpsH* | 1.50647E-19 | 19 | 4.08301E-21 | -2.1 | down | 30S ribosomal protein S8 | Pden_0773 | PDEN_RS03840 |
| cds-WP_011746353.1 | *mnmE* | 5.01848E-15 | 14 | 2.17627E-16 | -2.1 | down | tRNA uridine-5-carboxymethylaminomethyl(34) synthesis GTPase MnmE | Pden_0003 | PDEN_RS00020 |
| cds-WP_011747114.1 | *rplF* | 8.58302E-24 | 23 | 1.6377E-25 | -2.1 | down | 50S ribosomal protein L6 | Pden_0774 | PDEN_RS03845 |
| cds-WP_011750114.1 | *rpsP* | 1.87962E-27 | 27 | 2.64908E-29 | -2.1 | down | 30S ribosomal protein S16 | Pden_3881 | PDEN_RS19310 |
| cds-WP_011749243.1 | *rplM* | 7.8E-29 | 28 | 1.03166E-30 | -2.1 | down | 50S ribosomal protein L13 | Pden_2969 | PDEN_RS14790 |
| cds-WP_011750112.1 | *-* | 9.79608E-07 | 6 | 1.38063E-07 | -2.1 | down | GNAT family N-acetyltransferase CDS | Pden_3879 | PDEN_RS19300 |
| cds-WP_011749491.1 | *-* | 4.78384E-15 | 14 | 2.0434E-16 | -2.1 | down | enoyl-CoA hydratase/isomerase family protein CDS | Pden_3225 | PDEN_RS16020 |
| cds-WP_011747231.1 | *-* | 3.199E-10 | 9 | 2.64272E-11 | -2.2 | down | polyisoprenoid-binding protein CDS | Pden_0892 | PDEN_RS04430 |
| cds-WP_011749482.1 | *-* | 5.07436E-14 | 13 | 2.46998E-15 | -2.2 | down | NAD(P)-dependent oxidoreductase CDS | Pden_3256 | PDEN_RS16165 |
| cds-WP_011749481.1 | *-* | 4.4001E-16 | 15 | 1.62189E-17 | -2.2 | down | carboxymuconolactone decarboxylase family protein CDS | Pden_3257 | PDEN_RS16170 |
| cds-WP_011747030.1 | *tolA* | 1.28906E-12 | 12 | 7.88194E-14 | -2.2 | down | cell envelope integrity protein TolA | Pden_0685 | PDEN_RS03405 |
| cds-WP_011747029.1 | *-* | 9.12503E-11 | 10 | 6.90511E-12 | -2.2 | down | ExbD/TolR family protein CDS | Pden_0684 | PDEN_RS03400 |
| cds-WP_011750115.1 | *rimM* | 1.00562E-33 | 33 | 1.04661E-35 | -2.2 | down | 16S rRNA processing protein RimM | Pden_3882 | PDEN_RS19315 |
| cds-WP_011751144.1 | *-* | 1.27925E-06 | 6 | 1.8279E-07 | -2.2 | down | amino acid ABC transporter ATP-binding protein CDS | Pden_4926 | PDEN_RS24505 |
| cds-WP_011748418.1 | *-* | 4.72334E-10 | 9 | 4.02488E-11 | -2.2 | down | GNAT family N-acetyltransferase CDS | Pden_2131 | PDEN_RS10585 |
| cds-WP_011749734.1 | *-* | 4.17521E-06 | 5 | 6.48189E-07 | -2.2 | down | FAD-dependent monooxygenase CDS | Pden_3488 | PDEN_RS17285 |
| cds-WP_011747112.1 | *rpsN* | 6.33318E-15 | 14 | 2.80132E-16 | -2.2 | down | 30S ribosomal protein S14 | Pden_0772 | PDEN_RS03835 |
| cds-WP_011749475.1 | *dctP* | 2.00951E-08 | 8 | 2.11756E-09 | -2.2 | down | TRAP transporter substrate-binding protein DctP | Pden_3209 | PDEN_RS15945 |
| cds-WP_011746516.1 | *-* | 7.18256E-13 | 12 | 4.23603E-14 | -2.2 | down | cupin domain-containing protein CDS | Pden_0167 | PDEN_RS00805 |
| cds-WP_011750335.1 | *-* | 4.7603E-27 | 26 | 6.91545E-29 | -2.2 | down | GNAT family N-acetyltransferase CDS | Pden_4101 | PDEN_RS20440 |
| cds-WP_011746938.1 | *-* | 1.06554E-09 | 9 | 9.51867E-11 | -2.2 | down | hypothetical protein CDS | Pden_0593 | PDEN_RS02955 |
| cds-WP_011747080.1 | *-* | 4.42051E-16 | 15 | 1.639E-17 | -2.3 | down | dienelactone hydrolase family protein CDS | Pden_0735 | PDEN_RS03650 |
| cds-WP_011747110.1 | *-* | 3.64938E-15 | 14 | 1.54299E-16 | -2.3 | down | 50S ribosomal protein L24 CDS | Pden_0770 | PDEN_RS03825 |
| cds-WP_011750599.1 | *-* | 1.11461E-05 | 5 | 1.84275E-06 | -2.3 | down | TonB-dependent siderophore receptor CDS | Pden_4373 | PDEN_RS21785 |
| cds-WP_011747395.1 | *-* | 2.50982E-06 | 6 | 3.73317E-07 | -2.3 | down | hypothetical protein CDS | Pden_1066 | PDEN_RS05295 |
| cds-WP_011748329.1 | *-* | 1.05151E-31 | 31 | 1.20837E-33 | -2.3 | down | aspartate aminotransferase family protein CDS | Pden_2041 | PDEN_RS10145 |
| cds-WP_011749962.1 | *rpsB* | 2.85785E-26 | 26 | 4.58545E-28 | -2.3 | down | 30S ribosomal protein S2 | Pden_3726 | PDEN_RS18490 |
| cds-WP_011750900.1 | *-* | 3.40184E-07 | 6 | 4.4091E-08 | -2.3 | down | MaoC family dehydratase CDS | Pden_4680 | PDEN_RS23300 |
| cds-WP_011747117.1 | *rpmD* | 3.34279E-16 | 15 | 1.21042E-17 | -2.3 | down | 50S ribosomal protein L30 | Pden_0777 | PDEN_RS03860 |
| cds-WP_011750640.1 | *gabT* | 3.41155E-08 | 7 | 3.78733E-09 | -2.3 | down | 4-aminobutyrate--2-oxoglutarate transaminase | Pden_4414 | PDEN_RS21980 |
| cds-WP_019353022.1 | *groES* | 3.56599E-10 | 9 | 2.97681E-11 | -2.3 | down | co-chaperone GroES |  | PDEN_RS18130 |
| cds-WP_011749493.1 | *-* | 6.23656E-14 | 13 | 3.05608E-15 | -2.4 | down | CoA transferase CDS | Pden_3227 | PDEN_RS16030 |
| cds-WP_041530483.1 | *-* | 3.94797E-15 | 14 | 1.6778E-16 | -2.4 | down | DUF1775 domain-containing protein CDS | Pden_4444 | PDEN_RS22135 |
| cds-WP_011749303.1 | *-* | 6.3387E-06 | 5 | 1.00468E-06 | -2.4 | down | ABC transporter substrate-binding protein CDS | Pden_3033 | PDEN_RS15130 |
| cds-WP_011749492.1 | *-* | 2.61895E-16 | 16 | 9.31284E-18 | -2.4 | down | thiolase family protein CDS | Pden_3226 | PDEN_RS16025 |
| cds-WP_011747619.1 | *-* | 3.76874E-07 | 6 | 4.91931E-08 | -2.4 | down | DegT/DnrJ/EryC1/StrS family aminotransferase CDS | Pden_1296 | PDEN_RS06400 |
| cds-WP_011750312.1 | *trmFO* | 6.17576E-17 | 16 | 2.11572E-18 | -2.4 | down | methylenetetrahydrofolate--tRNA-(uracil(54)- C(5))-methyltransferase (FADH(2)-oxidizing) TrmFO | Pden_4078 | PDEN_RS20315 |
| cds-WP_011747084.1 | *-* | 6.95284E-27 | 26 | 1.04021E-28 | -2.4 | down | ABC transporter ATP-binding protein CDS | Pden_0739 | PDEN_RS03670 |
| cds-WP_011750569.1 | *cysT* | 2.98425E-06 | 6 | 4.48414E-07 | -2.4 | down | sulfate ABC transporter permease subunit CysT | Pden_4343 | PDEN_RS21630 |
| cds-WP_198140538.1 | *-* | 6.71859E-16 | 15 | 2.59304E-17 | -2.4 | down | AMP-binding protein CDS | Pden_3230 | PDEN_RS16045 |
| cds-WP_011751149.1 | *-* | 1.80674E-09 | 9 | 1.66101E-10 | -2.4 | down | zinc-binding alcohol dehydrogenase family protein CDS | Pden_4931 | PDEN_RS24530 |
| cds-WP_011749495.1 | *-* | 2.28546E-16 | 16 | 8.02786E-18 | -2.4 | down | MaoC family dehydratase N-terminal domain-containing protein CDS | Pden_3229 | PDEN_RS16040 |
| cds-WP_011747111.1 | *rplE* | 1.40972E-11 | 11 | 9.96462E-13 | -2.5 | down | rplE CDS | Pden_0771 | PDEN_RS03830 |
| cds-WP_011750669.1 | *-* | 3.24357E-15 | 14 | 1.36438E-16 | -2.5 | down | SCO family protein CDS | Pden_4443 | PDEN_RS22130 |
| cds-WP_011750945.1 | *-* | 5.97674E-07 | 6 | 8.06057E-08 | -2.5 | down | hypothetical protein CDS | Pden_4725 | PDEN_RS23520 |
| cds-WP_011749961.1 | *-* | 3.7282E-39 | 38 | 3.23348E-41 | -2.5 | down | elongation factor Ts CDS | Pden_3725 | PDEN_RS18485 |
| cds-WP_011746417.1 | *tnpB* | 2.53926E-08 | 8 | 2.74188E-09 | -2.5 | down | IS66 family insertion sequence element accessoryprotein TnpB | Pden_0067 | PDEN_RS00375 |
| cds-WP_011747189.1 | *-* | 4.2325E-25 | 24 | 7.43348E-27 | -2.5 | down | porin family protein CDS | Pden_0849 | PDEN_RS04220 |
| cds-WP_011746845.1 | *-* | 1.7296E-09 | 9 | 1.57884E-10 | -2.5 | down | VOC family protein CDS | Pden_0500 | PDEN_RS02485 |
| cds-PDEN_RS14835 | *-* | 9.02857E-06 | 5 | 1.47018E-06 | -2.5 | down | ? | Pden_2973 | PDEN_RS14835 |
| cds-WP_011751143.1 | *-* | 2.50718E-07 | 7 | 3.14757E-08 | -2.6 | down | amino acid ABC transporter permease CDS | Pden_4925 | PDEN_RS24500 |
| cds-WP_011750484.1 | *-* | 1.07338E-05 | 5 | 1.76646E-06 | -2.6 | down | Hsp70 family protein CDS | Pden_4255 | PDEN_RS21195 |
| cds-WP_011747262.1 | *-* | 5.76323E-16 | 15 | 2.16184E-17 | -2.6 | down | hypothetical protein CDS | Pden_0923 | PDEN_RS04585 |
| cds-WP_011747154.1 | *-* | 2.90382E-19 | 19 | 8.12215E-21 | -2.7 | down | phosphoglycerate dehydrogenase CDS | Pden_0814 | PDEN_RS04045 |
| cds-WP_104494388.1 | *-* | 1.52458E-06 | 6 | 2.2115E-07 | -2.7 | down | TetR family transcriptional regulator CDS | Pden_2940 | PDEN_RS14645 |
| cds-WP_011749494.1 | *-* | 8.69252E-21 | 20 | 2.12978E-22 | -2.7 | down | aldehyde dehydrogenase CDS | Pden_3228 | PDEN_RS16035 |
| cds-WP_011749216.1 | *-* | 1.0367E-39 | 39 | 8.5418E-42 | -2.8 | down | multidrug efflux RND transporter permease subunit CDS | Pden_2942 | PDEN_RS14655 |
| cds-WP_011747531.1 | *ureC* | 3.40184E-07 | 6 | 4.41089E-08 | -2.8 | down | urease subunit alpha | Pden_1208 | PDEN_RS05985 |
| cds-WP_041529781.1 | *-* | 2.97041E-07 | 7 | 3.78992E-08 | -2.8 | down | hypothetical protein CDS |  | PDEN_RS04285 |
| cds-WP_011748469.1 | *-* | 7.60771E-15 | 14 | 3.38157E-16 | -2.9 | down | DUF1800 domain-containing protein CDS | Pden_2182 | PDEN_RS10840 |
| cds-WP_011747118.1 | *-* | 1.8687E-08 | 8 | 1.95703E-09 | -2.9 | down | hypothetical protein CDS | Pden_0778 | PDEN_RS03865 |
| cds-WP_128492943.1 | *-* | 4.32341E-19 | 18 | 1.22803E-20 | -2.9 | down | ammonium transporter CDS | Pden_2032 | PDEN_RS10105 |
| cds-WP_011749215.1 | *-* | 1.18288E-07 | 7 | 1.41507E-08 | -3.0 | down | efflux RND transporter periplasmic adaptor subunit CDS | Pden_2941 | PDEN_RS14650 |
| cds-WP_011750899.1 | *-* | 1.09018E-23 | 23 | 2.10377E-25 | -3.0 | down | 6,7-dimethyl-8-ribityllumazine synthase CDS | Pden_4679 | PDEN_RS23295 |
| cds-WP_011750642.1 | *-* | 1.61074E-38 | 38 | 1.46685E-40 | -3.0 | down | NAD-dependent succinate-semialdehyde dehydrogenase CDS | Pden_4416 | PDEN_RS21990 |
| cds-WP_011750125.1 | *pdhA* | 2.83234E-40 | 40 | 2.27226E-42 | -3.2 | down | pyruvate dehydrogenase (acetyl-transferring) E1 component subunit alpha | Pden_3892 | PDEN_RS19370 |
| cds-WP_011749301.1 | *-* | 2.84838E-06 | 6 | 4.26145E-07 | -3.3 | down | iron ABC transporter permease CDS | Pden_3031 | PDEN_RS15120 |
| cds-WP_011750124.1 | *-* | 3.9234E-44 | 43 | 2.63715E-46 | -3.4 | down | pyruvate dehydrogenase complex E1 component subunit beta CDS | Pden_3891 | PDEN_RS19365 |
| cds-WP_011750123.1 | - | 1.41783E-52 | 52 | 7.68553E-55 | -3.5 | down | pyruvate dehydrogenase complex dihydrolipoamide acetyltransferase CDS | Pden_3890 | PDEN_RS19360 |
| cds-WP_011749300.1 | - | 4.60306E-09 | 8 | 4.50125E-10 | -3.5 | down | class I SAM-dependent methyltransferase CDS | Pden_3030 | PDEN_RS15115 |
| cds-WP_011748558.1 | - | 3.00914E-05 | 5 | 5.38278E-06 | -3.6 | down | cytochrome P450 CDS | Pden_2272 | PDEN_RS11295 |
| cds-WP_011747493.1 | - | 1.13413E-05 | 5 | 1.88119E-06 | -4.1 | down | ABC transporter substrate-binding protein CDS | Pden_1168 | PDEN_RS05785 |
| cds-WP_011748511.1 | - | 5.21305E-57 | 56 | 2.48671E-59 | -4.1 | down | NADP-dependent malic enzyme CDS | Pden_2224 | PDEN_RS11050 |
| cds-WP_164901616.1 | - | 8.65261E-35 | 34 | 8.81771E-37 | -4.4 | down | TonB-dependent siderophore receptor CDS | Pden_3029 | PDEN_RS15110 |
| cds-WP_011750985.1 | - | 1.2001E-101 | 101 | 3.1225E-104 | -9.6 | down | dicarboxylate/amino acid:cation symporter CDS | Pden_4765 | PDEN_RS23720 |

Supplementary Table 4: Top regulated genes KO_Suc_/WT_Suc_. Listed are all genes regulated above a threshold of log_2_-fold 2.0 in Pd1222 ΔramB on succinate compared to the Pd1222 wild type on succinate.

| gene_id | Gene | padj | neg. log10(padjust) | pvalue | log2FoldChange | regulation | CDS/product description | Old locus tag | New locus tag |
| --- | --- | --- | --- | --- | --- | --- | --- | --- | --- |
| cds-WP_011747683.1 | *aceB* | 3.44E-27 | 26.46 | 1.E-30 | 4.87 | up | malate synthase | Pden_1364 | PDEN_RS06730 |
| cds-WP_086000156.1 | *aceA* | 5.82E-65 | 64.23 | 1.E-68 | 4.42 | up | isocitrate lyase | Pden_1363 | PDEN_RS06725 |
